# Piezo1 activation attenuates thrombin-induced blebbing in breast cancer cells

**DOI:** 10.1242/jcs.258809

**Published:** 2022-04-01

**Authors:** Paul O'Callaghan, Adam Engberg, Olle Eriksson, Nikos Fatsis-Kavalopoulos, Christina Stelzl, Gonzalo Sanchez, Olof Idevall-Hagren, Johan Kreuger

**Affiliations:** 1 Department of Medical Cell Biology, Uppsala University, 75123 Uppsala, Sweden; 2 Department of Medical Biochemistry and Microbiology, 75123 Uppsala University, Uppsala, Sweden

**Keywords:** Piezo1, Thrombin, Blebbing, Breast cancer

## Abstract

Cancer cells exploit a variety of migration modes to leave primary tumors and establish metastases, including amoeboid cell migration, which is typically reliant on bleb formation. Here we demonstrate that thrombin induces dynamic blebbing in the MDA-MB-231 breast cancer cell line and confirm that protease-activated receptor 1 (PAR1) activation is sufficient to induce this effect. Cell confinement has been implicated as a driving force in bleb-based migration. Unexpectedly, we found that gentle contact compression, exerted using a custom built ‘cell press’ to mechanically stimulate cells, reduced thrombin-induced blebbing. Thrombin-induced blebbing was similarly attenuated using the small molecule Yoda1, an agonist of the mechanosensitive Ca^2+^ channel Piezo1, and this attenuation was impaired in Piezo1-depleted cells. Additionally, Piezo1 activation suppressed thrombin-induced phosphorylation of ezrin, radixin and moesin (ERM) proteins, which are implicated in the blebbing process. Our results provide mechanistic insights into Piezo1 activation as a suppressor of dynamic blebbing, specifically that which is induced by thrombin.

## Abstract (precis)

**Summary:** Thrombin and protease-activated receptor agonists induce dynamic blebbing in breast cancer cells, which can be attenuated by contact-mediated compression, and activation of the mechanosensitive ion channel Piezo1.

## INTRODUCTION

Cancer cells adopt a variety of migratory modes during metastasis, influenced in part by the composition of the local extracellular matrix (Tozluoğlu et al., 2013; Bergert et al., 2012). The amoeboid mode of migration is in part defined by the pressure-driven expansion and actomyosin-mediated retraction of plasma membrane (PM) protrusions, termed blebs. Bleb-driven cell migration is observed in a number of *in vivo* settings including germ cell migration during zebrafish embryogenesis (Blaser et al., 2006; Ruprecht et al., 2015) and in human cancer cells in models of tumorigenesis (Tozluoğlu et al., 2013). Bleb formation involves an expansion of the PM, triggered by its dissociation from the underlying actin cortex, causing it to balloon outward due to hydrostatic pressure (Charras et al., 2008). Ezrin, radixin and moesin (ERM) proteins, which tether actin to the PM, are among the first components to be recruited to the cytoskeleton-free bleb membrane, followed by actin and then myosin, which completes assembly of the contractile machinery required to retract the bleb membrane (Charras et al., 2006; Aoki et al., 2016). ERMs typically increase PM stability by coupling it to the actin cortex, which is associated with reduced blebbing capacity (Diz-Muñoz et al., 2010; Sliogeryte et al., 2014). The potential for mechanical confinement to trigger cells to adopt blebbing phenotypes and amoeboid modes of migration has been extensively studied and provides a basis for cell movement through matrices with reduced dependence on focal adhesions (Tozluoğlu et al., 2013; Logue et al., 2015; Liu et al., 2015; Ruprecht et al., 2015).

Tumor vascularization is central to the expansion of the primary malignancy, but is also the conduit through which cancer cells escape and establish metastases. Tumor vessels are often leaky and the tumor environment has been described as being in a pathological state of coagulation, which might be exacerbated by certain chemotherapies (Noble and Pasi, 2010). Consequently, cancer cells can exist within, and contribute to, a milieu enriched in various components of the coagulation cascade such as thrombin (Chaari et al., 2014; reviewed in Reddel et al., 2019). Thrombin acts on the G protein-coupled protease-activated receptors (PARs) 1–4 (Lin and Trejo, 2013; Mihara et al., 2016; Heuberger et al., 2019; Shapiro et al., 2000; Ishihara et al., 1997) to irreversibly sever a part of their N-terminal extracellular domain, thereby exposing a tethered ligand which activates the receptor through an intramolecular interaction. Thrombin activation of PARs results in phospholipase-C β (PLCβ)-mediated release of inositol 1,4,5-trisphosphate (IP3) from phosphatidylinositol 4,5-bisphosphate (PIP2), leaving diacylglycerol (DAG) in the PM, which can bind protein kinase C (PKC). Binding of IP3 to IP3 receptors (IP3R) in the endoplasmic reticulum (ER) triggers release of stored Ca^2+^, which in turn activates the DAG-bound PKC (reviewed in Coughlin, 2000). Ca^2+^ acts in consort with a variety of binding partners and relevant here is the Ca^2+^/calmodulin (CaM)-mediated activation of myosin light chain kinase (MLCK), which phosphorylates myosin II, permitting it to bind and contract actin filaments (Kamm and Stull, 2001). Myosin activity is fundamental to the contractility of cellular cytoskeletons and actomyosin contraction is an established driver of blebbing through its capacity to increase hydrostatic pressure within the cell (Tinevez et al., 2009). At the same time, actomyosin contractility is essential for the retraction of PM blebs (Charras et al., 2008).

Piezo1 is a mechanosensitive cation channel (Coste et al., 2010, 2012) implicated in diverse areas of cell biology, including shear stress sensing in endothelial cells (Li et al., 2014), regulation of red blood cell volume (Cahalan et al., 2015), cell division in epithelial cells (Gudipaty et al., 2017) and as a confinement sensor to optimize cell motility (Hung et al., 2016). Piezo1 is found in the plasma membrane as a homotrimer and, when in closed conformation, induces a bowl-shaped indentation in the PM. In response to increased membrane tension, Piezo1 transitions to a more planar arrangement and gates Ca^2+^ influx (Lin et al., 2019; Guo and MacKinnon, 2017). Furthermore, loss of cortical actin, which is also seen during the initial expansion of PM blebs, reduces the activation threshold of Piezo1 (Cox et al., 2016). Importantly, other mechanosensitive ion channels including the transient receptor potential vanilloid-type 4 (TRPV4) and the transient receptor potential cation channel subfamily M member 7 (TRPM7) have also been attributed important functional roles in cellular confinement sensing (Agarwal et al., 2021; Zhao et al., 2019, 2021).

Here, we demonstrate that thrombin induces blebbing in breast cancer cells, and that contact compression or stimulation with the small molecule Yoda1, a Piezo1 agonist, can suppress dynamic blebbing. Thrombin-induced blebbing was associated with increased ERM phosphorylation that was suppressed or reversed through Piezo1 activation and this effect was impaired when PP1 and PP2A serine/threonine phosphatases were inhibited. We propose that Piezo1 activation promotes dephosphorylation of ERMs, which in turn reduces the actomyosin contraction forces applied to the PM and thereby attenuates blebbing.

## RESULTS

### MDA-MB-231 breast cancer cells exhibit spontaneous blebbing

The current study was carried out using the MDA-MB-231 breast cancer cell model, and live imaging of MDA-MB-231 cells showed that a subpopulation of these cells exhibited spontaneous blebbing under standard cell culture conditions (Fig. S1A). F-actin dynamics were visualized by labeling cells with the live imaging probe SiR-actin, while changes in cell morphology were simultaneously monitored with differential interference contrast (DIC) microscopy and time-lapse imaging (Fig. S1B,C, Movie 1). In line with previous reports of bleb dynamics (Charras et al., 2005, 2008), new actin-free blebs expanded quickly (typically within 30 s), followed by actin recruitment and bleb retraction within approximately 2 min. *z*-stack analysis of SiR-actin distribution demonstrated that blebs could form over the majority of the cell surface, but were less prominent close to the apical and basal cell membranes (Fig. S1D).

### Thrombin induces blebbing in MDA-MB-231 cells

**Fig. 1.**
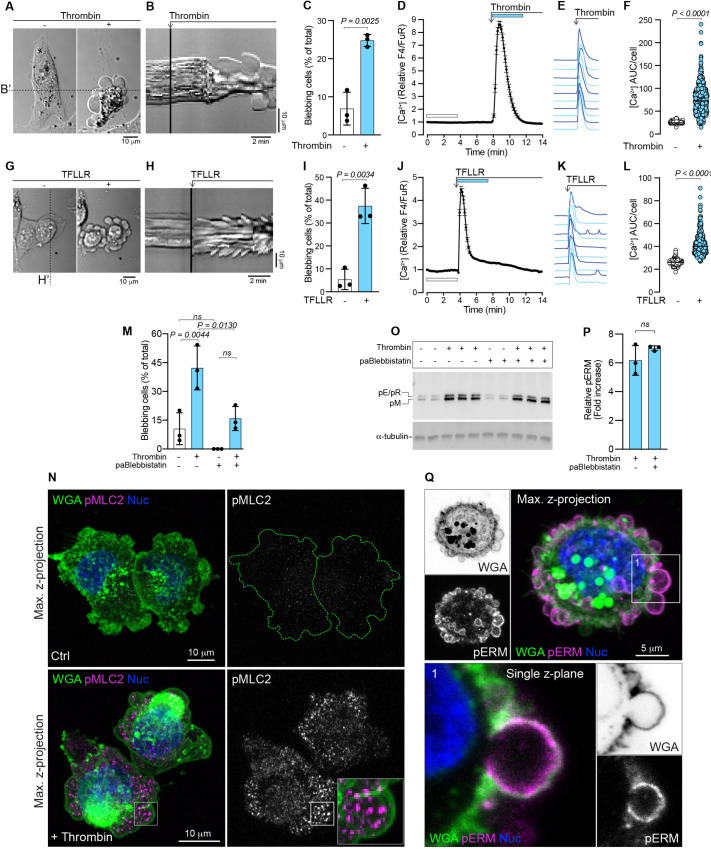
**Thrombin stimulation and PAR1 activation induces blebbing in MDA-MB-231 cells.** (A) Representative example of thrombin-induced blebbing in an MDA-MB-231 cell (see also Movie 2). The cell is presented before (left) and after (right) the addition of thrombin. Scale bar: 10 µm. (B) Kymograph plotted from the time-lapse signals recorded under the dashed line B′ in A; the point at which thrombin is added is indicated. Scale bars: 2 min (horizontal), 10 µm (vertical). (C) Quantification of blebbing in MDA-MB-231 cell populations before and after thrombin treatment. Bars represent the mean±s.d. from three experiments. (D) Thrombin-induced changes in cytosolic Ca^2+^ in MDA-MB-231 cells as quantified by ratiometric measurements of relative Fluo-4/Fura Red (F4/FuR) fluorescence. The mean values±s.e.m. over time for *n*=119 cells are plotted. (E) Examples of thrombin-induced Ca^2+^ responses from individual cells from the data set in D. (F) Quantification of Ca^2+^ area under the curve (AUC) per cell calculated from the relative F4/FuR plots for the durations indicated by the color-coded bars in D before and after thrombin treatment (*n*=593 cells, from the three experiments in C). (G) Induction of blebbing by TFLLR stimulation in an MDA-MB-231 cell. The cell is presented before (left) and after (right) the addition of TFLLR. Scale bar: 10 µm. (H) Kymograph plotted from the signal recorded under the dashed line H′ in panel G, the point at which TFLLR is added is indicated. Scale bars: 2 min (horizontal), 10 µm (vertical). (I) Quantification of blebbing in MDA-MB-231 cell populations before and after TFLLR treatment. Bars represent the mean±s.d. from three experiments. (J) Effect of TFLLR-treatment on cytosolic Ca^2+^. The plot represents the mean±s.e.m. over time for *n*=95 cells from one experiment. (K) Examples of TFLLR-induced Ca^2+^ responses from individual cells from the data set in J. (L) Quantification of Ca^2+^ AUC/cell for the durations indicated by the color-coded bars in J (*n*=392 cells from the three experiments in I). (M) Quantification of blebbing in MDA-MB-231 cell populations before and after thrombin treatment, and with or without pre-treatment (1 h) with para-aminoblebbistatin (paBlebbistatin). Bars represent the mean±s.d. for three experiments. (N) Maximum intensity *z*-projection images, collected by confocal microscopy, of control (Ctrl) and thrombin treated (+Thrombin) MDA-MB-231 cells immunostained for pMLC2 and counterstained with WGA for the plasma membrane, and NucBlue (Nuc) for nuclei. The peripheries of the pMLC2-stained Ctrl cells are outlined with a dashed green line. The inset in the lower right panel encloses a single bleb and illustrates pMLC2 distribution relative to the bleb membrane. Scale bars: 10 µm. (O) Immunoblotting for pERMs (pE, phospho-ezrin; pR, pospho-radixin; pM, phospho-moesin) in MDA-MB-231 cells pre-treated (1 h) with or without para-aminoblebbistatin (20 µM), followed by thrombin (1 U/ml) exposure for 5 min. α-tubulin was used as a loading control. The sample lanes for each condition represent biological replicates from the same experiment. (P) Quantification of pERM levels relative to α-tubulin, induced by thrombin with and without para-aminoblebbistatin pre-treatment from the blots in O; bars represent the mean±s.d. (Q) Maximum intensity *z*-projection image, collected by confocal microscopy, of a thrombin-treated MDA-MB-231 cell immunostained for pERMs and counterstained with WGA and NucBlue. A single *z*-plane from region 1, which is framed and enlarged, illustrates enrichment of pERMs associated with the bleb membrane. Scale bar: 5 µm. The *P*-values were determined by using an unpaired two-tailed Student's *t*-test in C, I and P, one-way ANOVA with Tukey's multiple comparisons in M, and Wilcoxon matched-pairs signed rank test in F and L. ns, not significant.

Tumor environments are typically thrombotic (Noble and Pasi, 2010) and a compilation of immunostaining data from the Human Protein Atlas (Uhlén et al., 2015; http://www.proteinatlas.org) demonstrates that thrombin is often present in breast cancer tissue (Fig. S2A,B). Thrombin induces actomyosin contraction (Gavara et al., 2006; Satpathy et al., 2004; Essler et al., 1998), which is also a driving force for bleb formation (Tinevez et al., 2009). Therefore, we tested the effect of thrombin on MDA-MB-231 breast cancer cells and found that it increased the number of blebbing cells by approximately 3-fold (Fig. 1A–C; Movie 2). Thrombin activation of PARs triggers Ca^2+^ release from the ER, which increases the potential for actomyosin contraction. We therefore imaged changes in cytosolic Ca^2+^ as a readout for thrombin signaling. Thrombin exposure induced a transient increase in cytosolic Ca^2+^, with similar kinetics observed in individual cells (Fig. 1D–F). To confirm that the bleb-inducing effects of thrombin were not related to its general activity as a protease, potentially promoting blebbing through cleavage of cell surface adhesion proteins, we assessed the effects of specific PAR agonists. PAR1 (also known as F2R) is the canonical thrombin receptor, and exposure of MDA-MB-231 cells to the PAR1 agonist peptide TFLLR mimicked the effects of thrombin with respect to bleb induction (Fig. 1G–I) and similarly increased cytosolic Ca^2+^ (Fig. 1J–L). Notably, some minor oscillations in the cytosolic Ca^2+^ levels over time were observed in individual cells stimulated with TFLLR (Fig. 1K). Further, the PAR2 agonist peptide SLIGRL also induced blebbing and transiently increased the levels of cytosolic Ca^2+^ (Fig. S2C–G), with some cells also displaying low amplitude oscillations after the initial peak of cytosolic Ca^2+^. Importantly, although blebbing behavior can also be indicative of cells undergoing apoptosis, cell viability analyses confirmed that even prolonged thrombin treatment (5 h) was not associated with increased cell death (Fig. S3A,B). Taken together, our data show that thrombin, as well as PAR1 and PAR2 agonists, induce blebbing in MDA-MB-231 breast cancer cells.

### Thrombin requires myosin II activity to induce blebbing, and stimulates phosphorylation of ERM proteins

To assess the role of actomyosin-mediated contraction in thrombin-induced blebbing, cells were pre-treated with the myosin II inhibitor para-aminoblebbistatin, which suppressed spontaneous blebbing and impaired the effect of thrombin on inducing blebbing (Fig. 1M), but did not reduce the thrombin-induced increase of cytosolic Ca^2+^ (Fig. S2H). Immunostaining for phosphorylated (p) myosin light chain 2 (MLC2, also known as MYL2) revealed elevated levels in thrombin-treated cells compared with untreated control cells (Fig. 1N). pMLC2 could be located within the cytosolic volume of the bleb but was not clearly associated with the bleb membrane (Fig. 1N, insert in lower right panel). Other potentially important regulators of blebbing are the ERM proteins that crosslink the actin cortex to the plasma membrane, to mediate mechanical forces exerted by myosin that can modulate intracellular pressure and blebbing (Diz-Muñoz et al., 2010; Sliogeryte et al., 2014). Thrombin stimulation was shown to upregulate ERM phosphorylation which was not affected by myosin II inhibition (Fig. 1O,P). Immunostaining for phosphorylated ERMs (pERMs) revealed that they were predominantly concentrated in the bleb membranes in thrombin-stimulated cells (Fig. 1Q and Fig. S5D). A similar pattern of pERM association was found in the bleb membranes of spontaneously blebbing control cells, whereas adjacent non-blebbing cells displayed considerably less pERM staining (Fig. S5C). Together, these data demonstrate that thrombin-induced blebbing is a myosin-dependent process, which is accompanied by phosphorylation of ERM proteins.

### Contact compression attenuates spontaneous blebbing in MDA-MB-231 cells

**Fig. 2.**
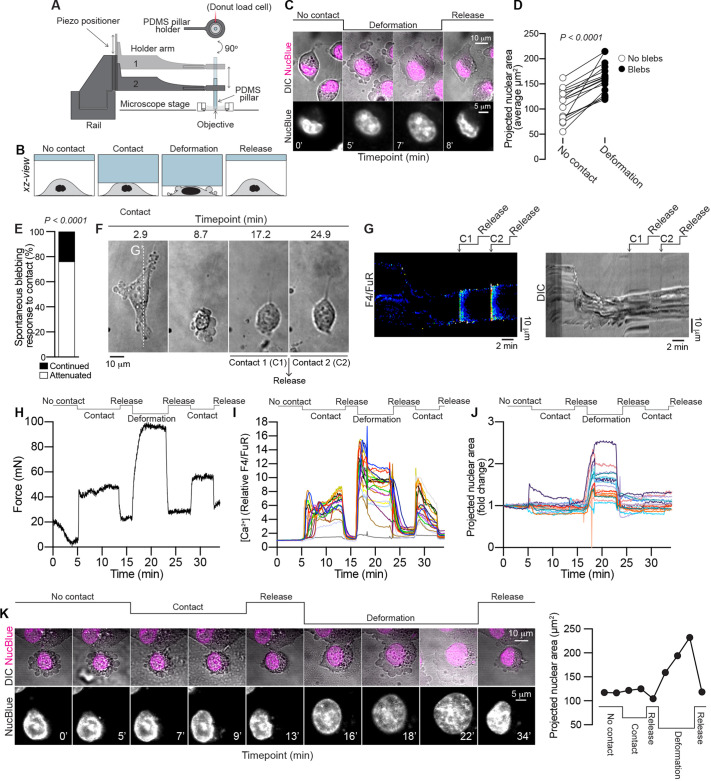
**Contact compression attenuates spontaneous blebbing.** (A) Overview of the cell press setup mounted on a confocal microscope stage. MDA-MB-231 cells were subjected to compression using a PDMS pillar attached to a vertical piezoelectric track. Force measurements were recorded using a Futek donut load cell. (B) Cells were imaged during no contact, contact compression, deformation compression, and after release of compression. The cartoon presents a side-on view (*xz*-view) of the cell during the different compression states. (C) Time-lapse microscopy of cell (DIC) and nucleus (NucBlue) morphological changes during deformation-induced bleb formation and following release of compression. Scale bars: 10 µm (top), 5 µm (bottom). (D) Quantitative analysis of the projected nuclear area of individual cells before and after deformation-induced bleb formation (*n*=17 cells)*.* The *P*-value was determined by paired two-tailed Student's *t-*test. (E) Quantitative analysis of contact-mediated attenuation of spontaneous blebbing (*n*=21 cells). The *P*-value was determined by Fisher's exact test. (F) Time-lapse images of bleb attenuation following contact compression (C1). Blebbing did not reoccur following release of compression or by a second gentle contact compression event (C2). Scale bar: 10 µm. (G) Kymographs of the changes in cytosolic Ca^2+^ (left) and cell morphology (right), recorded by confocal and DIC microscopy, respectively. Signals were plotted from under the vertical dashed line G′ in panel F. See Materials and Methods for details of how the ratiometric images were prepared. Scale bars: 2 min (horizontal), 10 µm (vertical). (H) Force measurements during the indicated states of contact compression, deformation compression and release. (I) Changes in cytosolic Ca^2+^ during the different compressive states outlined above the traces. (J) Changes in projected nuclear area during the different compressive states outlined above the traces. Individual traces from a sample of *n*=20 cells from one experiment are shown in I and J. (K) Time-lapse imaging of a blebbing MDA-MB-231 cell in which blebbing was first attenuated by contact compression, after which compression was released and the cell was subsequently subjected to deformation compression, which induced bleb formation, which was reversed upon release of compression. The projected nuclear area measurements are shown to the right. Scale bars: 10 µm (top), 5 µm (bottom).

Cellular confinement has been implicated as a driving force in the induction of blebbing (Liu et al., 2015; Logue et al., 2015; Tozluoğlu et al., 2013; Ruprecht et al., 2015). Two recent studies implicate the nucleus as an important mechanosensory organelle, demonstrating that compression-induced stretching of the nuclear membrane triggers a myosin-dependent contractile reaction that drives blebbing (Lomakin et al., 2020; Venturini et al., 2020). Based on the capacity of thrombin to induce blebbing in MDA-MB-231 cells (Fig. 1), we next hypothesized that blebbing might be enhanced if cells are exposed to thrombotic, spatially restricted environments. We therefore set out to assess the combined effects of thrombin and physical confinement on blebbing. For this purpose, MDA-MB-231 cells were compressed using a custom-built ‘cell press’, composed of a polydimethylsiloxane (PDMS) pillar positioned along a linear piezoelectric track mounted to a confocal microscope stage, permitting live-cell imaging during compression (Fig. 2A). Cells and nuclei were imaged (1) prior to compression; (2) during contact compression; (3) during compression leading to nuclear deformation; and (4) following compression release (Fig. 2B). A blebbing response was induced in MDA-MB-231 cells following cellular compression that led to deformation of the nucleus, measured as an increase in the projected nuclear area (Fig. 2C,D), which was reversed once the compression was released (Fig. 2C). This indicated that the recently defined ‘nuclear ruler’ function is active in MDA-MB-231 cells (Lomakin et al., 2020; Venturini et al., 2020). However, we unexpectedly observed that spontaneous blebbing could be attenuated following initial gentle contact compression with the cell press (Fig. 2E,F). Notably, this contact-attenuation of blebbing was associated with an increase in cytosolic Ca^2+^ (Fig. 2G) and blebbing did not resume when compression was released, nor was blebbing reinstated by subjecting the cell to a secondary contact compression event, which similarly induced an increase in cytosolic Ca^2+^ (Fig. 2G). To assess the differences in force exerted during initial contact compression and deformation compression, the cell press tool was fitted with a Futek LTH300 donut load cell. The pillar was lowered onto the cells in 5 µm steps. Upon first contact compression of the cell layer, the load cell registered an increase in force (Fig. 2H), which was accompanied by an increase in cytosolic Ca^2+^ (Fig. 2I). The pillar was then released and cytosolic Ca^2+^ levels returned to the baseline. The pillar was next lowered past the previous contact compression position in 2 µm steps, until the cells were visibly deformed and many cells were observed to form blebs. The load cell confirmed an increase in force during the deformation compression procedure as compared to contact compression (Fig. 2H), and an increase in cytosolic Ca^2+^ also accompanied the deformation phase (Fig. 2I). The pillar was released and the cells were subjected to a final round of contact compression, which again increased cytosolic Ca^2+^ up until the point of release (Fig. 2I). Analyses of changes to the projected nuclear area of cells revealed a pronounced increase during the deformation phase, compared to the contact or release phases (Fig. 2J). Note that transient spikes in the recordings in Fig. 2I,J during the deformation phase are attributed to focus adjustments. Spontaneous blebbing in MDA-MB-231 cells that was attenuated by contact could also be pushed into blebbing behavior by deformation compression, which led to an increase in the projected nuclear area, which again ceased upon release of compression (Fig. 2K). Taken together, these results demonstrate that different degrees of compression can exert opposing effects on the cells’ capacity to form blebs, such that gentle contact compression can attenuate blebbing, whereas severe compression that causes deformation of the cell body and nucleus leads to the induction of blebbing.

### Contact compression attenuates thrombin-induced blebbing in MDA-MB-231 cells

**Fig. 3.**
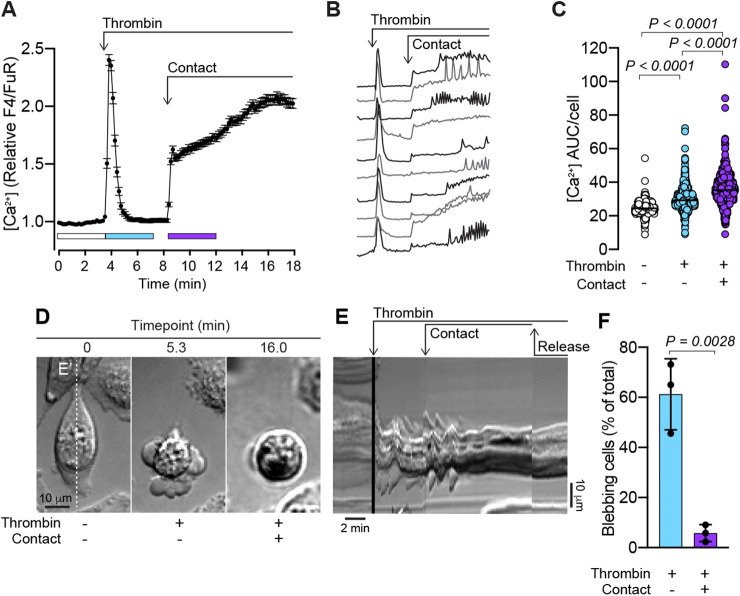
**Contact compression attenuates thrombin-induced blebbing.** (A) Thrombin- (1 U/ml) and contact-induced changes in cytosolic Ca^2+^ in MDA-MB-231 cells. The plot represents the mean±s.e.m. over time (*n*=163 cells, from one experiment). (B) Examples of individual thrombin- and contact-induced Ca^2+^ responses from individual MDA-MB-231 cells. (C) Quantification of Ca^2+^ AUC/cell for the durations indicated by the color-coded bars in A for *n*=556 cells from three experiments. (D) Time-lapse of an MDA-MB-231 cell illustrating thrombin-induced blebbing and its attenuation by gentle contact compression (see also Movie 3). Scale bar: 10 µm. (E) Kymograph of the time-lapse sequence plotted from under the vertical dashed line E′ in panel D. Timepoints for thrombin addition, contact compression and release of compression are annotated. Scale bars: 2 min (horizontal), 10 µm (vertical). (F) Quantification of blebbing in MDA-MB-231 cell populations in the presence of thrombin before and after contact compression. Bars represent the mean±s.d., for the same three experiments analyzed in C. The *P*-values were determined by using the Friedman test with Dunn's multiple comparisons in C, and by unpaired two-tailed Student's *t-*test in F.

Next, the effects of gentle contact compression on thrombin-induced blebbing were evaluated. Following the thrombin-induced Ca^2+^ response and associated blebbing, the compression pillar of the cell press was lowered until a contact-induced increase in cytosolic Ca^2+^ was recorded (Fig. 3A–C). Interestingly, in some cells the contact compression-induced elevation of cytosolic Ca^2+^ exhibited oscillations (Fig. 3B). As in the case of spontaneous blebbing, thrombin-induced blebbing could also be attenuated by contact compression (Fig. 3D), and the attenuation of blebbing persisted after contact compression was released (Fig. 3E; Movie 3). Quantitative assessment of the blebbing in thrombin-stimulated MDA-MB-231 cells before and after contact compression confirmed that contact significantly attenuated blebbing (Fig. 3F).

### Piezo 1 is expressed in MDA-MB-231 cells and its agonist Yoda1 attenuates thrombin-induced blebbing

**Fig. 4.**
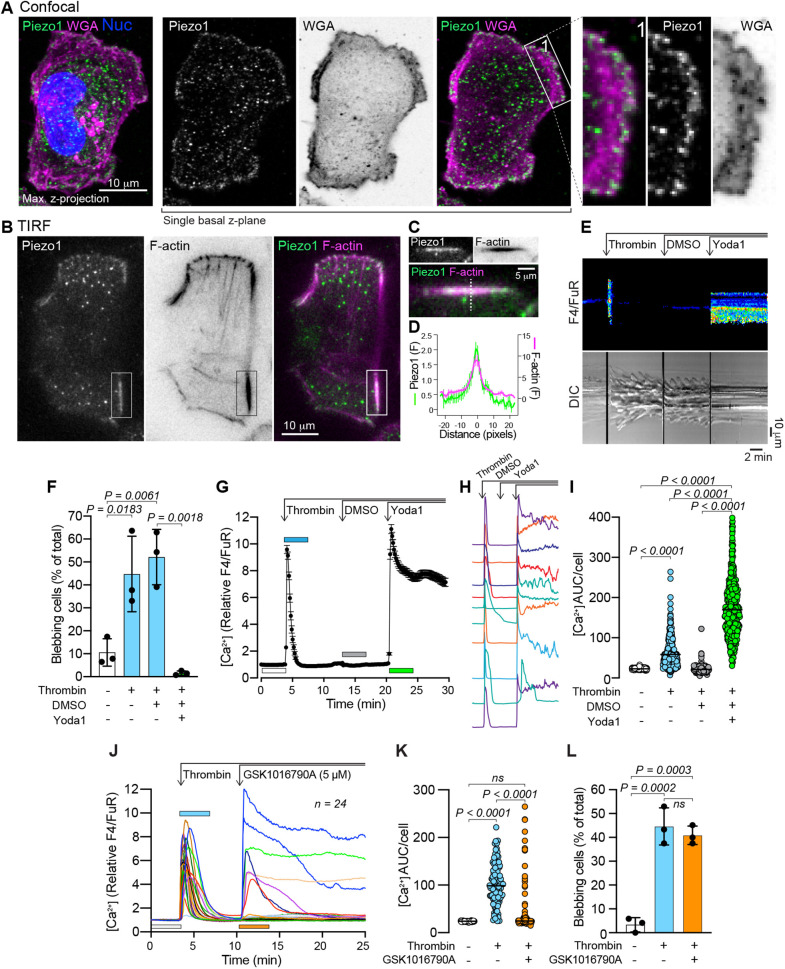
**Piezo1 activation by Yoda1 attenuates thrombin-induced blebbing.** (A) Maximum intensity *z*-projection image, collected by confocal microscopy, of an MDA-MB-231 cell immunostained for Piezo1 and counterstained with WGA and NucBlue. A single *z*-plane from the same *z*-stack reveals Piezo1 distribution in the most basal region of the plasma membrane, and the framed and enlarged region 1 illustrate the presence of Piezo1 enrichment at the periphery of the cell. Scale bar: 10 µm. (B) TIRF microscopy images of a cell stained for Piezo1 and F-actin. Scale bar: 10 µm. (C) Enlarged and rotated view of the area enclosed by the rectangle in panel B. Scale bar: 5 µm. (D) Relative fluorescence intensity profile of Piezo1 and F-actin staining under linear regions of interest similar to that indicated by the dashed line in C, i.e. plotted transversely through F-actin filaments. The plot represents the mean±s.e.m. from 11 structures from three cells. (E) Kymographs of the changes in cytosolic Ca^2+^ (top) and cell morphology (bottom) before and after sequential addition of thrombin, DMSO and Yoda1 in a single MDA-MB-231 cell, recorded by confocal and DIC microscopy (see also Movie 4). Scale bars: 2 min (horizontal), 10 µm (vertical). (F) Quantification of blebbing in MDA-MB-231 cell populations before and after sequential addition of thrombin, DMSO, and Yoda1. Bars represent the mean±s.d. from three experiments. (G) Changes to cytosolic Ca^2+^ (relative F4/FuR ratio) in cells before and after thrombin (1 U/ml), DMSO and Yoda1 (20 µM) addition. The plot represents the mean±s.e.m. over time (*n*=148 cells). (H) Examples of individual Ca^2+^ responses during sequential thrombin, DMSO and Yoda1 treatment from individual cells. (I) Quantification of Ca^2+^ AUC/cell from the relative F4/FuR plots for the durations indicated by the color-coded bars in G (*n*=401 cells from the same three experiments analyzed in F). (J) Changes to cytosolic Ca^2+^ (relative F4/FuR ratio) in cells before and after thrombin (1 U/ml) and the TRPV4 agonist GSK1016790A (5 µM) addition. Plots from individual cells (*n*=24) from one experiment are presented. (K) Quantification of Ca^2+^ AUC/cell from the relative F4/FuR plots for the durations indicated by the color-coded bars in J (*n*=106 cells from three experiments). (L) Quantification of blebbing in MDA-MB-231 cell populations before and after sequential addition of thrombin and GSK1016790A. Bars represent the mean±s.d. from the same three experiments analyzed in K. The *P*-values were determined by using one-way ANOVA with Tukey's multiple comparisons in F and L, and the Friedman test with Dunn's multiple comparisons in I and K.

The contact-induced Ca^2+^ response associated with blebbing attenuation (Figs 2 and 3) suggested the activation of a mechanosensitive Ca^2+^ channel. We hypothesized that this channel could be Piezo1 as previous findings have established that Piezo1 is expressed and active in MDA-MB-231 cells (Weng et al., 2018). Confocal microscopy imaging of an MDA-MB-231 cell immunostained for Piezo1, and counterstained with wheat germ agglutinin (WGA) to visualize the plasma membrane, revealed Piezo1 positive puncta throughout the cell body (Fig. 4A, Max. *z*-projection). Piezo1 was similarly distributed throughout a single *z*-plane collected from the most basal WGA-positive extremity of the cell (Fig. 4A, single basal *z*-plane), and appeared to be enriched around the periphery of this layer (Fig. 4A, insert 1). Total internal reflection fluorescence (TIRF) microscopy of Piezo1 distribution also detected Piezo1-positive puncta; however, discrete linear aggregates of Piezo1 were also found associated with cortical F-actin filaments (Fig. 4B–D). This pattern of Piezo1 distribution and association with F-actin aligns with the recent model proposed by Ellefsen et al., where Piezo1 channels in close proximity to focal adhesions are locally activated by actomyosin-mediated contractions (Ellefsen et al., 2019). Piezo1-positive puncta were similarly observed within the blebs of thrombin-stimulated cells, and although most of this signal was from the cytosolic fraction of the bleb, membrane-associated Piezo1 was also evident (Fig. S3C). Functional studies of Piezo1 have been greatly facilitated by use of the established chemical Yoda1, a Piezo1 agonist (Syeda et al., 2015; Botello-Smith et al., 2019). Yoda1 does not activate Piezo2 (Syeda et al., 2015), and as expected, qPCR analyses confirmed that Piezo2 transcript levels were several orders of magnitude lower than Piezo1 in MDA-MB-231 cells (Fig. S3D). Yoda1 induced a rapid increase in cytosolic Ca^2+^, which could be suppressed by the stretch-activated ion channel inhibitor GsMTx4, indicating the presence of functional Piezo1 channels in these cells (Fig. S3E–H). Yoda1 was similarly found to induce a sharp increase in cytosolic Ca^2+^ in thrombin-treated and blebbing MDA-MB-231 cells (Fig. 4E) and was associated with near complete attenuation of blebbing (Fig. 4F; Movie 4). Note, as Yoda1 is dissolved in DMSO, this solvent was also included here as a control treatment. The Yoda1-induced increase in cytosolic Ca^2+^ was distinct from the transient response observed for thrombin, and although it declined over time, it typically did not return to baseline for the duration of imaging experiments (Fig. 4G–I). These data confirm that Piezo1 activation has the capacity to attenuate blebbing in MDA-MB-231 cells.

To assess whether the attenuation of thrombin-induced blebbing was specifically mediated by Piezo1 or could be evoked by activation of other mechanosensitive ion channels, we assessed the capacity of the TRPV4 agonist GSK1016790A to attenuate thrombin-induced blebbing. GSK1016790A only elevated Ca^2+^ levels in a subset of thrombin-stimulated cells (Fig. 4J,K), but failed to attenuate thrombin-induced blebbing (Fig. 4L). Similarly, only a subset of thrombin-stimulated cells elevated their cytosolic Ca^2+^ in response to naltriben, an activator of TRPM7 (Hofmann et al., 2014), and it did not appear to attenuate thrombin-induced blebbing (Fig. S4A–C). The cytosolic Ca^2+^ response to GSK1016790A and naltriben was also imaged in the absence of thrombin, and in responsive cells, both agonists induced predominantly transient profiles of Ca^2+^ elevation. However, a substantial subset of cells proved to be largely insensitive to these agonists (Fig. S4D,E). Subsequent addition of Yoda1 to GSK1016790A-treated cells revealed a rapid and sustained cytosolic Ca^2+^ response in all cells (Fig. S4D). Similarly, cells that were insensitive to naltriben responded to Yoda1; however, the Yoda1-induced Ca^2+^ response profiles were altered such that the presence of naltriben appeared to delay the Yoda1 response in some cells (Fig. S4E). These results indicate that Piezo1 is readily activated in the majority of MDA-MB-231 cells, whereas only a subset of cells proved sensitive to TRPV4 and TRPM7 agonists, and support Piezo1 as the main candidate responsible for the bleb-attenuating effects induced by contact.

### Piezo1 depletion reduces the capacity of Yoda1 to attenuate thrombin-induced blebbing

**Fig. 5.**
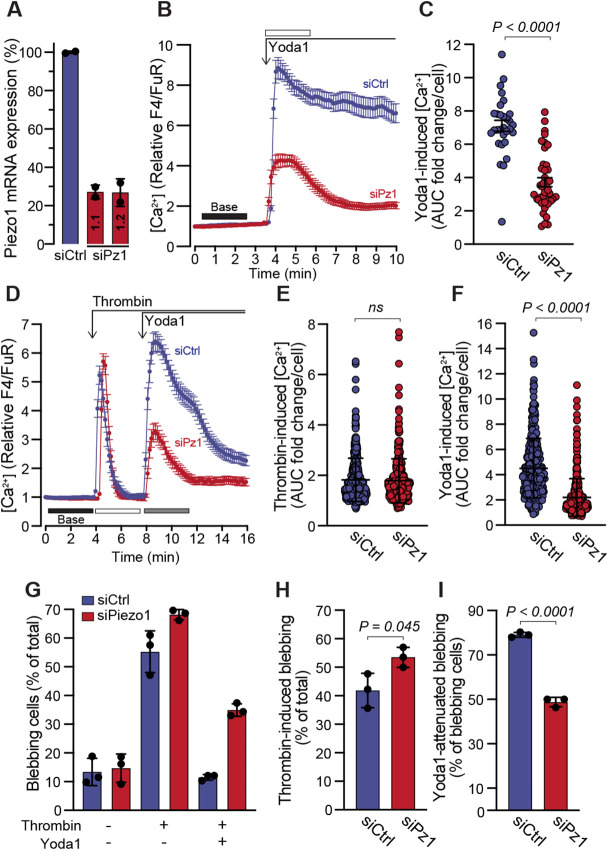
**Piezo1 depletion impairs the capacity of Yoda1 to attenuate blebbing.** (A) Relative expression of Piezo1 mRNA in cells transfected with control siRNA (siCtrl) or two siRNAs targeting Piezo1 (siPz1 1.1 and 1.2). Bars represent the mean±s.d. from two transfections. (B) Yoda1 (20 µM) induced changes to cytosolic Ca^2+^ in siCtrl or siPz1 transfected cells. The plot represents the mean±s.e.m. over time for *n*=30 siCtrl and *n*=40 siPz1 cells. (C) Quantification of Yoda1-induced Ca^2+^ AUC fold change/cell from the relative F4/FuR plots in B for the durations indicated by the color-coded bars. (D) Effects of sequential treatments with thrombin (1 U/ml) and Yoda1 (20 µM) on cytosolic Ca^2+^ in siCtrl and siPiezo1 transfected cells. The plot represents the mean±s.e.m. (*n*=130 for siCtrl and *n*=95 for siPz1 transfected cells, each from one experiment). (E,F) Quantification of thrombin- and Yoda1-induced Ca^2+^ increase from the relative F4/FuR plots for the durations indicated by the color-coded bars in D (*n*=365 siCtrl cells and *n*=370 siPz1 cells from three experiments). (G) Quantification of blebbing in siCtrl- and siPz1-transfected cells from the experiments in D–F. (H) Quantification of blebbing induced by thrombin in siCtrl and siPz1. (I) Quantification of blebbing attenuation in siCtrl and siPz1 cells in response to Yoda1. In G–I, bars represent the mean±s.d. The *P*-values were determined by using the Mann–Whitney test in C, E and F, and unpaired two-tailed Student's *t-*test in H and I.

Next, we assessed the effects of Piezo1 depletion on thrombin-induced blebbing as well as Yoda1 attenuation of blebbing. Cells were transfected with two siRNAs against Piezo1, and RT-qPCR confirmed that Piezo1 transcript expression was significantly reduced, as was the Ca^2+^ response to Yoda1 (Fig. 5A–C). Cells treated with siCtrl or a mix of both Piezo1 siRNAs were exposed to thrombin followed by treatment with Yoda1. The thrombin-induced Ca^2+^ response was not affected by Piezo1 suppression, however, as before, the siPiezo1-treated cells exhibited a significantly lower Ca^2+^ response to Yoda1 (Fig. 5D–F). Quantitative assessment of blebbing status revealed that thrombin-induced blebbing was greater in siPiezo1-treated cells as compared with control cells, and that the capacity of Yoda1 to attenuate blebbing was significantly reduced in siPiezo1-treated cells (Fig. 5G–I). We also aimed to assess the effect of Piezo1 depletion on contact-induced bleb attenuation. However, although siPiezo1 treatment consistently impaired the Yoda1-induced Ca^2+^ response (Fig. S4F), we did not observe a reduction in the Ca^2+^ response to contact compression (Fig. S4G). Given that contact compression will likely activate many different mechanosensitive channels, it is possible that through compensatory effects the net Ca^2+^ influx is similar between siCtrl and siPiezo1. Nonetheless, as we could not assess the effects of compression in Piezo1-depleted cells, we have yet to determine whether Piezo1 activation alone is sufficient to mediate the bleb attenuation observed in response to contact.

### Piezo1 activation inhibits thrombin-induced blebbing and ERM phosphorylation

**Fig. 6.**
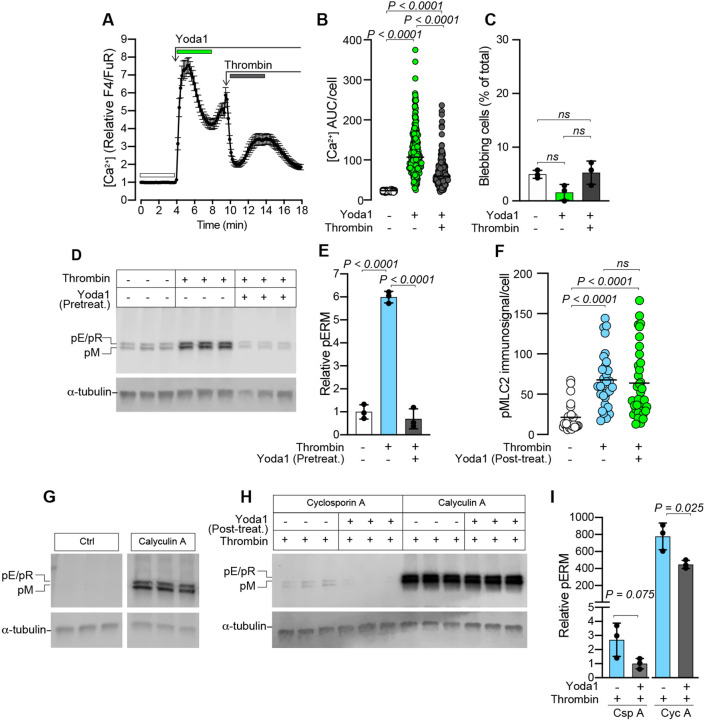
**Pretreatment with Yoda1 inhibits thrombin-induced blebbing and ERM phosphorylation.** (A) Yoda1- (20 µM) and thrombin- (1 U/ml) induced changes to cytosolic Ca^2+^ in MDA-MB-231 cells. The plot represents the mean±s.e.m. over time for *n*=70 cells from one experiment. (B) Quantification of Ca^2+^ AUC/cell for the durations indicated by the color-coded bars in A (*n*=258 cells from three experiments). *P-*values were determined by the Friedman test with Dunn's multiple comparisons. (C) Quantification of blebbing in cell populations treated sequentially with Yoda1 and then thrombin as indicated; bars represent the mean±s.d. for the same three experiments analyzed in B. *P-*values were determined by one-way ANOVA with Tukey's multiple comparisons. (D) Immunoblotting for pERMs in cells pre-treated for 15 min with or without Yoda1 (20 µM), followed by thrombin (1 U/ml) exposure for 5 min. (E) Quantification of pERM band intensities relative to α-tubulin loading controls for the samples in panel D. Bars represent the mean±s.d. for three samples. (F) Quantitative image analysis of pMLC2 immunofluorescence/cell in untreated control cells, or cells treated with thrombin (5 min), followed by 10 min incubation with or without Yoda1. Plots represent average pMLC2 intensity for *n*=26, 30 and 35 cells, respectively from one experiment. *P*-values were determined by Kruskal–Wallis test with Dunn's multiple comparisons. (G) Immunoblotting for phosphorylated ERMs in cells incubated with or without Calyculin A (50 nM); the Ctrl and Calyculin A panels in G were cropped from the same immunoblotted membrane. (H) Immunoblotting for pERMs in cells first stimulated with thrombin (1 U/ml) followed by 5 min treatment with Calyculin A (50 nM) or Cyclosporin A (250 nM), and subsequently (5 min later) treated with or without Yoda1 (20 µM) for 15 min. α-tubulin was used as a loading control in D, G and H. (I) Quantification of pERM band intensities relative to α-tubulin loading controls for the samples in F. Bars represent the mean±s.d. The *P*-values were determined by unpaired two-tailed Student's *t-*test. For the immunoblots in D, G and H, the sample lanes for each condition represent biological replicates from the same experiment.

MDA-MB-231 cells were pre-treated with Yoda1 followed by thrombin treatment to test whether Piezo1 activation could desensitize cells to thrombin-induced blebbing. Cytosolic Ca^2+^ was, as before, elevated in response to Yoda1, whereas the thrombin-induced Ca^2+^ response was less pronounced following pre-treatment with Yoda1 (Fig. 6A,B). Indeed, thrombin did not induce blebbing when cells were pre-treated with Yoda1 (Fig. 6C). Further, pre-treatment with Yoda1 effectively prevented thrombin-induced ERM phosphorylation (Fig. 6D,E), and treatment with Yoda1 after stimulation with thrombin was also shown to reduce ERM phosphorylation (Fig. S5A,B,E). Taken together, these data support a role for Piezo1 activation in counteracting ERM phosphorylation. As we had observed increased levels of pMLC2 in thrombin-treated cells (Fig. 1N), we next analyzed immunostained cells to determine the effect of Yoda1 on pMLC2 levels after thrombin treatment. pMLC2 levels were significantly increased in thrombin-treated cells, but Yoda1 did not significantly reduce the average pMLC2 levels in thrombin-stimulated cells (Fig. 6F).

We next attempted to identify phosphatases with the capacity to dephosphorylate ERM proteins. In the presence of the calcineurin (PP3 phosphatase) inhibitor cyclosporin A (Csp A), thrombin still induced ERM phosphorylation that could be reduced by Yoda1 (Fig. 6H,I), to a similar degree as observed in the absence of Csp A (Fig. S5A,B), suggesting that calcineurin is not involved in regulating pERM levels in MDA-MB-231 cells. The PP1/PP2A family of serine/threonine phosphatases are inhibited by calyculin A (Cyc A) (Brown et al., 2003), and Cyc A treatment alone was sufficient to induce a marked increase in pERM levels compared with controls (Fig. 6G), suggesting that the PP1/PP2A phosphatases participate in the constitutive dephosphorylation of ERMs. Similarly, in cells treated with Cyc A and then exposed to thrombin, the relative levels of ERM phosphorylation (Fig. 6H,I) were higher than those seen with thrombin alone (Fig. 6D,E), and although Yoda1 reduced pERM levels (Fig. 6H,I), they still exceeded those induced by thrombin alone. Furthermore, Cyc A alone had no effect on cytosolic Ca^2+^, but did induce blebbing and prevented Yoda1-mediated attenuation of blebbing in the presence of thrombin (Fig. S6). As Yoda1 could partially counteract the effect of CycA-mediated PP1/PP2A inhibition, i.e. by reducing pERM levels, Piezo1 activation might promote downstream PP1/PP2A activity or potentiate the activity of other phosphatases that can partly compensate for the loss of PP1/PP2As.

## DISCUSSION

The plasticity of tumor cells permits them to adopt a range of migratory modes, of which the amoeboid phenotype is in part defined by plasma membrane blebbing, and is implicated in tumor metastasis (Tozluoğlu et al., 2013). The tumor environment is thrombotic (reviewed in Reddel et al., 2019) due to the low integrity of tumor vessels, and thrombin has been proposed to increase the invasiveness of MDA-MB-231 breast cancer cells (Henrikson et al., 1999). Here we identify thrombin as a potent stimulus for blebbing in breast cancer cells and establish that activation of its receptors PAR1 or PAR2 is sufficient to induce this effect. A recent report demonstrates that PAR4 activation similarly induces blebbing (Vanderboor et al., 2020). During controlled compression of cells, we unexpectedly discovered that gentle mechanostimulation – referred to here as contact compression – could attenuate blebbing. This attenuation of blebbing was reproduced using the Piezo1-agonist Yoda1, and we present data to support a mechanism whereby Piezo1 activation leads to silencing of blebbing with an associated reduction in ERM phosphorylation, and propose that PP1/PP2A phosphatases might be required for this effect.

The initial expansion of a bleb requires dissociation of the PM from the underlying actin cortex. This actin-free bleb balloons outwards, after which the contractile machinery required for bleb retraction is sequentially recruited; firstly, the ERMs crosslinking actin and the PM, followed by actin and finally myosin II (Charras et al., 2006). Increased actomyosin contraction is sufficient to trigger bleb expansion, as it increases hydrostatic pressure within the cell and destabilizes areas of weak actin–PM contact (Tinevez et al., 2009), and targeted dephosphorylation of ERMs also promotes actin–PM dissociation and bleb formation (Welf et al., 2020). Consequently, cells deficient in actin–PM crosslinkers are more likely to bleb, and mechanistic studies of blebbing have been facilitated by the M2 melanoma cell line, which spontaneously bleb in part due to a deficiency in filamin (Charras et al., 2008; Cunningham et al., 1992). The spontaneous blebbing observed here in MDA-MB-231 cells (Fig. S1) is likely in part explained by a deficiency in the actin–PM linker and tumor suppressor Merlin (Shapiro et al., 2014; Morrow et al., 2011), which is related to the ERM proteins.

In the present study, agonist activation of PAR1 was sufficient to reproduce the blebbing effects observed for thrombin (Fig. 1), as did PAR2 activation (Fig. S2). Thrombin has been reported to act directly on PAR2, but can also transactivate PAR2 via the PAR1-tethered ligand (Heuberger et al., 2019; Mihara et al., 2016; Lin and Trejo, 2013). The potential relevance of such pathways to breast cancer is supported by the fact that thrombin is present in breast cancer tissues (Fig. S2), and PAR2 expression is elevated in breast tumor specimens and in breast cancer cell lines, including MDA-MB-231 cells (Su et al., 2009). Thrombin activation of PARs leads to phospholipase C (PLC)-mediated hydrolysis of PIP2 to DAG and IP3, which likely increases blebbing through a number of pathways. PIP2 serves as a binding site for ERMs, and therefore, loss of PIP2 reduces the stability of actin–PM contacts (Ben-Aissa et al., 2012; Hao et al., 2009), which might promote bleb initiation. IP3-stimulated Ca^2+^ release from the ER increases actomyosin contraction, which drives expansion of actin-free bleb membranes. The thrombin-induced increase in cytosolic Ca^2+^ occurred prior to bleb initiation; however, although some cells started to bleb in direct response to elevated Ca^2+^, in others the blebbing response was somewhat delayed (Fig. 2B, Fig. 3E, Fig. 4E). This might reflect differences in the balance between the resting levels of actomyosin contractility and actin–PM contacts in individual cells before thrombin stimulation, such that some cells are more susceptible than others to adopting blebbing behavior. DAG in combination with increased cytosolic Ca^2+^ also activates PKC, which can phosphorylate and activate ERMs (Adyshev et al., 2013). ERM phosphorylation at the PM exposes their actin-binding domains (Matsui et al., 1998), and this activation is implicated in coupling the bleb membrane to the actomyosin contractile force that ensures the retraction of blebs (Aoki et al., 2016; Charras et al., 2006). Cells with higher levels of ERM proteins have a reduced risk of bleb formation (Sliogeryte et al., 2014), whereas loss of ERMs is associated with greater blebbing activity (Diz-Muñoz et al., 2010). In contrast, here we observed that increased ERM phosphorylation was associated with thrombin-induced, myosin-dependent blebbing, and these phosphorylated ERMs were clearly enriched in bleb membranes (Fig. 1Q; Fig. S5D). Thrombin similarly induces ERM phosphorylation in endothelial cells (Adyshev et al., 2013), so this effect is not limited to MDA-MB-231 cells. This activation of ERMs might reflect a cellular response to reinstate contacts between the actin cortex and the PM following bleb expansion; however, in cells with elevated actomyosin contractility, it is conceivable that this recoupling might also promote blebbing by connecting the PM to the contracting cytoskeleton, contributing to an increase in hydrostatic pressure. This possibility is partly supported by the fact that in cells treated with the myosin inhibitor para-aminoblebbistatin, thrombin-induced blebbing was impaired, even though ERM phosphorylation was similarly increased in untreated controls (Fig. 1). Yoda1 treatment significantly reduced thrombin-induced ERM phosphorylation, which was associated with attenuated blebbing (Fig. 6), possibly by reducing the availability of active ERM contact sites between the PM and the contracting actomyosin network.

Cellular confinement or compression has been implicated as a driving force behind cells adopting bleb driven modes of migration (Liu et al., 2015; Logue et al., 2015; Ruprecht et al., 2015; Wisniewski et al., 2020; Zhao et al., 2019), and MDA-MB-231 cells favor this mode through narrow (3 µm wide) microchannels (Holle et al., 2019). The mechanosensitive ion channels TRPM7 and TRPV4 have been identified as important confinement sensors (Nam et al., 2019; Zhao et al., 2019, 2021); however, in this study, agonists of these channels did not replicate the bleb-attenuating effects of Yoda1, suggesting that the confinement-mediated attenuation of blebbing observed here might be selective to Piezo1 activation. Piezo1 has previously been implicated as a confinement sensor, which suppresses protein kinase A (PKA) activity and optimizes cell motility in response to the mechanical properties of the environment (Hung et al., 2016). Similarly, changes in actomyosin contractility and substrate adhesion can induce cells to transition between bleb- and lamellipodia-dependent cell locomotion modes (Bergert et al., 2012). Recently, Srivastava et al. identified Piezo in *Dictyostelium* cells as a pressure sensor, required for a Ca^2+^-dependent switch from pseudopod to bleb-based migration (Srivastava et al., 2020), and Piezo1 depletion also impaired endothelial cell migration in a transwell assay (Zhang et al., 2017). However, melanoma cell chemotaxis through a 3D collagen matrix or transmigration through synthetic pores is unaffected by GsMTx4-mediated inhibition of stretch-activated channels such as Piezo1 (Lomakin et al., 2020). In contrast, Yoda1 impedes cell migration in transformed fibroblasts (Chubinskiy-Nadezhdin et al., 2019), and here, we found that contact compression and Yoda1-activation of Piezo1 reduced both spontaneous and thrombin-induced blebbing in MDA-MB-231 breast cancer cells, which is likely to limit their capacity to engage in amoeboid migration. These results collectively indicate a role for Piezo1 as a modifier of migratory behavior, but the effect may differ between cell types and by the specific context in which the channel is activated.

A mechanosensing role for the nucleus in inducing blebbing and amoeboid behavior has recently been elucidated, whereby actomyosin contractility is increased through the action of cytosolic phospholipase A2 (cPLA2), which is activated by Ca^2+^ release from internal stores (Lomakin et al., 2020; Venturini et al., 2020). We similarly observed that compressing cells beyond the point of initial contact with the cell press pillar resulted in elevated cytosolic Ca^2+^, which was associated with an increase in projected nuclear area and the formation of blebs (Fig. 2). In contrast, gentle contact compression, which also induced a Ca^2+^ response, attenuated both spontaneous and thrombin-induced blebbing (Figs 3, 4), as did Piezo1 activation with Yoda1 (Fig. 4). This suggests that the contact compression studied here activates pathways that precede those activated by the greater compression exerted during deformation. In this regard, changes in PM tension impact the activity of the kinase TORC2 in a PIP2-dependent manner (Berchtold et al., 2012; Riggi et al., 2018), and it remains to be elucidated if the contact compression studied here might similarly alter the activity of potential blebbing regulators independently of Piezo1, and how PIP2 hydrolysis by thrombin might affect such a mechanism.

The activation threshold of Piezo1 has previously been shown to be lower in cytoskeleton-free PM blebs due to the loss of mechanoresistance that cortical actin provides against changes in membrane tension (Cox et al., 2016). Importantly, it has also been established that forces transmitted through the actin cytoskeleton (Gottlieb et al., 2012) and generated by the actomyosin network can activate Piezo1 (Ellefsen et al., 2019; Pathak et al., 2014). Given that thrombin increases actomyosin contraction, which contributes to the expansion of initially actin-free blebs, it is conceivable that Piezo1 found in the bleb membrane (Fig. S3) would have heightened sensitivity to agonists and mechanostimuli. Furthermore, although Piezo1 depletion impaired the ability of Yoda1 to attenuate blebbing, Piezo1-depleted cells were also slightly more susceptible to thrombin-induced blebbing (Fig. 5). This suggested that Piezo1 activity might also contribute to counteracting bleb initiation in these cells, and this possibility was supported by the fact that thrombin failed to induce blebbing in cells that were pre-treated with Yoda1 (Fig. 6). Piezo1 is also regulated by the phosphoinositide composition of the PM (Borbiro et al., 2015) and through interactions with SERCA2 in the ER (Zhang et al., 2017), and given that ER–PM contact sites are disrupted by bleb expansion (Hsieh et al., 2017) further studies are warranted to assess how Piezo1 regulation might be affected by blebbing.

Notably, here we report elevated cytosolic Ca^2+^ associated with thrombin-mediated bleb induction, and also Piezo1-activated bleb attenuation. These apparently opposing effects of elevated Ca^2+^ are likely explained by multiple factors, including possible spatial differences such that Ca^2+^ entering through Piezo1 at the PM would initially supply a high Ca^2+^ concentration at potential actin–PM contact sites, whereas thrombin stimulates release of Ca^2+^ from internal ER stores. There are also noticeable differences in the response profiles such that thrombin induces a transient relatively short-lived Ca^2+^ elevation, whereas Yoda1 and contact compression typically induced a more sustained Ca^2+^ response. Previous work has established how differences in the amplitude and duration of Ca^2+^ signaling can be deciphered to selectively activate specific transcription factors (Timmerman et al., 1996; Dolmetsch et al., 1997), and similar differentiation mechanisms could be relevant to our findings. Related to these differences, we also determined that prolonged exposure to Yoda1, but not thrombin, increased caspase-3/7 activation (Fig. S3), likely a consequence of the sustained elevation of cytosolic Ca^2+^ (reviewed in Rizzuto et al., 2003). Yoda1 sensitizes cancer cells including MDA-MB-231 cells to tumor necrosis factor-related apoptosis-inducing ligand (TRAIL)-mediated apoptosis (Hope et al., 2019), but in other cell types, Yoda1 alone does not reduce cell viability (Blythe et al., 2019; Chubinskiy-Nadezhdin et al., 2019; Davies et al., 2019). This highlights a limitation of our study in which we have focused on the MDA-MB-231 breast cancer cell line, and future work will be required to confirm to what degree our findings are applicable to other breast cancer as well as non-neoplastic cell types. We also observed some variability regarding the proportion of cells that initiate blebbing in response to thrombin, and although we have not established a definitive explanation for this, the relative effects were consistent throughout this study.

PP1/PP2A phosphatases are responsible for the dephosphorylation of ERMs in T-cells (Brown et al., 2003), and here, PP1/PP2A inhibition with Cyc A resulted in increased pERM levels and impaired Yoda1-mediated reduction in pERM levels (Fig. 6). Amongst the PP1/PP2A phosphatases the B″/PR72 regulatory subunit is reported to be regulated by Ca^2+^ (Wlodarchak et al., 2013; Janssens et al., 2003; Ahn et al., 2007). It is conceivable that Ca^2+^ influx via Piezo1 might promote PP1/PP2A activity. This would promote dephosphorylation of pERMs and thus reduce the actomyosin contractile forces exerted on the PM, and in doing so attenuate blebbing. However, we cannot exclude the possibility that Piezo1 activation might promote other phosphatases that can compensate for the loss of PP1/PP2A-mediated ERM dephosphorylation. Equally, the Piezo1-mediated influx of Ca^2+^ might indirectly promote ERM dephosphorylation through other signaling pathways, or might promote phosphatase activity in a Ca^2+^-independent manner. Cyc A treatment alone was sufficient to rapidly induce blebbing in MDA-MB-231 cells (Fig. S6), and Cyc A can also impair myosin-light-chain phosphatases (Peterson et al., 2004). Therefore, the observed effects are likely not limited to ERM phosphorylation status alone, but also potentiation of myosin activity. In contrast, Yoda1 stimulation of thrombin-treated cells did not have a significant effect on myosin activity (Fig. 6F), suggesting that its bleb-attenuating effects are limited more to reducing the activation state of ERMs. PP2As are important tumor suppressors; however, in breast tumors and in breast cancer cell lines, including MDA-MB-231 cells, endogenous PP2A inhibitors are overexpressed (Janghorban et al., 2014). In light of our findings, downregulation of PP2As in MDA-MB-231 cells might also contribute to their susceptibility to blebbing, including thrombin-induced blebbing, whereas Piezo1 activation promotes ERM dephosphorylation and attenuates blebbing.

## MATERIALS AND METHODS

### Reagents and chemicals

Fluo-4 and Fura Red Ca^2+^ indicators (ThermoFisher Scientific, Uppsala, Sweden) were resuspended in DMSO to 1 mM prior to dilution and cell staining. Wheat germ agglutinin (WGA), conjugated to Alexa Fluor 488 or 633 (ThermoFisher Scientific) was used to counterstain the plasma membrane and stored at −20°C. NucBlue live nuclear stain (Molecular Probes, ThermoFisher Scientific) was stored at 4°C. Human alpha thrombin (Nordic Diagnostica AB, Billdal, Sweden, and Nordic Biosite, Täby, Sweden) was resuspended in OptiMEM reduced serum medium (RSM) (Thermo Fisher Scientific) to 1 U/µl and stored at −20°C. SiR-Actin (Spirochrome, Tebu-bio, Roskilde, Denmark) was diluted to 1 mM in dimethyl sulfoxide (DMSO; ThermoFisher Scientific, Uppsala, Sweden) and stored at −20°C. The PAR1 agonist peptide TFLLR-NH2 (Abcam, Cambridge, UK) was diluted to 1 mM in OptiMEM RSM and stored at −20°C. The PAR2 agonist peptide SLIGRL-NH2 (Abcam, Cambridge, UK) was diluted to 1 mM in OptiMEM RSM and stored at −20°C. Para-aminoblebbistatin (Axol Bioscience Ltd, Cambridge, UK), the myosin II inhibitor, was diluted to 20 mM in DMSO and stored at −20°C. Yoda1 (Tocris, Bio-Techne, Abingdon, UK), the Piezo1 channel activator, was diluted to 20 mM in DMSO and stored at −20°C. GsMTx-4, the mechanosensitive and stretch-activated ion channel inhibitor (Abcam, Cambridge, UK), was diluted to 250 µM in DMSO and stored at −20°C. The TRPV4 agonist GSK1016790A (Tocris) was diluted to 50 mM in DMSO and stored at −20°C. The TRPM7 activator naltriben methanesulfonate hydrate (Naltriben; Merck, Solna, Sweden), was diluted to 20 mM in DMSO and stored at −20°C. Calyculin A (Tocris) was diluted to 1 mM and Cyclosporin A (Tocris) was diluted to 100 mM in DMSO and stored at −20°C. Polydimethylsiloxane (PDMS) pillars [Sylgard 184 silicone elastomer using a 10 (base):1 (curing agent) ratio (Sigma-Aldrich Sweden AB, Stockholm, Sweden)] were cast from a custom mold produced with a Form2 3D printer (Formlabs). Staurosporine (DMSO solution), protein kinase inhibitor (ab146588; Abcam, Amsterdam, Netherlands) was stored at −20°C.

### Antibodies

Piezo1 rabbit pAb (NBP1-78446; Novus Biologicals, Bio-Techne, Abingdon, UK) was selected as its specificity has previously been demonstrated (Gudipaty et al., 2017). Phospho-myosin light chain 2 polyclonal antibody (PA5-67499; ThermoFisher Scientific) was used for immunostaining fixed cells. Phospho-ezrin (Thr567)/radixin (Thr564)/moesin (Thr558) mouse mAb (48G2; Cell Signaling Technology) and α-tubulin rabbit mAb (11H19; Cell Signaling Technology) were detected with Alexa Fluor goat anti-rabbit IgG secondary antibodies conjugated to 680 nm fluorophores (A32734, ThermoFisher Scientific), for phospho-ERM and α-tubulin immunoblotting. For immunocytochemistry antibodies were detected with Alexa Fluor Plus goat anti-rabbit IgG secondary antibodies conjugated to 488 nm (A32731, ThermoFisher Scientific) or 555 nm fluorophores (A32732, ThermoFisher Scientific).

### Cell culture

The MDA-MB-231 breast cancer cell line was obtained from American Type Culture Collection (ATCC) (LGC Standards GmbH, Wesel, Germany) and externally authenticated using Idexx Bioanalytics (Ludwigsburg, Germany), which confirmed their genetic signature to be consistent with that published for the cell line of origin (Yu et al., 2015), and that they were free of contaminants. Cells were cultured in Dulbecco's modified Eagle's medium (DMEM) Glutamax (ThermoFisher Scientific) supplemented with 10% fetal bovine serum (FBS) (ThermoFisher Scientific) under standard conditions of 37°C and 5% CO_2_.

### Ca^2+^ imaging

For all live-imaging experiments, cells were seeded in MatTek (MatTek Corporation, Bratislava, Slovak Republic) or Ibidi (LRI Instrument Ab, Lund, Sweden) 35 mm dishes with uncoated coverslip bottoms. For Ca^2+^ imaging, cells were incubated with 1 µM Fluo-4 and Fura Red in OptiMEM reduced serum medium without Phenol Red (referred to hereafter as OptiMEM) for 1 h, after which cells were washed and cultured for an additional 1.5 h in OptiMEM before imaging and treatments. Time-lapse imaging of Fluo-4 and Fura Red fluorescence, and differential interference contrast (DIC) channels was performed on an LSM700 confocal microscope (Zeiss, Jena, Germany) equipped with an incubation chamber, using a Plan-Apochromat 20×/0.8 (Zeiss), or a Plan-Apochromat 63×/1.4 (Zeiss) objective with a pinhole setting of 1 Airy unit. For Ca^2+^ imaging, Fluo-4 fluorescence and DIC images were scanned simultaneously on track 1, followed by Fura Red fluorescence scanning on track 2. Prior to the indicated treatments, a baseline for Fluo-4 and Fura Red fluorescence was established. Treatments were administered manually by pipetting reagents directly into the culture dish. To facilitate access to the culture dish when administering treatment solutions, the head of the microscope was tilted backwards, which temporarily obstructed DIC imaging. The stated treatment concentrations account for the dilution of reagents in the imaging medium.

### F-actin imaging

Cells were seeded in MatTek (MatTek Corporation, Bratislava, Slovak Republic) or Ibidi (LRI Instrument Ab, Lund, Sweden) 35 mm dishes with uncoated coverslip bottoms. Prior to live imaging of F-actin, cells were incubated overnight with 0.5 µM SiR-actin in DMEM Glutamax, 10% FBS. Confocal fluorescence and differential interference contrast (DIC) time-lapse imaging of SiR-actin labeled cells was performed on an LSM700 confocal microscope (Zeiss, Jena, Germany), as described for Ca^2+^ imaging above.

### Image analysis and processing of time-lapse sequences

Time-lapse sequences of Fluo-4 and Fura Red fluorescence and DIC recordings were analyzed using the Fiji version of ImageJ (Schindelin et al., 2012). Individual cells in the DIC recordings were viewed for the duration of the time-lapse experiment and the total area occupied by each cell was outlined with the polygon tool and annotated as a region of interest (ROI). The Fluo-4 and Fura Red fluorescence intensity was measured for each ROI using the multi-measure function in ImageJ and the values were exported to Excel where the Fluo-4/Fura Red ratio, the relative Fluo-4/Fura Red signals, and the area under the curve (AUC) of the Fluo-4/Fura Red responses were calculated for the durations of treatments indicated in the respective figures. In time-lapse experiments where focal drift was observed to alter the out-of-cell Fluo-4/Fura Red background fluorescence, corrections were applied to all ROIs. The blebbing status for each cell during the indicated treatments was visually assessed and manually recorded in Excel. Blebbing behavior in MDA-MB-231 cells could be broadly subdivided into cells that expanded and retracted large well-defined blebs around their perimeter and cells that produced smaller blebs that were either found around their perimeter or concentrated to one part of the cell. Both these states were annotated as blebbing. Cells were considered to have stopped blebbing when no new bleb protrusions were seen to form, but some cells occasionally still had unretracting protrusions on their surface. Where it was difficult to visually discern the boundaries for entangled clusters of 3–4 cells, the area occupied by the cluster was annotated as a single ROI for the purpose of Fluo-4 and Fura Red fluorescence analysis, but where possible, the blebbing status for each cell in the cluster was noted. Therefore, the total number of cells analyzed for Ca^2+^ signaling, which is noted in the figure legends, is not always exactly the same as the number of cells analyzed for blebbing. Kymographs of DIC and Fluo-4/Fura Red recordings were prepared by applying the ImageJ reslice command to linear ROIs plotted through individual cells. To prepare the ratiometric Fluo-4/Fura Red kymographs using the ImageJ Image Calculator tool the boundary defined by the Fluo-4 and Fura Red signals in a composite image of the resliced cell was assigned as a single ROI. The ImageJ Clear Outside function was used to clear fluorescence from all pixels outside of this ROI (i.e. in the cell-free background). The Fluo-4 signal was then divided by the Fura Red signal and the ratiometric fluorescence was presented; no quantitative analysis was performed on images processed like this.

### Cell compression

Cell compression experiments were conducted with the ‘cell press’, a custom-built manipulator composed of 3D printed part, a linear piezoelectric positioner (SLC-1730, SmarAct GmbH, Oldenburg, Germany) operated with an MCS2 manual controller (SmarAct), and a flexible PDMS pillar cast from a 3D-printed mold. The cell press was fixed to the microscope behind the translation stage (Fig. 2A). To control the position of the PDMS pillar relative to the cell layer, we established and recorded the *z*-position displayed on the MCS2 controller for the point-of-contact between the surface of the PDMS pillar and the bottom of a cell-free dish. To do so, ink markings were made on the surface of the pillar and the surface of the coverslip. The piezoelectric positioner was moved to its start position ensuring the pillar was at its maximum distance from the coverslip. While visualizing the ink marking on the coverslip under the microscope, the pillar was lowered until its ink marking was also visible. The positioner was then set to descend in 5 µm steps. We observed that, due to its flexibility, once the PDMS pillar was in contact with the coverslip it moved in sync with small *x* or *y* positional changes to the coverslip. Therefore, for each step that the pillar was lowered, we tested if it had contacted the coverslip by making small adjustments to the translation table until both ink markings moved in sync, indicating that the pillar was in contact with the coverslip. This position was recorded and used as a reference point for subsequent cell compression experiments. Fluo-4- and Fura Red-labeled cells were visualized as before and the pillar was then lowered until it was within approximately 100 µm of the recorded reference point, permitting a baseline of non-compressed signal to be recorded. The positioner was then lowered in 5 µm steps until a Ca^2+^ response was observed, this was considered the point of contact compression. For deformation compression the pillar was further lowered (typically in 2 µm steps) until the cells were visibly deformed.

To study the effects of compression on nuclear deformation, cells were labeled with NucBlue live nuclear stain. The projected nuclear area was calculated by selecting individual nuclei as ROIs in ImageJ, thresholding for the NucBlue signal, and converting the timelapse to a sequence of binary images. The particle analysis tool was then used to determine the projected nuclear area for each timepoint in the image sequence. The average projected nuclear area was calculated for the different specified states of compression. Due to the hydrophobic properties of PDMS, we observed no binding or attachment of cells to the surface of the pillar during the compression experiments.

To assess the force exerted on cells by the PDMS pillar during different compression states, the holder for the PDMS pillar was redesigned to house a Futek LTH300 donut load cell (Microepsilon, Sensotest AB, Järfälla, Sweden), into which a custom-fitted PDMS pillar was inserted. The load cell was connected to a Futek USB220 high-resolution load cell digital amplifier (Microepsilon), and load data was collected using the Futek Sensit test and measurement software (Microepsilon).

### Piezo1 knockdown

MDA-MB-231 cells were co-transfected with Silencer Select pre-designed siRNAs si18891 and si18892, which respectively target the sequences GCCTCGTGGTCTACAAGATT and AGAAGAAGATCGTCAAGTA of human Piezo1 mRNA. siRNAs (100 pmoles of siPiezo1 or siCtrl) were delivered using Lipofectamine RNAiMax transfection solution (ThermoFisher Scientific). Experiments were performed 48 h post-transfection.

### Reverse transcription and real-time qPCR

RNA was isolated from siPiezo1- or siCtrl-transfected MDA-MB-231 cells using a PureLink RNA Mini Kit (Thermo Fisher Scientific); concentrations were determined by NanoDrop (Thermo Fisher Scientific). cDNA was synthesized by reverse transcription using the iScript kit (BioRad, Stockholm, Sweden). Transcripts of interest were analyzed using the QuantStudio 5 real-time quantitative PCR (RT-qPCR) system with the associated software (Thermo Fisher Scientific). cDNA was diluted with the Fast SYBR green master mix (Thermo Fisher Scientific) and Piezo1 mRNA levels were assessed by RT-qPCR using the following primer pairs: forward, 5′-CACCAACCTCATCAGCGACT-3′, and reverse, 5′-GCACCAGCCAGAACAGGTAT-3′; and forward, 5′-AGGGAGGCACTGTGGAGTAT-3′, and reverse, 5′-AGGGATGACCACAGACTGGT-3′. Piezo2 was detected with the following primer pairs: forward, 5′-CACCATCTACAGACTGGCCCAC-3′, and reverse, 5′-ACCAGGTGCCATTTGTTCCTT-3′; and forward, 5′-GACGGACACAACTTTGAGCCTG-3′, and reverse, 5′-CTGGCTTTGTTGGGCACTCATTG-3′. The threshold cycles for Piezo1 and Piezo2 transcripts were normalized to three reference genes using the following primers: Tyrosine 3-monooxygenase/tryptophan 5-monooxygenase activation protein zeta polypeptide (YWHAZ), forward, 5′-CCGTTACTTGGCTGAGGTTG-3′, and reverse, 5′-TGCTTGTTGTGACTGATCGAC-3′; Tata binding protein (TBP), forward, 5′-TTGGGTTTTCCAGCTAAGTTCT-3′, and reverse, 5′-CCAGGAAATAACTCTGGCTCA-3′; Glyceraldehyde 3-phosphate dehydrogenase (GAPDH), forward, 5′-AGCCACATCGCTCAGACAC-3′, and reverse, 5′-GCCCAATACGACCAAATCC-3′. YWHAZ and TBP are reported to be stable reference genes for qPCR analysis of MDA-MB-231-derived transcripts (Lemma et al., 2016).

### Immunostaining and imaging with confocal and total internal reflection fluorescence microscopy

MDA-MB-231 cells were seeded in Nunc Lab-Tek chambers with coverglass bottoms (Thermo Fisher Scientific) and cultured in DMEM Glutamax, 10% FBS. The cell medium was aspirated and the cells were fixed for 10 min (37°C) with 3.7% formalin, DMEM Glutamax, 10% FBS. The fixative was aspirated and the cell layer was washed extensively with Tris-buffered saline (TBS) with 0.1% Tween 20 (TBST). Cells were incubated overnight (4°C) with the specified primary antibodies diluted 1:250 in TBST. After extensive washing, primary antibodies were detected with secondary Alexa Fluor Plus antibodies diluted 1:1000. Cells were counterstained with phalloidin to visualize F-actin, or WGA to visualize the plasma membrane and were additionally stained with NucBlue to label nuclei. Confocal microscopy of immunofluorescence staining and counterstaining was performed as above on an LSM700 confocal microscope (Zeiss) using a Plan-Apochromat 63×/1.4 (Zeiss) objective and images were captured with Zen software (Zeiss). Pin-hole settings were adjusted to render *z*-sections with an optical thickness of approximately 1 µm. TIRF microscopy was performed on a Nikon TiE microscope equipped with an iLAS2 TIRF illuminator for multi-angle patterned illumination (Cairn Research, Faversham, UK) and a 60×/1.49-NA Apo-TIRF objective (Nikon). Excitation light was delivered by 488 nm and 561 nm diode-pumped solid-state lasers with built-in acousto-optical modulators (Coherent, Santa Clara, USA). Fluorescence was detected with a back-illuminated EMCCD camera (DU-897, Andor Technology, Belfast, Northern Ireland) controlled by MetaMorph (Molecular Devices, San Jose, USA). Emission wavelengths were selected with filters (527/27 nm and 590 nm long-pass, Chroma Technology, Rockingham, USA) mounted in a filter wheel (Lambda 10-3, Sutter Instruments, Novato, USA).

### Image analysis of immunostained cells

The intensities of pMLC2 immunosignals associated with MDA-MB-231 cells treated with or without thrombin (1 U/ml) for 5 min, and subsequently incubated for 10 min with or without Yoda1 (20 µM) (Fig. 6F), were analyzed using the Fiji version of ImageJ. OptiMEM was used in control incubations where thrombin or Yoda1 was omitted. The outer boundaries of individual cells were defined by WGA staining and assigned as ROIs. These ROIs were used to analyze the average pMLC2 intensity in each *z*-scan of the acquired *z*-stacks, and these averages were summed to represent the total pMLC2 immunosignal per cell.

### ERM phosphorylation and phosphatase inhibition assay

MDA-MB-231 cells were seeded into 24-well plates (Sarstedt, Newton, NC, USA) and cultured overnight in DMEM Glutamax, 10% FBS (Thermo Fisher Scientific). The culture medium was aspirated and the cells were washed in OptiMEM before being treated as indicated with or without thrombin (1 U/ml) in combination with or without Yoda1 (20 µM) pre-treatment (15 min) or post-treatment (15 min). Inhibition of phosphatases was performed using the PP1/PP2A phosphatase family inhibitor Calyculin A (Cyc A; 50 nM) or the calcineurin inhibitor Cyclosporin A (Csp A; 250 nM). Cells were stimulated with thrombin (1 U/ml) and after 5 min Cyc A or Csp A was added, 5 min later cells were treated with or without Yoda1 (20 µM). All untreated control conditions were exposed to equal concentrations of DMSO as those present in the Yoda1-, Cyc A- and Csp A-treated cultures.

### SDS-PAGE and western blotting

MDA-MB-231 cells were treated as indicated and lysates were prepared using RIPA buffer (Thermo Fisher Scientific, Uppsala, Sweden) supplemented with Complete Protease Inhibitor Cocktail (Roche, Basel, Switzerland) and PhosStop (Roche) or Halt (Thermo Fisher Scientific) phosphatase inhibitor cocktail and diluted in 4× Laemmli buffer (BioRad) containing β-mercaptoethanol. Lysates were denatured and proteins were separated by SDS-PAGE on Mini-Protean 10% tris-glycine gels (BioRad). Precision plus protein Kaleidoscope molecular weight (MW) standards (BioRad) were loaded in one lane of every gel. Separated proteins were transferred to Immobilon-FL polyvinylidene difluoride (PVDF) membranes (Millipore, Cork, Ireland), which were blocked with a 2:1 ratio of Odyssey blocking buffer (LI-COR Biosciences) and 0.1% Tween-20 in TBS (TBST). Membranes were incubated overnight at 4°C with phospho-ERM (pERM) and α-tubulin antibodies diluted 1:1000. After extensive washing, antibodies were detected with secondary goat anti-rabbit Alexa Fluor 680 IgGs (diluted 1:2000) and imaged using an Odyssey Fc system (LI-COR Bioscience). Band intensities were analyzed using Image Studio software (LI-COR Bioscience), and the relative pERM/α-tubulin signal was calculated in Microsoft Excel. Full uncropped images of western blots are shown in Fig. S7.

### Cell death assays

MDA-MB-231 cells were labeled with NucBlue nuclear stain and incubated with the Cellevent caspase-3/7 green detection reagent (ThermoFisher, Uppsala, Sweden) and propidium iodide (ThermoFisher) to monitor apoptosis and necrosis during prolonged incubation with thrombin (1 U/ml), Yoda1 (20 µM), thrombin and Yoda1, or the apoptosis control staurosporine (10 μM, 5 μM and 0.25 μM). Time-lapse images were acquired with 10 min intervals for 5 h 20 min. The NucBlue signal for the entire field of view was defined as a region of interest (ROI) and the relative caspase-3/7 and propidium iodide fluorescence within this nuclear ROI was determined using the Fiji version of ImageJ and plotted over time. The accumulated caspase-3/7 and propidium iodide fluorescence for the indicated 25 min durations were calculated from the area under their respective curves (AUC), which is expressed as a percentage of the average fluorescence recorded in wells treated with 10 µM staurosporine.

### Statistical analysis

All data were summarized in Microsoft Excel and exported to GraphPad Prism software (GraphPad, La Jolla, CA; version 9 for macOS) for statistical analyses. Data distributions were analyzed and parametric or non-parametric statistical tests were employed accordingly. For parametric datasets consisting of two groups, two-tailed paired or unpaired Student's *t*-tests were used as appropriate. For parametric paired data sets consisting of three or more groups, one-way ANOVA with Tukey's multiple comparisons were used. For non-parametric paired datasets consisting of two groups, the Wilcoxon matched-pairs signed rank test was used; and for non-parametric unpaired datasets consisting of two groups, the Mann–Whitney test was used. For non-parametric paired datasets consisting of three or more groups, the Friedman test with Dunn's multiple comparisons was used; and for non-parametric unpaired datasets consisting of three or more groups, the Kruskal–Wallis test with Dunn's multiple comparisons were used.

## Supplementary Material

Supplementary information

Reviewer comments
