# Supplementary material for: Piezo1 activation attenuates thrombin-induced blebbing in breast cancer cells
Source: J Cell Sci. 2022 Apr 1;135(7):jcs258809. doi: 10.1242/jcs.258809 (PMC9016622; doi:10.1242/jcs.258809)
Supplement: Supplementary information [file joces-135-258809-s1.pdf]

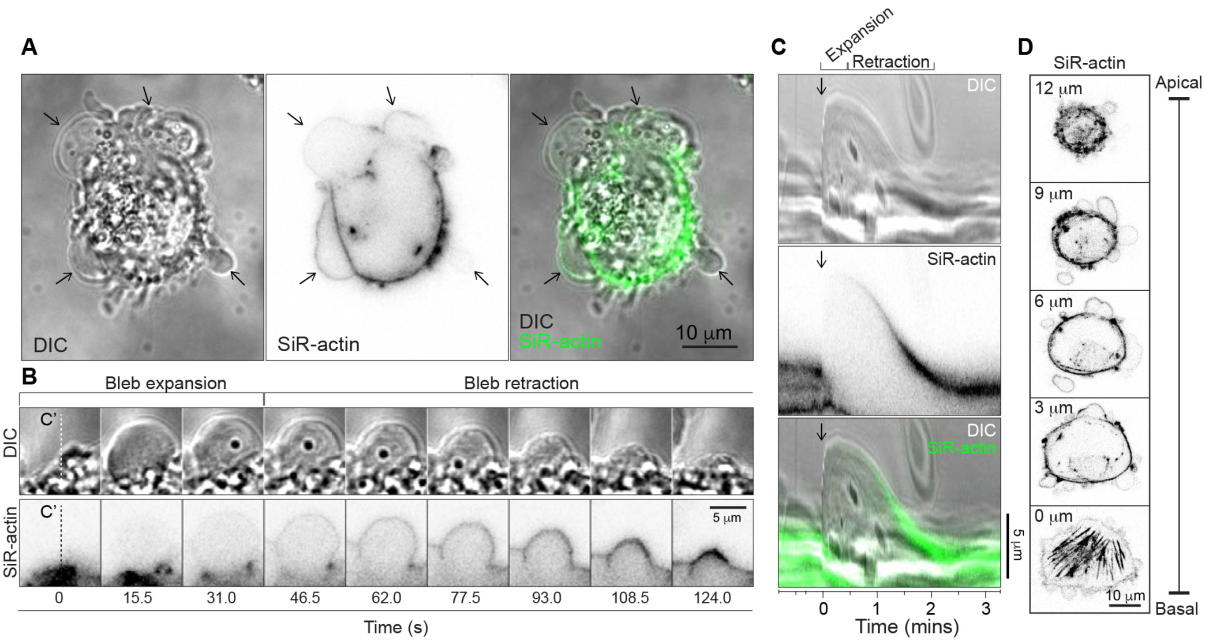

Fig. S1. Characterization of spontaneous blebbing in MDA-MB-231 breast cancer cells.
A, DIC and confocal microscopy of a blebbing MDA-MB-231 cell with F-actin visualized using the fluorescent SiR-actin probe. Arrows indicate blebs at various stages of F-actin recruitment (see also Movie 1). **B,** Time-lapse sequences of the expansion and retraction of a single bleb and associated F-actin recruitment (from the cell shown in panel A). **C,** Kymograph of the expansion and retraction of an individual bleb plotted from the DIC and SiR-actin signals under the dashed lines (C') in (B). Arrows indicate start of bleb expansion. **D,** Confocal z-stack images of the SiR-actin signal from the basal to apical cell surfaces of the cell shown in (A). .

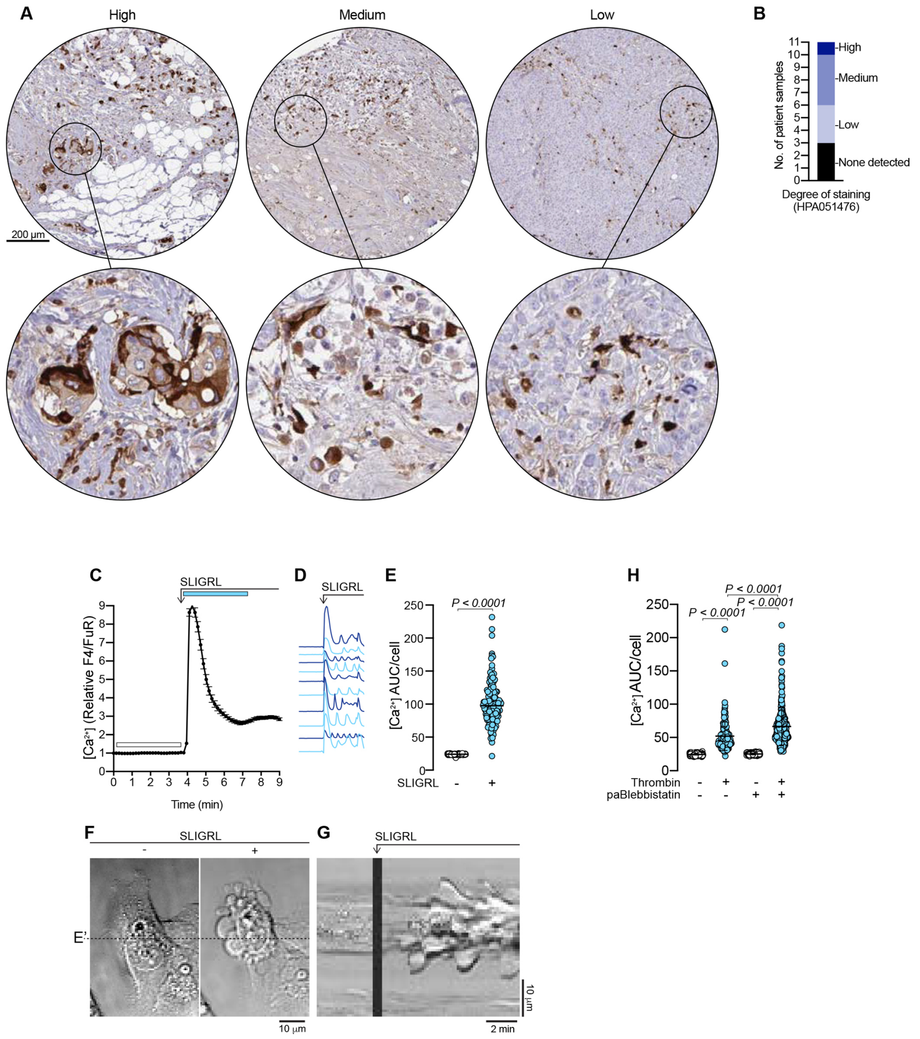
A
High
Medium
Low
200 µm
B
No. of patient samples
High
Medium
Low
None detected
Degree of staining
(HPA051476)
C
SLIGRL
[Ca2+] (Relative F4/FuR)
Time (min)
D
SLIGRL
E
P < 0.0001
[Ca2+] AUC/cell
SLIGRL
H
P < 0.0001
P < 0.0001
P < 0.0001
[Ca2+] AUC/cell
Thrombin
paBlebbistatin
F
SLIGRL
E'
10 µm
G
SLIGRL
10 µm
2 min

Fig. S2. Thrombin in breast cancer tissue, and effects of PAR2 stimulation on MDA-MB-231 breast cancer cells.

Thrombin expression data from the Human Protein Atlas (HPA). **A,** Examples of the breast cancer sections that revealed high, medium and low degrees of staining with the thrombin antibody HPA051476. Staining patterns are described by the HPA as cytoplasmic and membranous. The breast cancer sections are annotated as follows in the HPA database. The example of high degree thrombin staining was biopsied from patient 2565 lobular carcinoma (M-85203). The medium degree thrombin staining example was biopsied from patient 1910 with ductal carcinoma (M-85003), and the low degree thrombin staining example was biopsied from patient 4193, also with ductal carcinoma. **B,** Degree of immunohistochemistry staining observed with the thrombin antibody HPA051476 in 11 breast cancer patients. All thrombin immunohistochemistry data can be viewed at the following address: https://www.proteinatlas.org/ENSG00000180210-F2/pathology/breast+cancer#img from v19.3.proteinatlas.org. Image credit: Human Protein Atlas. **C,** PAR2 agonist (SLIGRL; 10 µM) induced changes in cytosolic Ca^{2+} (F4/FuR ratio) in MDA-MB-231 cells. The plot represents the mean ± s.e.m. over time for n = 133 cells. **D,** Individual F4/FuR signals from ten MDA-MB-231 cells in response to SLIGRL (10 µM). Notably, SLIGRL induced Ca^{2+} oscillations that were not typically observed in thrombin-treated cells (see Fig. 2 for comparison). **E,** Quantification of Ca^{2+} AUC /cell before and after SLIGRL addition, calculated from the relative F4/FuR plots for the durations indicated by the color-coded bars in (A). The *P*-value was determined by Wilcoxon matched-pairs signed rank test. **F,** DIC images of an MDA-MB-231 cell before and after SLIGRL stimulation. **G,** Kymograph plotted from the DIC time-lapse signal recorded under the dashed line (E') in (D). **H,** Quantification of Ca^{2+} AUC/cell before and after thrombin addition with or without para-amino-blebbistatin pretreatment, calculated from their respective F4/FuR plots. *P*-values were determined by Kruskal Wallis test, with Dunn's multiple comparisons.

Journal of Cell Science • Supplementary information

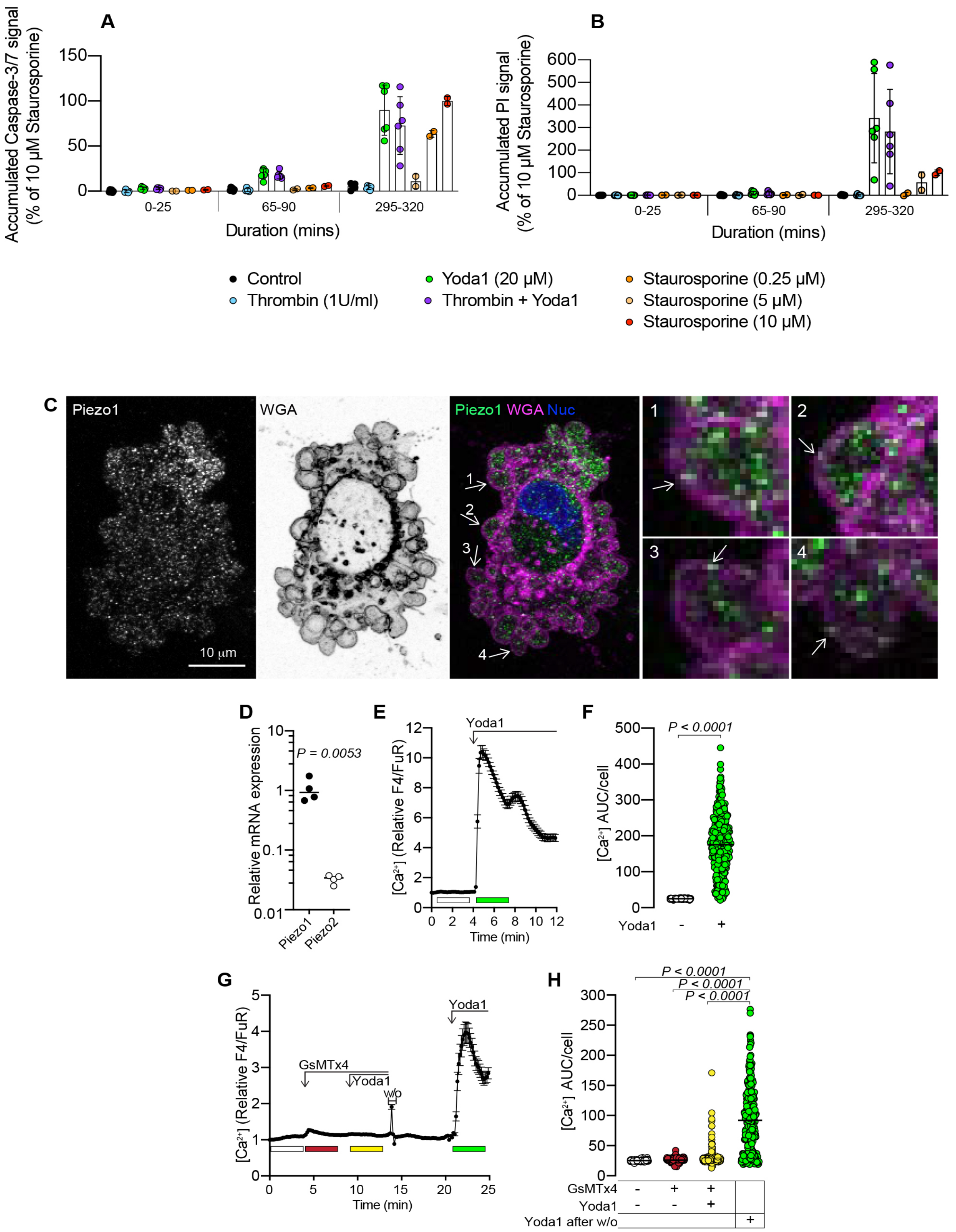

Journal of Cell Science • Supplementary information

Fig. S3. Cell death analysis, and Piezo1 distribution and Yoda1-mediated activation in MDA-MB-231 cells.

A, Accumulated caspase-3/7 fluorescence signal in MDA-MB-231 cells treated with thrombin (1U/ml), Yoda1 (20 µM), thrombin and Yoda1, or the apoptosis inducing agent staurosporine (positive control) at indicated concentrations, during the indicated time periods. **B,** Accumulated propidium iodide (PI) fluorescence signal for the MDA-MB-231 cells treated in (A). Scatterplots represent $n = 6$ experiments, except for the staurosporine treatments where $n = 2$ for each concentration. **C,** Confocal microscopy image of a thrombin-stimulated blebbing MDA-MB-231 cell immunostained for Piezo1 and counterstained with WGA for the plasma membrane, and NucBlue for nuclei. The regions indicated by arrows and enlarged in panels 1-4 represent individual blebs. Piezo1 puncta are distributed within the cytosolic part of the bleb, but examples of overlap with the WGA-positive membrane are also present. **D**, Relative expression of Piezo1 and Piezo 2 mRNA in MDA-MB-231 cells as determined by qPCR. The *P*-value was determined by two-tailed Student's *t*-test. **E,** Yoda1 (20 µM) induced changes to cytosolic Ca^{2+} (F4/FuR signal) in MDA-MB-231 cells recorded by confocal microscopy. The plot represents the mean ± s.e.m. over time for $n = 148$ cells from one experiment. **F,** Quantification of Ca^{2+} AUC /cell before and after Yoda1 addition, calculated from the relative F4/FuR plots for the durations indicated by the color-coded bars in (A). The *P*-value was determined by Wilcoxon matched-pairs signed rank test, for $n = 377$ cells from three experiments. **G**, Changes to cytosolic Ca^{2+} (F4/FuR signal) in response to Yoda1 (20 µM) in the presence and absence of GsMTx4 (10 µM) recorded by confocal microscopy. The plot represents the mean ± s.e.m. from $n = 108$ cells from one experiment. **H,** Quantification of Ca^{2+} AUC/cell calculated for the durations indicated by the color-coded bars in (C). The *P*-value was determined by the Friedman test, with Dunn's multiple comparisons, for $n = 285$ cells from two experiments.

Journal of Cell Science • Supplementary information

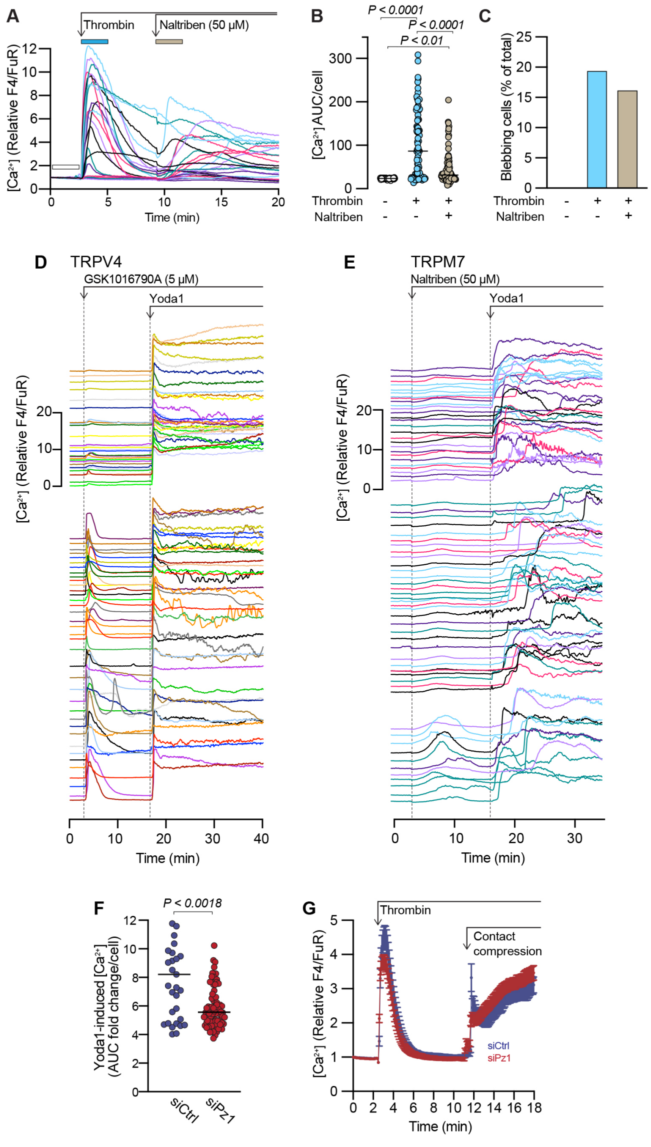

Journal of Cell Science • Supplementary information

Fig. S4. Effects of the TRPM7 activator naltriben on thrombin-stimulated blebbing. A, Changes to cytosolic Ca^{2+} (relative F4/FuR ratio) in cells before and after thrombin (1U/ml), and naltriben (50 μM) addition. Plots from individual cells (*n* = 24) from one experiment are presented. **B,** Quantification of Ca^{2+} AUC/cell from the relative F4/FuR plots for the durations indicated by the color-coded bars in (A) (*n* = 85 cells from three experiments). **C,** Quantification of blebbing in MDA-MB-231 cell populations before and after sequential addition of thrombin and naltriben. Bars represent the percentage of blebbing cells from one experiment. *P*-values in (B) were determined by Friedman test with Dunn's multiple comparisons. **D,** Changes to cytosolic Ca^{2+} (relative F4/FuR ratio) in cells before and after GSK1016790A (5 μM) addition, followed by Yoda1. Plots from individual cells (*n* = 67 cells) from one experiment are presented. Note that the plots are divided into an upper and lower group, where the upper 29 cells demonstrate low or no sensitivity to GSK1016790A, while the lower 38 cells demonstrate a clear transient Ca^{2+} elevation, while both groups display a robust and sustained response toYoda1.E,Changes to cytosolic Ca^{2+} (relativeF4/FuR ratio)in cells before and after naltriben (50 μM) addition, followed by addition of Yoda1. Plots from individual cells (*n* = 70 cells) from one experiment are presented. Note that the plots are divided into an upper, middle and lower group, where the upper (27 cells) and middle (32 cells) demonstrate low or no sensitivity to naltriben, while the lower 11 cells demonstrate a slow and transient Ca^{2+} elevation. In contrast, while all three groups display a response to Yoda1, examples of slower Ca^{2+} rises and delayed responses are seen predominantly in the middle and lower groups. **F,** Quantification of Yoda1-induced Ca^{2+} AUC fold change/cell from relative F4/FuR plots for n = 33 siCtrl cells, and 86 siPiezo1 cells. The *P*-value was determined by the Mann-Whitney test. **G,** Effect of treatment with thrombin (1 U/ml) followed by contact compression on cytosolic Ca^{2+} in siCtrl and siPiezo1 transfected cells. The plot represents the mean ± s.e.m. (*n* = 52 siCtrl and *n* = 77 for siPz1 transfected cells).

Journal of Cell Science • Supplementary information

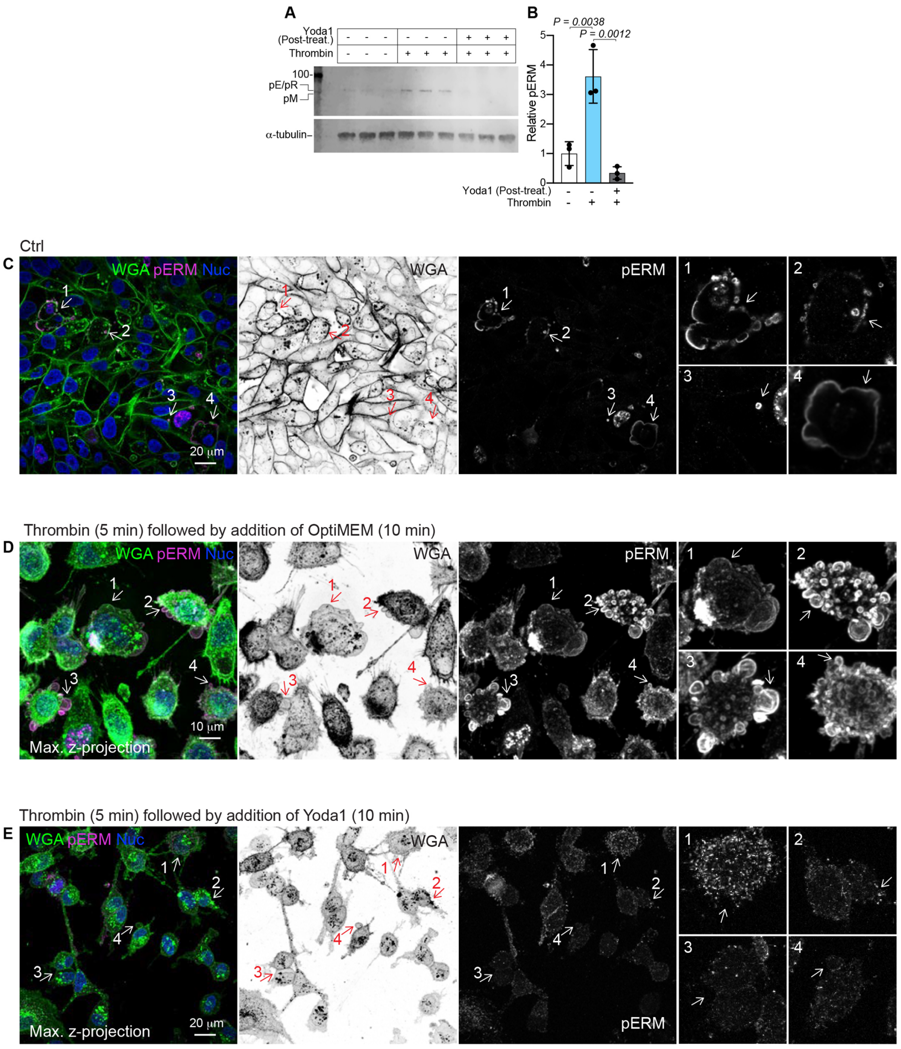

Journal of Cell Science • Supplementary information

Fig. S5. Yoda1 impairs thrombin-induced ERM phosphorylation.

A, Immunoblotting for phosphorylated ERMs in MDA-MB-231 cells stimulated with or without thrombin (1 U/ml), and subsequently (5 min later) treated with or without Yoda1 (20 μM) for 15 mins. **B,** Quantification of pERM band intensities relative to α-tubulin loading controls for the samples in (A). Bars represent the mean ± s.d. for three replicates. *P*-values were determined by one-way ANOVA, with Tukey's multiple comparisons. **C-E.** Confocal microscopy image of MDA-MB-231 cell immunostained for pERMs and counterstained with WGA for the plasma membrane, and NucBlue for nuclei. **C.** Examples of control (Ctrl) cells treated with OptiMEM (15 min). The regions indicated by arrows and enlarged in panels 1-4 illustrate examples of pERM enrichment, which are specifically observed in blebbing, but not adjacent non-blebbing cells. **D.** Examples of cells treated with thrombin (5 min) and then with OptiMEM (10 min). The regions indicated by arrows and enlarged in panels 1-4 detail examples of cells with multiple blebs, which are clearly detected by pERM staining. **E.** Examples of cells treated with thrombin (5 min) and then Yoda1 (10 min). The regions indicated by arrows and enlarged in panels 1-4 reveal how the pERM signal no longer appears to be predominantly membrane associated, and is instead reduced in intensity and more punctate in appearance. Note that the intensity of the pERM immunofluorescence has been enhanced in (E) to aid in visualizing the altered staining distribution. Also, while (D) and (E) are maximum intensity z-projections (approximate z-height of 16 μm), to better visualize the contrast between pERM positive and negative cells, only a single z-plane (approximate z-height 1μm) is presented in (C).

Journal of Cell Science • Supplementary information

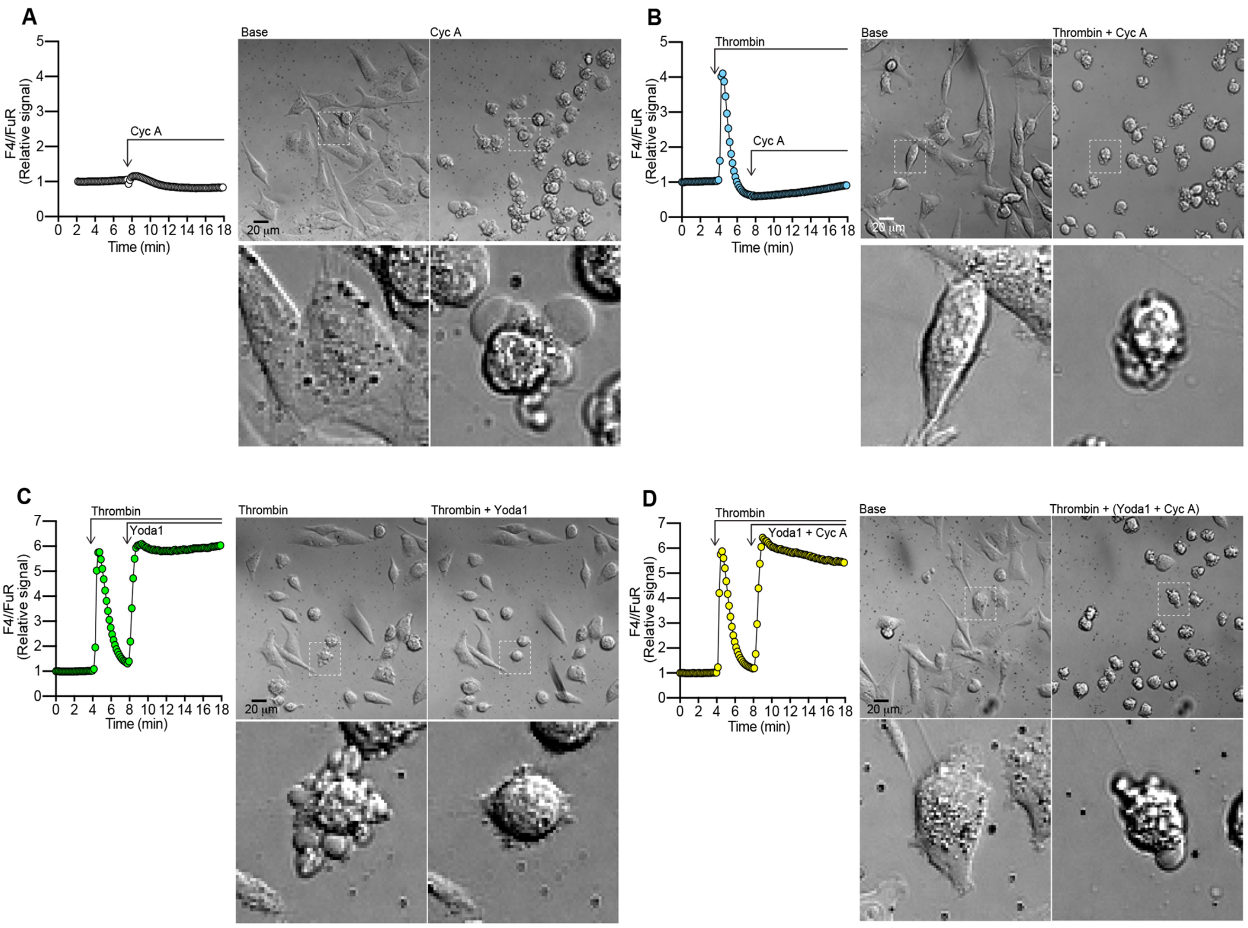

Fig. S6. PP1/PP2A phosphatase inhibition induces blebbing.

A-D, Wholefield analysis of cytosolic Ca^{2+} changes (F4/FuR signal) as recorded by confocal microscopy before and after the indicated treatments with either Calyculin A alone (Cyc A; 50 nM) (A), thrombin (1 U/ml), followed by Cyc A (50 nM) (B), thrombin (1 U/ml) followed by Yoda1 (20 µM) (C) or thrombin (1 U/ml), followed by Yoda1 (20 µM) together with Cyc A (50 nM) (D). Individual frames from DIC time-lapse sequences illustrate cell morphology before and after treatment; insets represent high magnification examples of cells framed with dashed lines in the main images.

Journal of Cell Science • Supplementary information

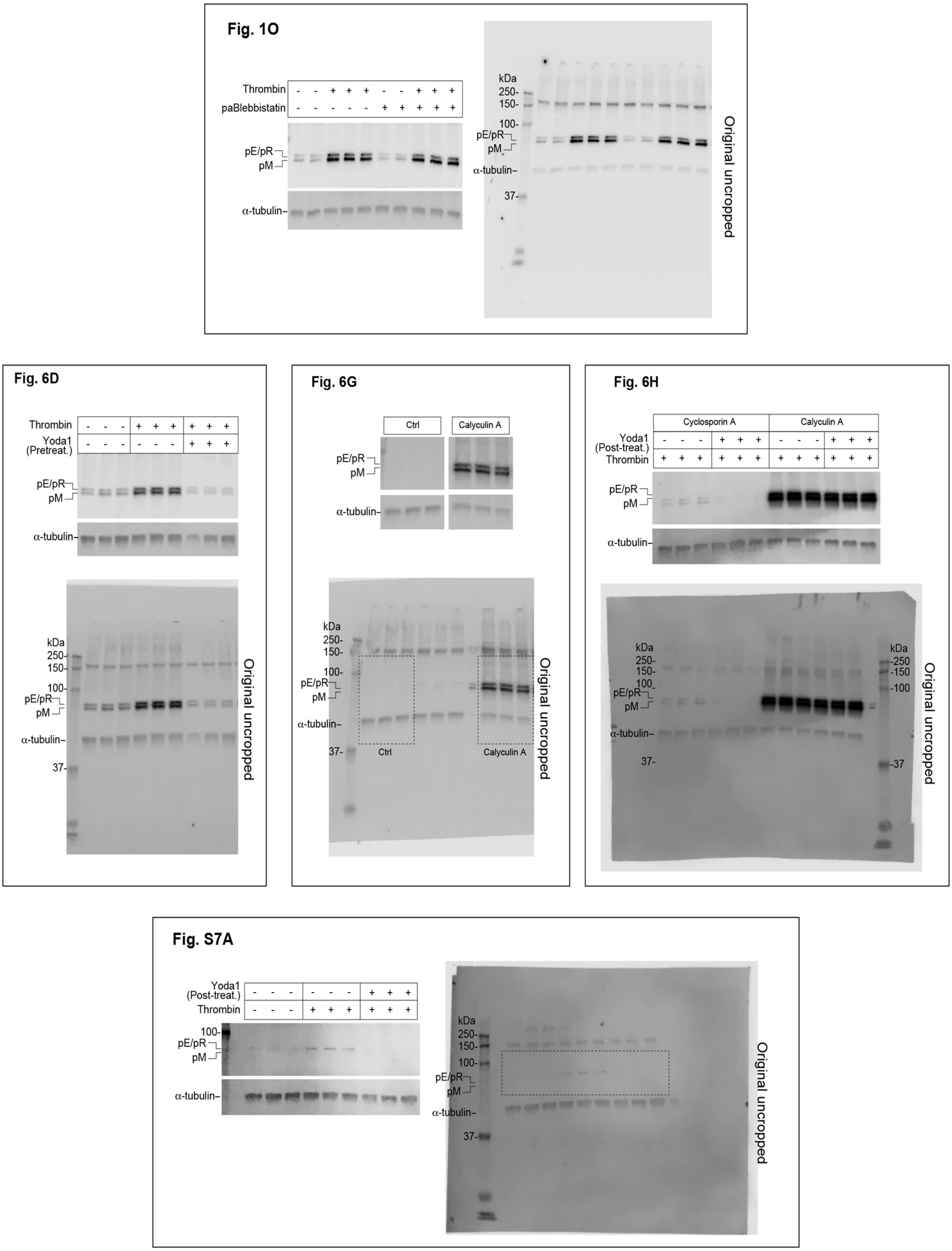

Fig. S7. Western blot transparency

Journal of Cell Science • Supplementary information

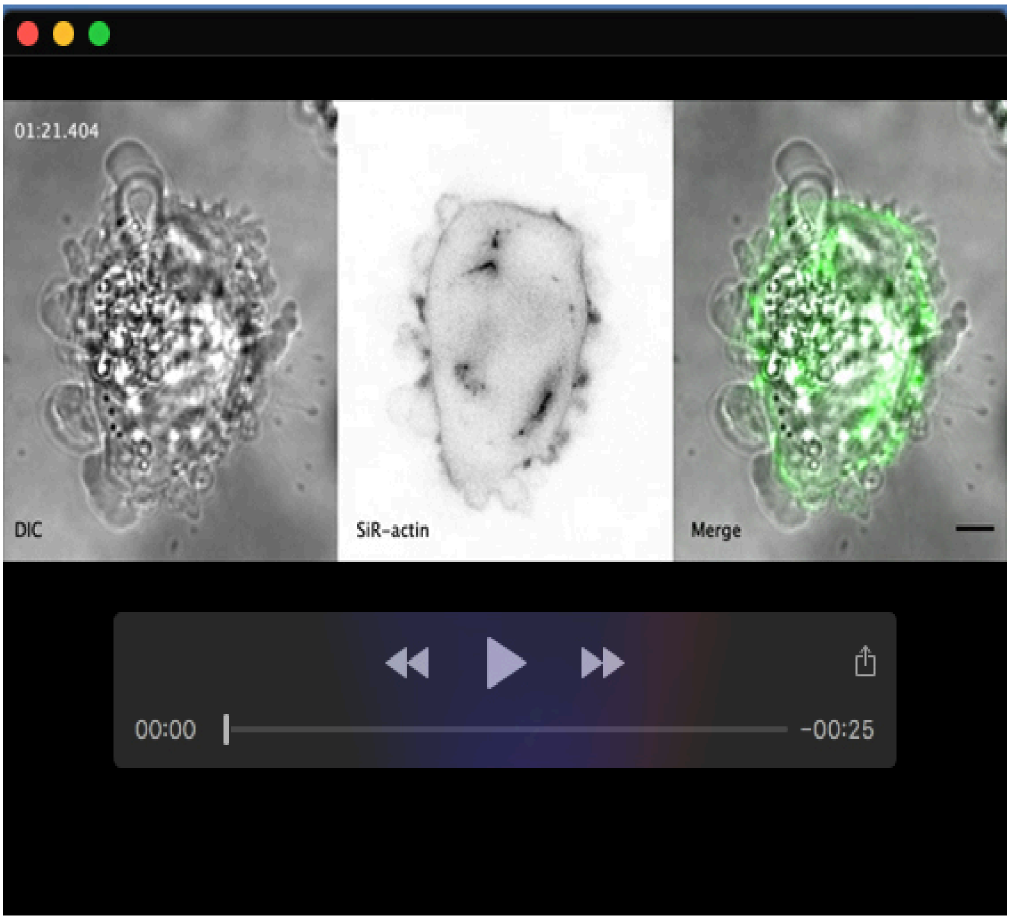

Movie 1. Plasma membrane and actin dynamics in a spontaneously blebbing MDA-MB-231 cell. Time-lapse series of DIC and confocal microscopy recordings for the blebbing MDA-MB-231 cell in Fig. 1A with F-actin visualized using the fluorescent SiR-actin probe. The format of the digital timer is min:sec.msec. Scale bar; 10 µm.

Journal of Cell Science • Supplementary information

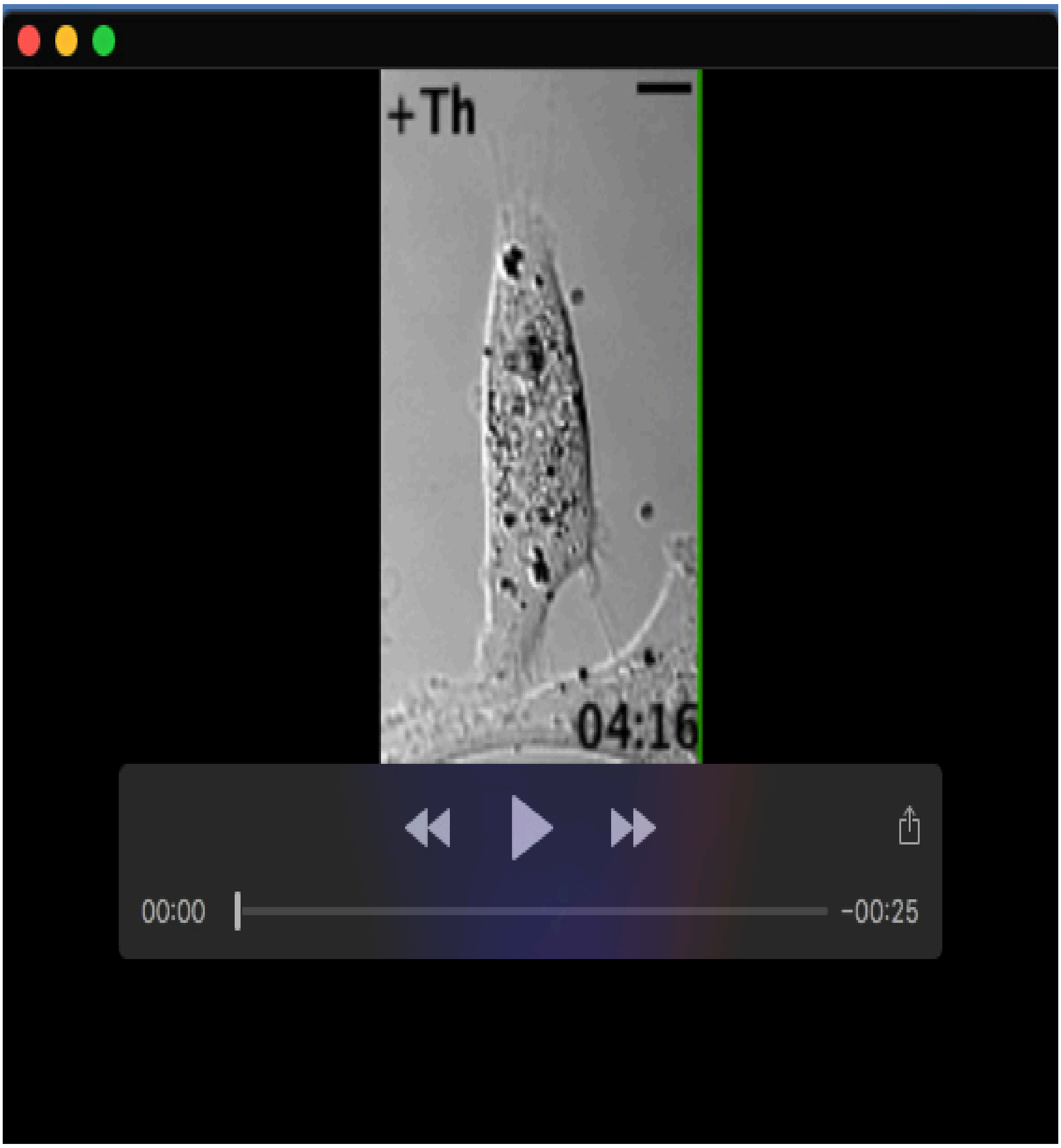

Movie 2. Thrombin-induced blebbing in an MDA-MB-231 cell. DIC time-lapse recordings of thrombin-induced blebbing for the MDA-MB-231 cell in Fig. 2E, F. The addition of thrombin (1 U/ml) is indicated as +Th. The format of the digital timer is min:sec. Scale bar; 10 µm.

Journal of Cell Science • Supplementary information

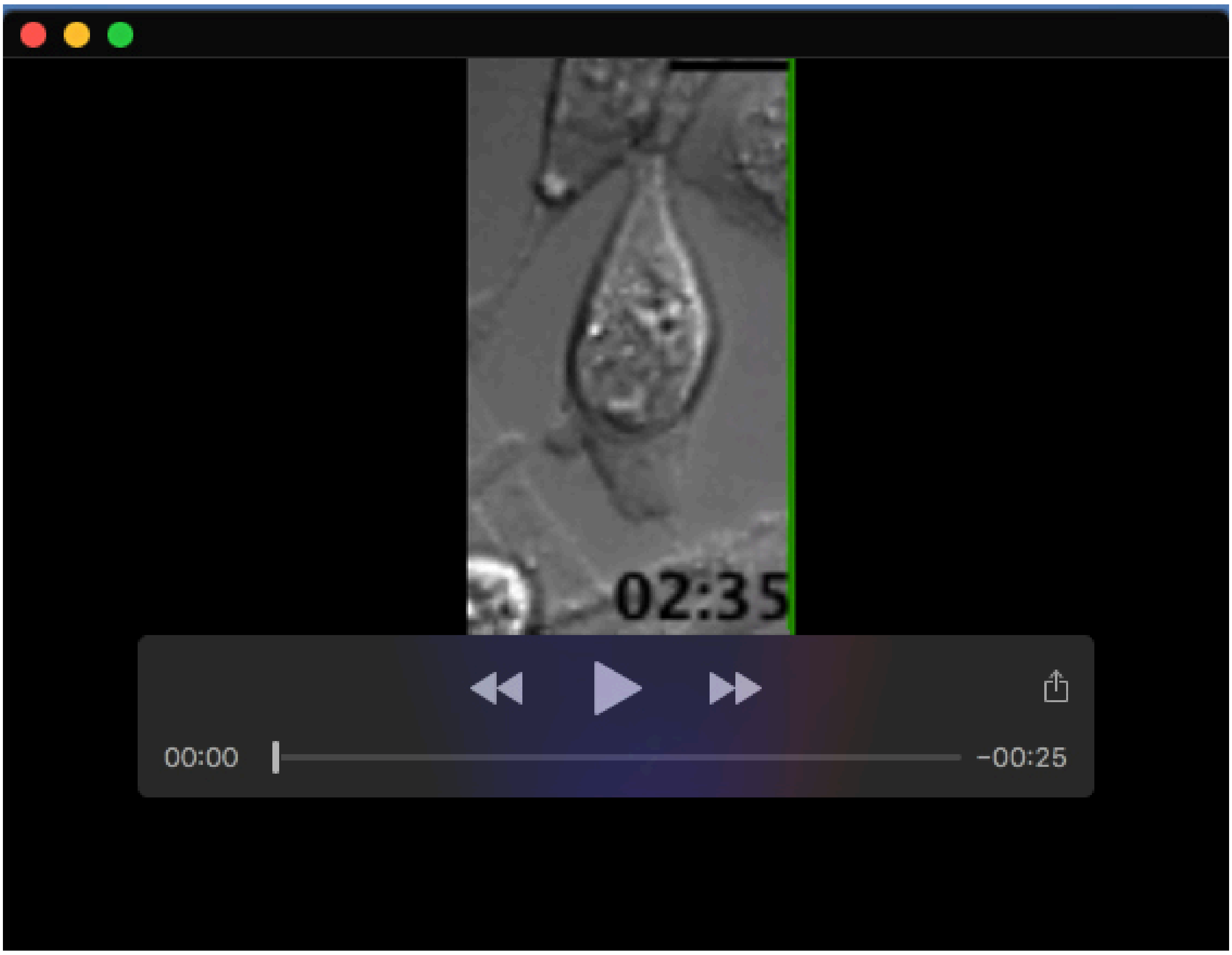

Movie 3. Contact compression attenuates thrombin-induced blebbing in an MDA-MB-231 cell. DIC time-lapse recordings of contact compression attenuation of thrombin-induced blebbing for the MDA-MB-231 cell in Fig. 3F, G. The addition of thrombin (1 U/ml) is indicated as +Th, and the application of contact compression as +CC. Removal of the compression pillar is indicated as NC (no compression). The format of the digital timer is min:sec. Scale bar; 10 µm

Journal of Cell Science • Supplementary information

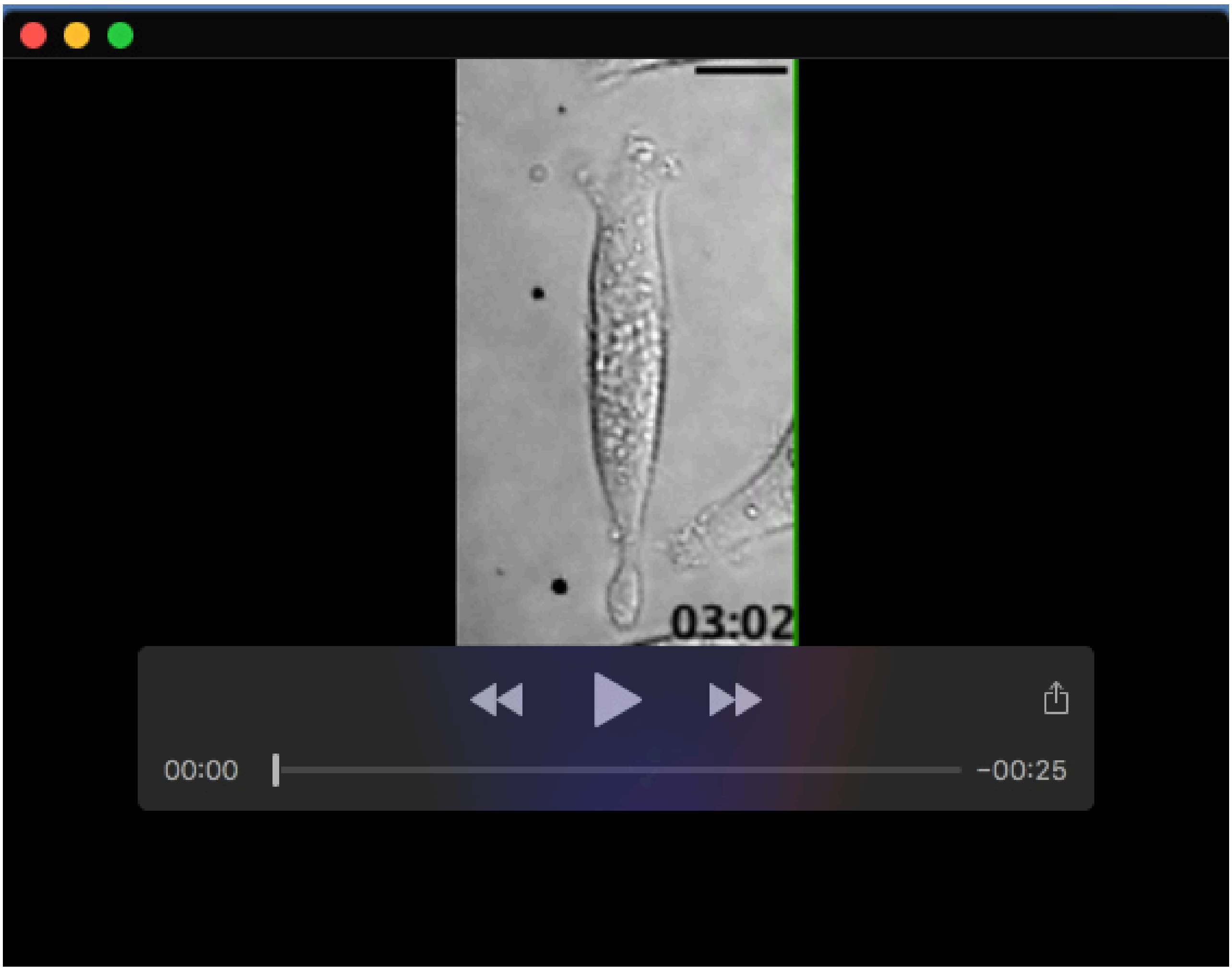

Movie 4. Yoda1 attenuated blebbing in a thrombin-treated MDA-MB-231 cell. DIC time-lapse recordings of the MDA-MB-231 cell from Fig. 5C. The addition of thrombin (1 U/ml), DMSO and Yoda1 (20 µM) are indicated as +Th, +DMSO, and +Yoda1. The format of the digital timer is min:sec. Scale bar; 10 µm

Journal of Cell Science • Supplementary information
