# Supplementary material for: Piezo1 activation attenuates thrombin-induced blebbing in breast cancer cells
Source: J Cell Sci. 2022 Apr 1;135(7):jcs258809. doi: 10.1242/jcs.258809 (PMC9016622; doi:10.1242/jcs.258809)
Supplement: Reviewer comments [file joces-135-258809_review_history.pdf]

Journal of Cell Science

Piezo1 activation attenuates thrombin-induced blebbing in breast cancer cells

Paul O'Callaghan, Adam Engberg, Olle Eriksson, Nikos Fatsis-Kavalopoulos, Christina Stelzl, Gonzalo Sanchez, Olof Idevall-Hagren and Johan Kreuger
DOI: 10.1242/jcs.258809

Editor: Andrew Ewald

Review timeline

Original submission:	20 April 2021
Editorial decision:	14 June 2021
First revision received:	13 December 2021
Editorial decision:	24 January 2022
Second revision received:	4 February 2022
Accepted:	22 February 2022

Original submission

First decision letter

MS ID#: JOCES/2021/258809

MS TITLE: Piezo1 activation attenuates thrombin-induced blebbing in breast cancer cells

AUTHORS: Paul O'Callaghan, Adam Engberg, Olle Eriksson, Nikos Fatsis-Kavalopoulos, Christina Stelzl, Gonzalo Sanchez, Olof Idevall-Hagren, and Johan Kreuger
ARTICLE TYPE: Research Article

We have now reached a decision on the above manuscript.

To see the reviewers' reports and a copy of this decision letter, please go to: https://submit-jcs.biologists.org and click on the 'Manuscripts with Decisions' queue in the Author Area. (Corresponding author only has access to reviews.)

As you will see, the reviewers share enthusiasm for the topic and scope of the study and find the conclusions interesting; however, they raise a number of substantial criticisms that prevent me from accepting the paper at this stage. There are recurrent questions about how calcium increases could mediated both induction and repression of blebbing, concerns about experimental support for some stated conclusions, and a sense that the study would benefit significantly from some additional experiments. They suggest, however, that a revised version might prove acceptable, if you can address their concerns. If you think that you can deal satisfactorily with the criticisms on revision, I would be pleased to see a revised manuscript. We would then return it to the reviewers.

We are aware that you may be experiencing disruption to the normal running of your lab that makes experimental revisions challenging. If it would be helpful, we encourage you to contact us to discuss your revision in greater detail. Please send us a point-by-point response indicating where you are able to address concerns raised (either experimentally or by changes to the text) and where you will not be able to do so within the normal timeframe of a revision. We will then provide further guidance. Please also note that we are happy to extend revision timeframes as necessary.

© 2022. Published by The Company of Biologists under the terms of the Creative Commons Attribution License (https://creativecommons.org/licenses/by/4.0/).

Please ensure that you clearly highlight all changes made in the revised manuscript. Please avoid using 'Tracked changes' in Word files as these are lost in PDF conversion.

I should be grateful if you would also provide a point-by-point response detailing how you have dealt with the points raised by the reviewers in the 'Response to Reviewers' box. Please attend to all of the reviewers' comments. If you do not agree with any of their criticisms or suggestions please explain clearly why this is so.

Reviewer 1

Advance summary and potential significance to field

This study reports thrombin-induction of breast cancer cell blebbing, a phenomenon that can be suppressed by low compressive stress and treatment of Yoda-1, an agonist of mechanosensitive ion channel PIEZO1.
These observations provide a new angle to understand how cell blebbing, an integral characteristic of amoeboid cell state, can be regulated by chemical and mechanical cues. The significance of this work will be enhanced if compelling mechanistic explanations can be provided to explain how thrombin and PIEZO1-mediated mechanotransduction works in this context.

Comments for the author

This manuscript by O'Callagan et al. investigates blebbing behavior of breast cancer cells. The authors reported that thrombin treatment induces blebbing of MDA-MB-231 cells, a breast cancer cell line. Thrombin treatment increases cytosolic calcium level. The authors showed that PAR1 and PAR2 agonists induced cell blebbing and phosphorylated of ERM proteins, which tethers the plasma membrane to actin. The authors showed that low level compressive stress reduces thrombin-dependent blebbing, whereas higher compressive stress induces blebbing. The authors showed that Yoda-1, an agonist of mechanosensitive ion channel PIEZO1, increases cytosolic calcium level and suppresses thrombin-induced blebbing. The authors showed that PIEZO1 knockdown increases thrombin-induced blebbing and the level of phosphorylated ERM.
Finally, the authors treated cells with CycA, which can inhibit PP1/PP2A phosphatase, and detected increased blebbing. Based on these data, the authors concluded that PIEZO1 activation suppresses thrombin-induced blebbing by activation of PP1/PP2A phosphatase to dephosphorylate ERM. This manuscript describes interesting phenomena, including thrombin induction of cell blebbing, which can be inhibited by low compressive stress, and a potential role of PIEZO1 in modulating this process. However, it is difficult to interpret these observations at a mechanistic level. There are several claims that are not supported by experimental data. The following points would need to be addressed before publication.
1. The most perplexing point relates to the effects of thrombin and PIEZO1 in regulating cytosolic calcium.
Thrombin treatment and Yoda-1 activation of PIEZO1 both increase cytosolic calcium. However, they led to opposite phenotypes, namely thrombin treatment increases blebbing, whereas PIEZO1 activation suppresses blebbing. The authors stated that calcium influx by PIEZO1 activates PP1/PP2A phosphatases, which dephosphorylates ERM thereby attenuate blebbing. Why does thrombin-dependent calcium level increase lead to blebbing? Are there spatiotemporal differences in the calcium signaling events induced by thrombin and PIEZO1 that can explain their opposite roles? The author discussed about calcium-independent role of PIEZO1, however, such mechanism was not explored as the manuscript is "calcium-centric".
2. The authors stated that an increase of phosphorylated ERM, induced by thrombin, increases blebbing whereas a decrease in phosphorylated ERM, induced by Yoda-1 treatment, decreases blebbing. However dynamic changes, rather than a simply increase or decrease, of phosphorylated ERM govern membrane blebbing. For example, the initiation phase of cell protrusion requires dephosphorylation of Ezrin, and actin-membrane release creates blebbing (Welf et al. Developmental Cell 2020). Therefore, the functional relevance of phosphorylated ERM level is not clear. In addition, Western blotting does not provide spatial information, which is critical to interpret the function of phosphorylated ERM as a membrane-actin linker.
Immunofluorescence staining and high-resolution imaging focusing on the blebbing areas are required to evaluate changes in ERM states.

© 2022. Published by The Company of Biologists under the terms of the Creative Commons Attribution License (https://creativecommons.org/licenses/by/4.0/).

3. There are places where correlations were claimed as causalities.
First, the fact that thrombin and PAR1/2 agonist treatment both led to calcium increase and blebbing does not prove thrombin signals through PAR1/2 – these data are correlations. Loss-of-function experiment (genetic or pharmacological inhibition of PAR1/2 in thrombin-treated cells) can help establish causality.
Second, the authors treated cells with CycA, which can inhibit PP1/PP2A phosphatase, and stated that phosphorylated ERM level is increased. As the authors pointed out, however, CycA also inhibits myosin light chain phosphatase. It is then not certain whether PP1/PP2A are mediating the effect of Yoda-1.
Third, even if PP1/PP2A mediates the effect of Yoda1/the function of PIEZO1, it is not certain PP1/PP2A, which have many substrates, works through dephosphorylate phosphorylated ERM to regulate blebbing.
4. The relevance of apoptosis-related data is not clear in this manuscript. How does Yoda-1 induced apoptosis help explain the blebbing behavior?
5. PIEZO1 localization on the plasma membrane is not sufficiently demonstrated. F-actin is not a plasma membrane marker. A definitive plasma membrane marker is required in Figure 5.
6. Vehicle control data are missing in Figure 7F, 7G
7. Is the effect of thrombin, Yoda-1, PIEZO1 etc. conserved in other types of breast cancer cell lines?
8. The author mentioned amoeboid cell state in a couple of places. It would be important to clarify that this manuscript studies blebbing behaviors but not amoeboid cell state, which include blebbing, as well as cell rounding, increased actomyosin contractility, and reduced cell adhesion to ECM. The latter three aspects are not studied in this work.

<u>Reviewer 2</u>

Advance summary and potential significance to field

In this manuscript, O'Callaghan et al. show that contact compression, which increases cytosolic Ca2+, attenuates thrombin-induced blebbing in MDA-MB-231 cells. Similarly, the Piezo1 agonist Yoda1, which induces a sharp increase in cytosolic Ca2+, abolishes thrombin-mediated blebbing. As expected, Piezo1 knockdown impairs the ability of Yoda1 to suppress thrombin-induced blebbing. Interestingly, this molecular intervention moderately exacerbates thrombin-induced blebbing in the absence of Yoda1. Lastly, the authors implicate the PP1A/PPA2 family of serine/threonine phosphatases in Yoda1-mediated reduction of thrombin-induced ERM phosphorylation. Overall, this is a well-executed and interesting albeit phenomenological study worthy of publication pending an appropriate revision.

Comments for the author

Comments:
1. The results of this manuscript are in line with recent data showing that increasing hydraulic resistance shifts the cell phenotype from amoeboid/blebbing to mesenchymal in confinement (PMID: 33893091; Zhao et al., Sci. Adv. 2021; 7: eabg4934). As shown in Fig. 7 of that study (PMID: 33893091), TRPM7 is the key sensor of hydraulic resistance. In view of this finding, the reviewer is wondering whether thrombin-induced cell blebbing is attenuated specifically by activation of Piezo1 or activation of other mechanosensitive ion channels (MICs), such as TRPM7 via the use of Naltriben or TRPV4 via GSK1016790A could also suppress thrombin-induced blebbing.
2. The authors should verify key findings with an additional cell line or at least acknowledge the use of a single cell line (MDA-MB-231) as a limitation.
3. Line 144: "spontaneous blebbing could be attenuated following initial gentle contact compression".
Inclusion of quantitative data would strengthen their manuscript.
4. Comparing Fig. 4F to Fig. 2C, it is evident that there is data variability. How do the authors explain this? Is this due to different cell passage numbers used in different experiments?
5. They only show Piezo 1 mRNA expression of siControl and siPiezo1 specimens. A western blot is needed to confirm knockdown at the protein level.
6. In Fig. 7F-G, data showing the effect of CycA on ERM phosphorylation would be helpful to the reader.

© 2022. Published by The Company of Biologists under the terms of the Creative Commons Attribution License (https://creativecommons.org/licenses/by/4.0/).

The demonstration of the involvement of PP1A/PP2A in this process is indirect, and the authors should soften their wording.

7. Statistical analysis: The authors should ensure the normal distribution of their data before performing the Student's t-test or one-way ANOVA. For instance, are the data in Fig. 2L normally distributed?

8. The authors should cite two additional articles which are directly relevant to this manuscript. First PMID: 32789173 shows that confinement promotes cell blebbing. Second, PMID: 31355337 shows that loss of cortical actin increases the sensitivity of MICs.

Reviewer 3

Advance summary and potential significance to field

The work by O'Callaghan et al. addresses the regulation of thrombin induced blebbing by Piezo1 in MDA MB-231 breast cancer cells. Using a custom-made "cell press" the authors describe the intriguing observation that gentle cell confinement (termed contact compression) leads to an inactivation of both spontaneous and thrombin-induced cell blebbing, whereas strong cell compression in the absence of thrombin induces pronounced cell blebbing as previously described in other cell types. The authors show that contact compression was accompanied with an increase of intracellular calcium levels which led them to study the involvement of the mechano-sensitive ion channel Piezo1 in thrombin-induced cell blebbing. Using the Piezo1 agonist Yoda1 and siRNA interference the authors identify a role of Piezo1 in modulating thrombin-induced cell blebbing. They propose that this process is regulated by PP1A/PP2A phosphatases upon Piezo1 activation by reducing thrombin-mediated ERM protein phosphorylation.

The article is clearly written and the data figures are presented in a detailed and consistent format easing the reading of the manuscript. The finding that Piezo1 activation inhibits thrombin-induced cell blebbing observation is interesting, however whether Piezo1 is involved in the mechanical cell response to contact compression remains unclear. It appears surprising that the authors have not tested the role of Piezo1 in contact compression to see if blebbing persists when reducing Piezo1 activity by the described siRNA approach and how this would link to calcium levels upon contact compression. Furthermore, the authors should clarify the following key points below related to the role of intracellular calcium levels and the function of PP1A/PP2A in regulating cell blebbing.

Comments for the author

- The authors argue that thrombin-induced calcium release from the ER stimulates cell blebbing. Contrary, a Piezo-induced calcium increase inhibits cell blebbing. This raises the question how intracellular calcium levels in the studied system regulate two opposing cell responses, activation of blebbing and inhibition of blebbing.
Is the calcium increase upon thrombin treatment necessary for cell blebbing or would an inhibition of the thrombin-induced calcium increase also promote cell blebbing? Would an increase of intracellular calcium levels after thrombin addition or contact confinement by alternative strategies (using for example Thagsigargin or Ionomycin) also lead to an inhibition of blebbing? Can the authors explain what leads to a gradual increase of intracellular calcium levels upon contact compression while Piezo1 activation triggers a transient calcium change? The authors could thereby strengthen the arguments on the suggested involvement of the mechano-sensitive activity of Piezo1 in regulating cell responses to compression, which would benefit to present the work more consistently. An involvement of Piezo1 in the cell blebbing response to larger deformation would further be interesting to assess.
- The authors suggest a role of PP1A/PP2A in controlling cell blebbing via phosphorylation of ERM proteins.
As the authors point out, CalyculinA is also causing changes in the phosphorylation of other proteins and most notably Myosin 2 proteins. It remains however unclear how Myosin 2 activity is affected by the treatments described in the paper. To which extend do thrombin and contact compression affect phosphorylation of Myosin 2 and how do these levels vary depending on the activation/inhibition of intracellular calcium levels?
Would also a non-specific increase of intracellular calcium lead to pERM increase? And does siRNA reduction of Piezo1 maintain ERM phosphorylation upon thrombin and contact compression?

© 2022. Published by The Company of Biologists under the terms of the Creative Commons Attribution License (https://creativecommons.org/licenses/by/4.0/).

- The "cell press" confinement device is indicated to perform cell compression in 5 μm steps during the first cell contact compression. This value appears very coarse to define it as a contact-compression. What is the standard cell height and nucleus height? This would allow to estimate the average cell deformation when a calcium influx is observed. The authors could potentially also use the nucleus area change as an additional parameter to calcium influx to assess cell contact compression more accurately. Furthermore, is the measured force dependent on the number of cells under the PDMS pillar or the activation of a contractile cell response upon compression? And was the PDMS functionalized or passivated and do the authors expect an influence of the PDMS material properties on the measured cell response?

Minor points:
- How do the authors explain that the attenuation of blebbing persisted after contact compression was released (Fig. 4E, and Movie 3)? Do cells upon release respond to thrombin? Does mild contact (and its release) lead to cell apoptosis?
- Thrombin addition leads to a rapid and transient calcium increase but apparently the blebbing response is delayed which would be interesting to comment.
- Do the two populations of spontaneously blebbing/non-blebbing cells behave differently upon thrombin treatment and contact compression?
- Is there a possible involvement of Piezo2 or other mechano-sensitive ion channels in the studied cell type as identified in different migratory cell types? A comment would be relevant to include.
- Fig. 1C seems to show the wrong time axis when compared to Fig. 1B.
- Fig. 2M shows a fraction of blebbing cells in the presence of blebbistatin and thrombin, how do the authors explain the occurrence of this blebbing cell fraction?
- Fig. 2N at which time points was ERM phosphorylation measured and does it persist over time?
- Fig. 3E spontaneous blebbing does not seem to correlate with a calcium increase, is this consistently observed and how to the authors argue about the occurrence of spontaneous blebbing?
- The thrombin-induced calcium spikes in Fig. 5G are consistent with Fig. 2D but not 4A, how do the authors explain the difference?
- Fig. 4F data for contact compression without thrombin should be included
- Fig. 6B x scale is missing
- Fig. 7A, what causes the faster decay upon Yoda treatment in this experiment which appears inconsistent with the decay in Fig. 6B?
- Several main figure panels could be moved to the supplementary information to reduce the number of main figures as they often show supporting information (including for example Fig. 1 panels, Fig. 3J which appears redundant to panel 3C and Fig. 6H,I).

First revision

<u>Author response to reviewers' comments</u>

Reviewer 1 Advance Summary and Potential Significance to Field:
This study reports thrombin-induction of breast cancer cell blebbing, a phenomenon that can be suppressed by low compressive stress and treatment of Yoda-1, an agonist of mechanosensitive ion channel PIEZO1. These observations provide a new angle to understand how cell blebbing, an integral characteristic of amoeboid cell state, can be regulated by chemical and mechanical cues. The significance of this work will beenhanced if compelling mechanistic explanations can be provided to explain how thrombin and PIEZO1- mediated mechanotransduction works in this context.

Reviewer 1 Comments for the Author:
This manuscript by O'Callaghan et al. investigates blebbing behavior of breast cancer cells. The authors reported that thrombin treatment induces blebbing of MDA-MB-231 cells, a breast cancer cell line. Thrombintreatment increases cytosolic calcium level. The authors showed that PAR1 and PAR2 agonists induced cell blebbing and phosphorylated of ERM proteins, which tethers the plasma membrane to actin. The authors showed that low level compressive stress reduces thrombin-dependent blebbing, whereas higher compressive stress induces blebbing. The authors showed that Yoda-1, an agonist of mechanosensitive ion channel PIEZO1, increases cytosolic calcium level and

© 2022. Published by The Company of Biologists under the terms of the Creative Commons Attribution License (https://creativecommons.org/licenses/by/4.0/).

suppresses thrombin-induced blebbing. The authors showed that PIEZO1 knockdown increases thrombin-induced blebbing and the level of phosphorylated ERM. Finally, the authors treated cells with CycA, which can inhibit PP1/PP2A phosphatase, and detected increased blebbing. Based on these data, the authors concluded that PIEZO1 activation suppresses thrombin-induced blebbing by activation of PP1/PP2A phosphatase to dephosphorylate ERM. This manuscript describes interesting phenomena, including thrombin induction of cell blebbing, which can be inhibited by low compressive stress, and a potential role of PIEZO1 in modulating this process. However, it is difficult to interpret these observations at a mechanistic level. There are several claims that are not supported by experimental data. The following points would need to be addressed before publication.

1. The most perplexing point relates to the effects of thrombin and PIEZO1 in regulating cytosolic calcium. Thrombin treatment and Yoda-1 activation of PIEZO1 both increase cytosolic calcium. However, they led to opposite phenotypes, namely thrombin treatment increases blebbing, whereas PIEZO1 activation suppresses blebbing. The authors stated that calcium influx by PIEZO1 activates PP1/PP2A phosphatases, which dephosphorylates ERM thereby attenuate blebbing.

a) Why does thrombin-dependent calcium level increase lead to blebbing?
b) Are there spatiotemporal differences in the calcium signaling events induced by thrombin and PIEZO1 that can explain their opposite roles?
c) The author discussed about calcium-independent role of PIEZO1, however, such mechanism was not explored as the manuscript is “calcium-centric”.

(a) Thrombin induces a number of changes that likely promote blebbing, and as described in the introduction the increase in Ca^{2+} activates calmodulin, which in turn activates MLC kinase, which will phosphorylate MLC2 and increase actomyosin contractility, an established promoter of blebbing. We provide additional support for this in our revised manuscript where we include immunocytochemistry data demonstrating that thrombin increases levels of phosphorylated MLC2 (Fig. 1N and 6F). An initial consequence of thrombin-mediated PAR activation is the phospholipase-mediated hydrolysis of PIP2 (to DAG and IP3), which will reduce actin-PM contact points, such as those stabilized via ERMs, allowing the actin-free plasma membrane (PM) to expand outwards in response to hydrostatic pressure. Importantly, while the myosin inhibitor para-aminoblebbistatin impaired blebbing (both spontaneous and thrombin-induced; Fig. 1M), neither thrombin-induced ERM phosphorylation (Fig. 1O, P) or Ca^{2+} (Fig. S3F) were reduced by myosin inhibition; thus underscoring the central importance of myosin activity for the thrombin-induced blebbing effect.

(b) We acknowledge that the opposing effects of increased cytosolic Ca^{2+} with regard to bleb induction, and bleb attenuation were not explicitly addressed in the original submission of our manuscript, and we have now added a more extensive discussion (paragraph 9 in our new Discussion on page 12, which starts “Notably, here we report elevated cytosolic Ca^{2+}….”.). With the method we use for imaging changes in cytosolic calcium it is difficult to obtain the resolution required to differentiate potential spatiotemporal differences, and while an approach like that used by Ellefsen et al. (Ellefsen et al., 2019) permits analysis of Piezo1-dependent Ca^{2+} influx at discrete sites on the PM, these events are generated by local endogenous increases in actomyosin traction forces, while Yoda1-stimulation and contact compression stimulated a more robust whole-cell Ca^{2+} response. Nonetheless, we now outline how spatiotemporal differences with respect to Ca^{2+} influx via Piezo1 at the PM close to sites of actin-PM contact versus the recruitment of internally ER stored Ca^{2+} via thrombin could contribute to the contrasting effects. Additionally, we draw attention to the fact that the Ca^{2+} response to thrombin is transient and short-lived, while typically a more sustained Ca^{2+} elevation is observed for Yoda1-induced Piezo1 activation, and contact compression. In this regard we cite two important studies (Dolmetsch et al., 1997, Timmerman et al., 1996) where the capacity for the cell to distinguish between different amplitudes and durations of Ca^{2+} responses results in the selective activation of specific downstream signaling and transcriptional events (please see page 12).

© 2022. Published by The Company of Biologists under the terms of the Creative Commons Attribution License (https://creativecommons.org/licenses/by/4.0/).

(c) Our main rational for discussing the possibility of a Ca^{2+}-independent effect of Piezo1 activation is the fact that we cannot directly couple the Yoda1-mediated reduction of thrombin-induced pERMs to a known mechanism that requires an increase in Ca^{2+}. As mentioned, only one Ca^{2+}-dependent PP1/PP2A phosphatase has been identified, and while we speculate that this is a potential candidate for the observed effects, we currently cannot confirm this. Piezo1 channel activation results in conformational changes, and given that Piezo1 rely on phosphoinositides (PIs) for its activation (Borbiro et al., 2015), it is conceivable that channel opening could result in local changes to the availability of PI components, which could impact on other PI-dependent complexes such as actin-PM contact sites. Nonetheless, to our knowledge the vast majority of Piezo1-mediated effects are a result of elevated Ca^{2+}, and future work will be required to investigate Ca^{2+}-independent effects of Piezo1 activation.

2. The authors stated that an increase of phosphorylated ERM, induced by thrombin, increases blebbing, whereas a decrease in phosphorylated ERM, induced by Yoda-1 treatment, decreases blebbing. However, dynamic changes, rather than a simply increase or decrease, of phosphorylated ERM govern membrane blebbing. For example, the initiation phase of cell protrusion requires dephosphorylation of Ezrin, and actin-membrane release creates blebbing (Welf et al. Developmental Cell 2020). Therefore, the functional relevance of phosphorylated ERM level is not clear. In addition, Western blotting does not provide spatial information, which is critical to interpret the function of phosphorylated ERM as a membrane-actin linker. Immunofluorescence staining and high-resolution imaging focusing on the blebbing areas are required to evaluate changes in ERM states.

We have now included new data to address this concern. We have performed immunocytochemistry for phosphorylated ERMs and used WGA to visualize the PM in control cells, and in cells treated with thrombin, with and without subsequent Yoda1 treatment (presented in the new Fig. 1 and in Fig. S7). We demonstrate that thrombin-induced pERMs are selectively enriched within the bleb membrane (Fig. 1Q, and Fig. S7D); essentially undetectable in non-blebbing Ctrl cells (Fig. S7C); and reduced, and no longer membrane-associated following Yoda1 treatment (Fig. S7E).

In our revised text we now include a discussion of this important study by Welf et al. (Discussion, page 9, paragraph 2). Notably, in their work ezrin dephosphorylation is controlled by photoactivatable, plasma membrane (PM) recruitment of the PRL3 phosphatase, causing local ezrin dephosphorylation, and blebbing due to dissociation of actin-PM contacts. However, while ezrin dephosphorylation is sufficient to induce blebbing, it is not the only means by which bleb expansion can occur, and here we propose a related, and closely connected event, whereby thrombin activation of PARs would lead to PIP2 hydrolysis, and therefore loss of PIP2 bindings sites for pERMs at the PM, which would destabilize actin-PM contacts and lead to blebbing.

3. There are places where **correlations were claimed as causalities.**

First, the fact that thrombin and PAR1/2 agonist treatment both led to calcium increase and blebbing does not prove thrombin signals through PAR1/2 – these data are correlations. Loss-of-function experiment (genetic or pharmacological inhibition of PAR1/2 in thrombin-treated cells) can help establish causality.

Our main objective for including the PAR1 and PAR2 agonists was to rule out the possibility that the bleb-inducing effect of thrombin was not directly related to a specific signaling event, but rather through the enzyme's general activity as a protease, which could potentially promote cell detachment by cleaving other cell surface proteins involved in adhesion (similar to trypsin). As the PAR1 and PAR2 agonists are short peptide sequences, identical to the tethered ligands generated by thrombin-mediated activation of their respective PARs, they lack protease activity and confirm that PAR1 or PAR2 activation alone is sufficient to induce blebbing. We have now included a statement outlining this rationale (Page 3).
Vanderboor et al. further demonstrate that thrombin can also induce blebbing via PAR4, and this work is cited in our discussion (Vanderboor et al., 2020). Consequently, while we agree that loss-of-function experiments would confirm causality, we would presumably need to simultaneously inhibit/knock-down multiple PAR family members to confirm that PAR activity is essential for the bleb-inducing effect of thrombin. Therefore, we would respectfully argue that as PAR1 and PAR2 are established thrombin-sensitive receptors, and given that agonists of both PAR1 and PAR2

© 2022. Published by The Company of Biologists under the terms of the Creative Commons Attribution License (https://creativecommons.org/licenses/by/4.0/).

reliably replicated both the Ca^{2+} and bleb inducing effects of thrombin, we suggest that our stated conclusion that *"…thrombin, as well as PAR1 and PAR2 agonists, induce blebbing in MDA-MB-231 breast cancer cells."* is a reasonable claim.

Second, the authors treated cells with CycA, which can inhibit PP1/PP2A phosphatase, and stated that phosphorylated ERM level is increased. As the authors pointed out, however, CycA also inhibits myosin light chain phosphatase. It is then not certain whether PP1/PP2A are mediating the effect of Yoda-1.
Third, even if PP1/PP2A mediates the effect of Yoda1/the function of PIEZO1, it is not certain PP1/PP2A, which have many substrates, works through dephosphorylate phosphorylated ERM to regulate blebbing.

We agree that given the potential broad effects of CycA-mediated inhibition of PP1/PP2As our data does not provide conclusive evidence that PP1/PP2As are effectors of Piezo1 activation, or that PP1/PP2A-mediated pERM dephosphorylation is the only mechanism contributing to bleb attenuation. Based on this, and on the advice of another of the reviewers we have now reworded and softened our claims that were based on the CycA experiments, and have for example removed reference to these data from our abstract (see also, Results, page 8, paragraph 2, and Discussion, page 12). In addition, we have provided some requested control data, where the effects of CycA alone on pERM levels are now also presented (Fig. 6G).
As described above, we have also included new immunocytochemistry data, which establishes that thrombin induces an increase in pMLC2 (Fig. 1N and 6F), and is not significantly reduced by subsequent Yoda1 addition. Given that increased MLC2 phosphorylation is an established effect of CycA treatment we have not experimentally explored this further here, and while we acknowledge that the conclusions that can be drawn from the CycA experiments are limited, they do establish that PP1/PP2A's are likely involved in the constitutive dephosphorylation or ERMs in MDA-MB-231 cells, while PP3 phosphatases (calcineurin) are not (Fig. 5H and I). Furthermore, addition of Yoda1 to CycA-treated cells did reduce the observed increased in pERM levels, suggesting that Yoda1 could be counteracting the effects of PP1/PP2A inhibition.
Further work will be required to establish a definitive mechanism by which Piezo1 activation is attenuating blebbing, but given the established roles of pERMs in blebbing, and the fact that in thrombin treated cells Yoda1-activation of Piezo1 reduces pERMs back towards untreated control levels (Fig. 6D, E and S7A, B), it is likely that this process is contributing towards bleb attenuation.

4. The relevance of apoptosis-related data is not clear in this manuscript. How does Yoda-1 induced apoptosis help explain the blebbing behavior?

We acknowledge that the apoptosis data does not further our explanation of Yoda1's involvement in the observed blebbing behavior. The original purpose of these experiments was to assess whether the thrombin-induced blebbing was associated with increased apoptosis (as blebbing is also a characteristic of this process). As demonstrated, thrombin stimulation did not increase cell death; however, during these experiments we observed that prolonged exposure to Yoda1 lead to increased apoptosis, and included these findings as supplementary data as we considered they may prove insightful to others (Fig. S4). We have now revised the manner in which this data is discussed, and instead focus on the finding that thrombin exposure is not associated with increased cell death (*Results; Thrombin induces blebbing in MDA-MB-231 cells,* page 3 and 4), and mention the effect of Yoda1 in our Discussion (page 12, paragraph 2).

5. PIEZO1 localization on the plasma membrane is not sufficiently demonstrated. F-actin is not a plasma membrane marker. A definitive plasma membrane marker is required in Figure 5.

We have now revised this figure (now Fig. 4) and have performed new Piezo1 immunocytochemistry (ICC) using WGA to label the plasma membrane. Cells were imaged with confocal microscopy and z-scans with an optical thickness of approximately 1 µm were collected from the first in-focus basal plane, and through to the cells apical surface. A maximum intensity projection of all collected z-planes, and the most basal z-plane only are presented, which illustrates the distribution of Piezo1 throughout this basal layer, but also in the most peripheral edges of the cell. In addition, we include Piezo1 ICC and WGA staining of a thrombin-treated blebbing cell (Fig. S5A). Interestingly, while a lot of the bleb-associated Piezo1 appears to be within the cytosolic volume of the bleb, we also provide evidence of Piezo1 within the bleb membrane. While we continue to include an

© 2022. Published by The Company of Biologists under the terms of the Creative Commons Attribution License (https://creativecommons.org/licenses/by/4.0/).

example of Piezo1 ICC combined with phalloidin staining for F-actin (now Fig. 4B). These images were collected by total internal reflection fluorescence (TIRF) microscopy, where fluorescence emission is collected at a depth of about 100 nm from the point at which the cell is attached to the cover glass, and as such should represent the most peripheral Piezo1 immunostaining that can be detected at the basal plasma membrane of the cell.

6. Vehicle control data are missing in Figure 7F, 7G

Vehicle control data for calyculin A are now included in the figure (now Fig. 6G), in which we demonstrate pERM levels inthe presence and absence of calyculin A diluted in OptiMEM.

7. Is the effect of thrombin, Yoda-1, PIEZO1 etc. conserved in other types of breast cancer cell lines?

While we acknowledge that this will be interesting to pursue we have not yet extended our study to include other breast cancer cell lines. We have now included a comment in the discussion highlighting this (Discussion, page 12, paragraph 2).

8. The author mentioned amoeboid cell state in a couple of places. It would be important to clarify that this manuscript studies blebbing behaviors but not amoeboid cell state, which include blebbing, as well as cell rounding, increased actomyosin contractility, and reduced cell adhesion to ECM. The latter three aspects arenot studied in this work.

We appreciate the reviewer identifying the need for this clarification and we have now revised the text throughout our manuscript to ensure that the distinction between amoeboid cell state and blebbing is more clearly stated. While we agree thatour study predominantly focuses on blebbing behavior, our data demonstrating that the myosin-inhibitor blebbistatin impairs the thrombin-induced blebbing response (now in Fig. 1M), and our new data that indicates a thrombin-mediated increased in myosin phosphorylation (Fig. 1N), both indicate the involvement of increased actomyosin contraction, at least for thrombin- induced blebbing.

Reviewer 2 Advance Summary and Potential Significance to Field:
In this manuscript, O'Callaghan et al. show that contact compression, which increases cytosolic Ca2+, attenuates thrombin-induced blebbing in MDA-MB-231 cells. Similarly, the Piezo1 agonist Yoda1, which induces a sharp increase in cytosolic Ca2+, abolishes thrombin-mediated blebbing. As expected, Piezo1 knockdown impairs the ability of Yoda1 to suppress thrombin-induced blebbing. Interestingly, this molecularintervention moderately exacerbates thrombin-induced blebbing in the absence of Yoda1. Lastly, the authors implicate the PP1A/PPA2 family of serine/threonine phosphatases in Yoda1-mediated reduction of thrombin-induced ERM phosphorylation. Overall, this is a well-executed and interesting albeit phenomenological studyworthy of publication pending an appropriate revision.

Reviewer 2 Comments for the Author:
Comments:
1. The results of this manuscript are in line with recent data showing that increasing hydraulic resistance shifts the cell phenotype from amoeboid/blebbing to mesenchymal in confinement (PMID: 33893091; Zhao et al., Sci. Adv. 2021; 7: eabg4934). As shown in Fig. 7 of that study (PMID: 33893091), TRPM7 isthe key sensor of hydraulic resistance. In view of this finding, the reviewer is wondering whether thrombin-induced cell blebbing is attenuated specifically by activation of Piezo1 or activation of other mechanosensitive ion channels (MICs), such as TRPM7 via the use of Naltriben or TRPV4 via GSK1016790A could also suppress thrombin-induced blebbing.

We have now conducted the suggested experiments using GSK1016790A to stimulate TRPV4 channels, and naltriben to stimulate TRPM7, and the results relating to TRPV4 are presented as Fig. 4J-L and Fig. S6D, and those relating to TRPM7are included as Fig. S6A-C, and E. In addition to the discussion of these new results, we have also specifically cited the above paper by Zhao et al., as well as a related TRPM7 study (mentioned below).

We conclude that neither the TRPV4 or TRPM7 agonists attenuate thrombin-induced blebbing, and so it seemsthat the Piezo1 attenuation of blebbing is at least not a general effect of mechanosensitive ion channel stimulation. In additional combined stimulation experiments, we also

© 2022. Published by The Company of Biologists under the terms of the Creative Commons Attribution License (https://creativecommons.org/licenses/by/4.0/).

demonstrate that both agonists evoked Ca^{2+}-responses in a limited subset of cells, while subsequent Yoda1 addition stimulated a response essentially throughout the entire population. This suggests that MDA-MB-231 cells are perhaps more sensitive to the Piezo1 agonists, than the TRPV4 or TRPM7 activators tested here. Furthermore, the transient Ca^{2+} responses evoked by the TRPV4 and TRPM7 agonists were distinct from the more sustained Ca^{2+} elevation stimulated by Yoda1. We hope the reviewer agrees that these data offer some interesting insights, and evidence that the bleb-attenuation achieved by Piezo1 activation is not a general effect of MIC activation.

2. The authors should verify key findings with an additional cell line or at least acknowledge the use of a single cell line (MDA-MB-231) as a limitation.

While we agree that it would be interesting to assess our findings in additional cells lines, we have not yet pursued this line of investigation. We acknowledge that this is an important limitation of our study, and as advised we have now highlighted this fact in our Discussion (page 12, paragraph 2)

3. Line 144: “spontaneous blebbing could be attenuated following initial gentle contact compression”. Inclusion of quantitative data would strengthen their manuscript.

We have now included quantitative data outlining the effects of gentle contact compression on spontaneous blebbing. This is presented as Fig. 2E.

4. Comparing Fig. 4F to Fig. 2C, it is evident that there is data variability. How do the authors explain this? Is this due to different cell passage numbers used in different experiments?

We have considered possible explanations for this observed variability, and as suggested by the reviewer cell passage number may play a role in these differences. We have also considered whether it may be influenced by the specific degree of thrombin activity in a given sample aliquot. We have ruled out potential effects from the attachment substrate, as we have observed similar results whether we use cover-glass dishes from IBIDI or Mattek. While we cannot provide a definitive explanation for this variability, we observe that the relative effects of the described treatments are consistent throughout. We have added a short description of this variability in our Discussion (page 12, paragraph 2).

5. They only show Piezo 1 mRNA expression of siControl and siPiezo1 specimens. A western blot is needed to confirm knockdown at the protein level.

We have attempted to perform Western blots for Piezo1 and unfortunately have not succeeded in obtaining a specific signal. While we appreciate that a Western blot is necessary to analyze the degree of knockdown at the protein level, we suggest that our experiments demonstrating an impaired Ca^{2+} response to Yoda1 in siPz1-treated cells compared to siCtrl-treated cells (Fig. 5B, C) provides strong loss-of-function evidence, indicating that our knockdown protocol is effective.

6. In Fig. 7F-G, data showing the effect of CycA on ERM phosphorylation would be helpful to the reader. The demonstration of the involvement of PP1/PP2As in this process is indirect, and the authors should soften their wording.

We have now included an additional Western blot demonstrating the effect of CycA alone on ERM phosphorylation (Fig. 6G), and discussed this result accordingly. In addition, we have now flipped the order of the Western blot and accompanying analysis in Fig. 6H and I, so that the thrombin-treated results are presented before the results from cells subsequently treated with Yoda1. We consider that this is a more logical order to present this data. We appreciate the reviewer’s comments regarding the degree of evidence supporting the involvement of PP1/PP2A, and as advised have now reworded the text in key sections of the manuscript. For example, we have now removed reference to PP1/PP2As from the abstract, and in the Results section, we now suggest that our data supports a role for PP1/PP2As in the constitutive dephosphorylation of ERMs (as indicated by the new panel Fig. 6G), and that their inhibition is sufficient to induce blebbing (Fig. S8). Further, the increase in pERM levels in thrombin and CycA-treated cells is reduced by addition of Yoda1, so Yoda1-activation of Piezo1 does seem to stimulate a response that counteracts the inhibited dephosphorylation of pERMs caused by CycA. Please see new text in Results, page 8,

© 2022. Published by The Company of Biologists under the terms of the Creative Commons Attribution License (https://creativecommons.org/licenses/by/4.0/).

paragraph 2, and Discussion, page 12, paragraph 2.

7. Statistical analysis: The authors should ensure the normal distribution of their data before performing the Student's t-test or one-way ANOVA. For instance, are the data in Fig. 2L normally distributed?

We have revised the statistical analyses performed in the paper and selected appropriate alternative tests for datasets which were not normally distributed. We have rewritten our Materials and Methods accordingly.

8. The authors should cite two additional articles which are directly relevant to this manuscript. First, PMID: 32789173 shows that confinement promotes cell blebbing. Second, PMID: 31355337 shows that loss of cortical actin increases the sensitivity of MICs.

We thank the reviewer for drawing these interesting studies to our attention, and we have now added these citations to our revised discussion.

Reviewer 3 Advance Summary and Potential Significance to Field:
The work by O'Callaghan et al. addresses the regulation of thrombin induced blebbing by Piezo1 in MDA MB-231 breast cancer cells. Using a custom-made "cell press" the authors describe the intriguing observation that gentle cell confinement (termed contact compression) leads to an inactivation of both spontaneous and thrombin-induced cell blebbing, whereas strong cell compression in the absence of thrombin induces pronounced cell blebbing as previously described in other cell types. The authors show that contact compression was accompanied with an increase of intracellular calcium levels which led them to study the involvement of the mechano-sensitive ion channel Piezo1 in thrombin-induced cell blebbing. Using the Piezo1 agonist Yoda1 and siRNA interference the authors identify a role of Piezo1 in modulating thrombin-induced cell blebbing. They propose that this process is regulated by PP1A/PP2A phosphatases upon Piezo1 activation by reducing thrombin-mediated ERM protein phosphorylation.

The article is clearly written and the data figures are presented in a detailed and consistent format easing the reading of the manuscript. The finding that Piezo1 activation inhibits thrombin-induced cell blebbing observation is interesting, however whether Piezo1 is involved in the mechanical cell response to contact compression remains unclear. It appears surprising that the authors have not tested the role of Piezo1 in contact compression to see if blebbing persists when reducing Piezo1 activity by the described siRNA approach and how this would link to calcium levels upon contact compression. Furthermore, the authors should clarify the following key points below related to the role of intracellular calcium levels and the function of PP1A/PP2A in regulating cell blebbing.

Reviewer 3 Comments for the Author:
- The authors argue that thrombin-induced calcium release from the ER stimulates cell blebbing. Contrary, a Piezo-induced calcium increase inhibits cell blebbing. This raises the question how intracellular calcium levels in the studied system regulate two opposing cell responses, activation of blebbing and inhibition of blebbing.

As mentioned above, we acknowledge that the opposing effects of increased cytosolic Ca^{2+} with regard to bleb induction, and bleb attenuation were not explicitly addressed in our original manuscript, and we have now added a more extensive discussion (page 12, paragraph 2 in the Discussion, which starts *"Notably, here we report elevated cytosolic* Ca^{2+}*...."*. In this regard, we discuss the fact that the Ca^{2+} response to thrombin is transient and short-lived, while typically a more sustained Ca^{2+} elevation is observed for Yoda1-induced Piezo1 activation, and contact compression, and cite two important studies (Dolmetsch et al., 1997, Timmerman et al., 1996) where they describe how the capacity for the cell to distinguish between different amplitudes and durations of Ca^{2+} responses, permits different outcomes.

Is the calcium increase upon thrombin treatment necessary for cell blebbing or would an inhibition of the thrombin-induced calcium increase also promote cell blebbing?

This is an interesting question and we believe that some of the data already presented may offer some insights. Thrombin- induced blebbing is impaired in cells pretreated with the myosin inhibitor

© 2022. Published by The Company of Biologists under the terms of the Creative Commons Attribution License (https://creativecommons.org/licenses/by/4.0/).

para-aminoblebbistatin (Fig. 1M). However, thrombin still induces an increase in cytosolic Ca^{2+} in these cells (Fig. S3F), confirming that the Ca^{2+} response is only associated with blebbing in myosin-competent cells, offering support for our proposal that thrombin-induced Ca^{2+} is contributing to blebbing by potentiating the activity of myosin. Cells engaging in spontaneous blebbing and cells blebbing inresponse to CycA do not exhibit elevated cytosolic Ca^{2+} levels, indicating that the basal levels of actomyosin contractility, and/or the integrity of actin-PM contacts alone may influence a cells propensity to bleb independently of a change in cytosolic Ca^{2+}. Considering that thrombin induces PIP2 hydrolysis (which has to precede IP3-mediated Ca^{2+} release from theER), and in doing so reduces actin-PM contacts, which Welf et al. and others have established can initiate blebbing (Welf et al., 2020), it is reasonable to assume that thrombin would trigger a degree of blebbing even in the absence of the subsequent potentiating effect Ca^{2+} has on actomyosin contractility.

Finally, in our revision we have assessed the capacity of TRPV4 and TRPM7 activation by agonists to attenuatethrombin-induced blebbing (Fig. 4J-L, and Fig. S6A-E), and concluded that in this context these channels do not share this function with Piezo1. Interestingly, TRPV4 activation in some cells resulted in a Ca^{2+} response with a similarly transient profile as that observed for thrombin (Fig. S6D), but this did not lead to induction of blebbing. Consequently, while the thrombin-induced Ca^{2+} response plays an established role in promoting actomyosin contractility, there are additional upstream events that are required to increase a cells propensity to bleb, and similar Ca^{2+} responses produced by an influx of Ca^{2+} through e.g. TRPV4 are insufficient to cause blebbing.

Would an increase of intracellular calcium levels after thrombin addition or contact confinement by alternative strategies(using for example Thapsigargin or Ionomycin) also lead to an inhibition of blebbing?

We have performed experiments with thapsigargin and include the results here for the reviewer's consideration, but as our observations with respect to blebbing were inconclusive we have not included this data in the manuscript. In brief, thapsigargin treatment resulted in a gradual and sustained elevation of cytosolic Ca^{2+}, resulting from store operated Ca^{2+} entry, due to inhibition of ER refilling. This mechanism has previously been described for these cells (Motiani et al., 2010). As cytosolic Ca^{2+} increased in thapsigargin-treated cells we observed limited examples of both bleb induction and bleb attenuation. However, after thapsigargin treatment we found that subsequent addition of Yoda1 resulted in a drop of cytosolicCa^{2+}, which gradually recovered to the new elevated baseline established after thapsigargin addition (Response Fig. 1) Therefore, due to this altered and apparently inhibited Piezo1 activity in thapsigargin treated cells it was not possible to assess the capacity of Yoda1 to alter blebbing behavior.

We would also refer the reviewer to our discussion above in which we describe our new data using agonists against the mechanosensitive ion channels TRPV4 and TRPM7. Briefly, cells that responded to TRPV4 presented with a Ca^{2+} response profile similar to that obtained for thrombin (and the PAR agonists), namely a transient short-lived increase. However, TRPV4 activation following thrombin neither increased the proportion of blebbing cells, nor was it capable of attenuating blebbing (Fig. 4J-L). Similar results were obtained with a TRPM7 agonist (Fig. S6A). We hope the reviewer finds that these additional data are informative in this regard.

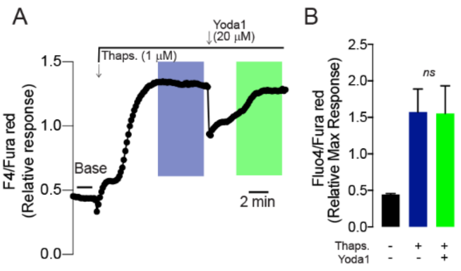

Response Fig. 1. Ca^{2+} response to thapsigargin and subsequent Yoda1 treatment in MDA-MB-231 cells.

Can the authors explain what leads to a gradual increase of intracellular calcium levels upon contact compression whilePiezo1 activation triggers a transient calcium change?

© 2022. Published by The Company of Biologists under the terms of the Creative Commons Attribution License (https://creativecommons.org/licenses/by/4.0/).

In contrast to Yoda1, which specifically targets Piezo1, contact compression will likely activate multiple stretch/touch activated channel types. It is conceivable that different channel types may contribute to elevated Ca^{2+} in a delayed fashion, and/or that co-operation between multiple different touch sensitive channels may give rise to the observed increase. In this regard we also provide new data demonstrating the effect that TRPV4 and TRPM7 agonists have on cells subsequently co- stimulated with Yoda1 (Fig. S6D and E). Interestingly, the TRPV4 agonist appears to support a more sustained elevation of Yoda1-induced cytosolic Ca^{2+}, suggesting a co-operative interaction between these channels. Nonetheless, both Yoda1 and contact compression induce a rapid elevation of cytosolic Ca^{2+}, and while Yoda1 levels do start to decrease, they are sustained considerably higher than baseline for the duration of any of the experiments conducted here (Fig. 4G, 5B, 6A, S5C, E), while the thrombin stimulated Ca^{2+} response profile is always short-lived and transient, and Ca^{2+} levels predominantly return to baseline.

The authors could thereby strengthen the arguments on the suggested involvement of the mechano-sensitive activity of Piezo1 in regulating cell responses to compression, which would benefit to present the work more consistently. An involvement of Piezo1 in the cell blebbing response to larger deformation would further be interesting to assess.

We have undertaken these experiments, but must unfortunately report that our knock-down of Piezo1 is insufficient to establish an impaired Ca^{2+} response to contact compression. We include a short discussion and summary of this data here for the reviewer's information (Response Fig. 2).

Three independent siCt- and siPz1-transfections were performed in duplicate. To confirm that a functional Piezo1 knock-down was achieved one dish of each condition was exposed to Yoda1, and as before we consistently detected a significant reduction in Ca^{2+} responses for siPz1-treated cells compared to siCtrls (One example is presented in Response Fig. 2A). However, when we subsequently performed thrombin treatment followed by contact compression, we found that the Ca^{2+} response to compression was similar in both siPz1 and siCtrl conditions (Response Fig. 2B). A possible explanation for this effect is that unlike Yoda1, which specifically activates Piezo1, contact compression will activate a wider range of mechanosensitive channels, and that through compensatory effects the net Ca^{2+} influx will be similar between siCt and siPz1 cells. Our new data using the TRPV4 and TRPM7 agonists indicate that activation of these channels is not sufficient to attenuate thrombin-induced blebbing, but contact-compression may activate other channels not explored here, or activate co-operative effects between multiple different channels (discussed above), such that the functional loss of Piezo1 from this system is absorbed by other channels. During this study we have attempted to transfect cells with higher concentrations of siRNA, but we then experienced issues with cell viability.

Therefore, while our data using Yoda1 and Piezo1 knockdown (Fig. 5) supports our conclusion that Piezo1 is a contributor to the bleb attenuation response, other channels may also be recruited during the contact-mediated attenuation (Fig. 3). Given this limitation it has also not been possible to assess the role of Piezo1 in the blebbing response to deformation; although, we do mention on page 11, paragraph 1 the fact that Lomakin et al. (Lomakin et al., 2020) conclude that Piezo1 inhibition via GsMTx4 does not impair the capacity of melanoma cells to migrate, suggesting that the mechanoresponse that induces blebbing following nuclear deformation, involves mechanisms that are distinct from the bleb attenuation that follows contact compression.

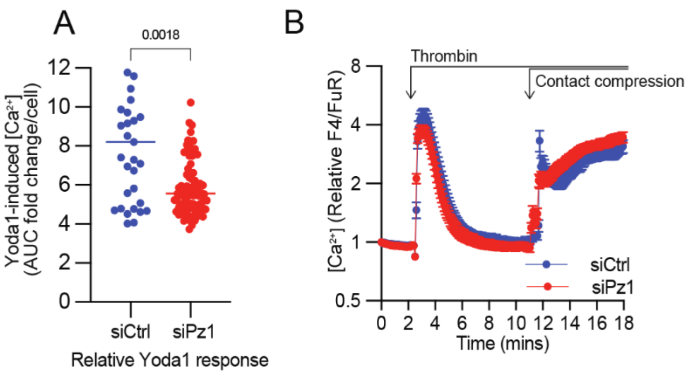

Response Fig. 2. Ca^{2+} response to Yoda1 (A), and thrombin followed by contact compression (B) in siCtrl and siPz1 treated cells.

© 2022. Published by The Company of Biologists under the terms of the Creative Commons Attribution License (https://creativecommons.org/licenses/by/4.0/).

- The authors suggest a role of PP1A/PP2A in controlling cell blebbing via phosphorylation of ERM proteins. As the authors point out, CalyculinA is also causing changes in the phosphorylation of other proteins and most notably Myosin 2 proteins.

It remains however unclear how Myosin 2 activity is affected by the treatments described in the paper. To which extend do thrombin and contact compression affect phosphorylation of Myosin 2 and how do these levels vary depending on the activation/inhibition of intracellular calcium levels?

We have now addressed this issue and included immunocytochemistry data and image analysis of pMLC2 levels before and after thrombin treatment, with or without the subsequent exposure to Yoda1 (Fig. 1N and 6F). We demonstrate that thrombin induces an increase in pMLC2, which is not significantly suppressed by Yoda1. Therefore, while Yoda1- mediated Piezo1 activation reduced pERM levels (Fig. 6D, S7A-E), it did not affect pMLC2 levels. We have also reworded our results and discussion text, such that we present our claims relating to PP1/PP2A phosphatases as potential Piezo1 effectors more cautiously, and have for example removed reference to these findings from our abstract. Please read the revised text in the Results, page 8, paragraph 2, and Discussion, page 12, paragraph 2.

Would also a non-specific increase of intracellular calcium lead to pERM increase? And does siRNA reduction of Piezo1 maintain ERM phosphorylation upon thrombin and contact compression?

Several kinases have been implicated in ERM phosphorylation, including Rho-associated protein kinase, NFkB-inducing kinase, and protein kinase C (reviewed in (Ponuwei, 2016). As thrombin mediate PAR activation, will lead to protein kinase C activation, through diacylglycerol exposure at the PM (following PLC-mediated PIP2 hydrolysis), and Ca^{2+}-release from the ER, it seems a likely kinase candidate for the thrombin-mediated increase in ERM phosphorylation observed here. However, as other kinases, which are not Ca^{2+}-dependent can also induce ERM phosphorylation, the potential role of Ca^{2+} will be context dependent. For example, in the paper we also demonstrate that PP1/PP2A inhibition with CycA is sufficient to increase pERM levels (Fig. 6G), but this occurs independently of an intracellular Ca^{2+} increase (Fig. S8). Equally, an increase in intracellular Ca^{2+} without a concomitant increase in DAG availability, will be unlikely to lead an increase in pERMs. This is in part supported by the fact that Yoda1 treatment (before or after thrombin addition), which also increases intracellular Ca^{2+}, does not increase, but rather reduces pERM levels (Fig. 6D, and Fig. S7A). Therefore, we do not consider that any mechanism that increases intracellular Ca^{2+} will be sufficient to induce an increase in pERMs. As discussed above, given the limitations we experienced with our siPiezo1 treatment combined with contact compression experiments, we have not been able to assess a potential effect of Piezo1 knockdown on pERM levels following contact compression.

- The "cell press" confinement device is indicated to perform cell compression in 5µm steps during the first cell contact compression. This value appears very coarse to define it as a contact-compression. What is the standard cell height and nucleus height? This would allow to estimate the average cell deformation when a calcium influx is observed.

We thank the reviewer for addressing this important point. We have now assessed the cell and nucleus height from images of fixed and immunostained cells, and have included some examples below for the purpose of discussion. As described in the Materials and Methods we move the ‘cell press’ downwards in 5 µm steps until we observe a Ca^{2+} response, we typically begin taking 5 µm steps when approximately 100 µm from the coverglass, the position of which is calibrated prior to the start of the experiment. In Response Fig. 3 we present the xz planes for the ‘tallest’ acquired position for 6 cells that were stained with WGA (plasma membrane) and NucBlue. The cells measured 16.9, 15.1, 14.3, 13.2, 11.0 and 10.3 µm in height.
Consequently, if the cell press was 20 µm from the cover-glass, the next 5 µm step (to 15 µm) would contact the two tallest cells, and a subsequent 5 µm step would ensure contact with the remaining cells. At this point all cells would be compressed to a height of 10 µm, and during this contact compression we do not observe nuclear deformation (Fig. 2J and K). Lomakin et al. observe a contractile cellular response when cells are compressed to a height of 5 µm, which is not observed when cells are compressed to a height of 20 µm, or 10 µm, and they attribute this contractile response to a mechanism that is dependent on nuclear membrane expansion (Lomakin et al., 2020). With 5 µm steps we found that we can stimulate a coordinated contact-mediated Ca^{2+}

© 2022. Published by The Company of Biologists under the terms of the Creative Commons Attribution License (https://creativecommons.org/licenses/by/4.0/).

response in a large field of cells of differing heights. We can subsequently compress cells to the point of deformation by further lowering the cell press in 2 µm steps until we visually observe an increase in the projected nuclear area, and see bleb formation. Measures of the nucleus height are complicated by the fact that we have observed that contact compression can result in nucleus repositioning, sometimes moving it to a lower position within the cell. This relocation does not seem to result in blebbing as it does not visibly squeeze the nucleus, but it makes it difficult to measure the projected nuclear area from live imaging data as we typically only collect one z-plane to ensure sufficiently fast image acquisition.
During the revision we used WGA to stain the plasma membrane in fixed cells, and while the dye is compatible with live imaging, in the context of tunneling nanotube studies it has been reported to increase stiffness of the PM, and we have not assessed its potential effect on bleb dynamics during live imaging. We acknowledge that determining what degree of displacement elicits the contact-induced Ca^{2+} response would be interesting to elucidate, but given the variability in cell heights, for the purpose of the wide-field of view experiments we have conducted it is more useful to compress cells using the admittedly coarser 5 µm steps to ensure a robust coordinated contact response, while still not exceeding the compression threshold required for the 'nuclear ruler' response.

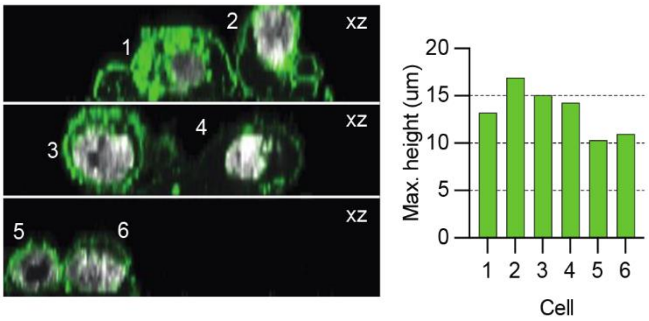

Response Fig. 3. Examples of cell heights as measured in fixed WGA-stained MDA-MB-231 cells. Images presented along the xz-axis for 6 cells that were treated with thrombin.

The authors could potentially also use the nucleus area change as an additional parameter to calcium influx to assess cell contact compression more accurately. Furthermore, is the measured force dependent on the number of cells under the PDMS pillar or the activation of a contractile cell response upon compression?

We agree that conducting nuclear deformation measurements in addition to Ca^{2+} influx throughout the study would in general have provided us with more information for assessing the contact compression responses. However, as this data is included in Fig. 2 to investigate and define the different responses to compression (contact vs. deformation), we suggest that our approach in Fig. 3 where blebbing is assessed before and after a contact-induced Ca^{2+}-response is still a reliable protocol for this analysis. In addition, for the purpose of the analyses in Fig. 3 compression was stopped after we observed the initial Ca^{2+} response before nuclear deformation is induced, so as such no changes in nuclear area are expected.
As the reviewer indicates, we consider that the measured force will be influenced by the number of cells under the pillar, but the purpose of these measurements is to assess relative changes in the force exerted on a given cell population following increased downward displacement of the pillar. In principle, as the compression pillar is in direct contact with the donut load sensor and its surface is in direct contact with cells, it is conceivable that contractile cells could exert a repulsive force on the pillar, which would upwardly deflect the pillar and reduce the perceived load. However, while such measurements can be reliably performed with e.g. microcantilever-based devices, given the size of the PDMS pillar, it seems unlikely that the 'cell press' can detect such deflective forces.

And was the PDMS functionalized or passivated and do the authors expect an influence of the PDMS material properties on the measured cell response?

© 2022. Published by The Company of Biologists under the terms of the Creative Commons Attribution License (https://creativecommons.org/licenses/by/4.0/).

The PDMS pillar used in this study is not functionalized; for example, we do not plasma treat the PDMS which is common when the goal is to bind PDMS-based devices to glass surfaces, as PDMS is a hydrophobic material and cells do not readily attach to it. This hydrophobic property is evident in experiments where we release cells following contact and/or deformation, and the cells remain attached to the underlying coverslip and do not get 'stretched' upwards as we move the pillar away. We have now added a sentence to our Materials and Methods, clarifying this property of the PDMS pillar (Page 16, paragraph 2).

Minor points:

- How do the authors explain that the attenuation of blebbing persisted after contact compression was released (Fig. 4E, and Movie 3)?

One possible explanation for this effect is that the sustained period of contact prior to release is sufficient to promote and stabilize new contacts between the actin cortex and the PM; therefore, upon release the cells blebbing propensity is already reduced.

Do cells upon release respond to thrombin?

During some earlier work on this study we treated cells with the PAR2 agonist after contact compression and observed examples of cells which no longer exhibited a Ca^{2+} response, and we consider that the thrombin response would be similarly muted after contact compression. This in line with the results obtained when cells are pretreated with Yoda1, and when subsequently treated with thrombin we observe an impaired Ca^{2+} response and we do not see an induction of blebbing (Fig. 6A-C).

Does mild contact (and its release) lead to cell apoptosis?

We have not specifically analyzed this possibility, although others have confirmed that sustained and greater degrees of compression are not associated with cell death (e.g. (Lomakin et al., 2020)). Furthermore, as detailed in the paper, cells are still capable of Ca^{2+} responses after cycles of mechanostimulation (Fig. 2G, and H-J), indicating that during the time-course of the experiments their sensitivity to these signals are intact.

- Thrombin addition leads to a rapid and transient calcium increase but apparently the blebbing response is delayed which would be interesting to comment.

We have now added a comment in the Discussion section of the paper about this (please see page 10, paragraph 1).

- Do the two populations of spontaneously blebbing/non-blebbing cells behave differently upon thrombin treatment and contact compression?

We now include data in Fig. 2E which demonstrates that spontaneous blebbing is attenuated by contact compression. In contrast, non-blebbing cells were only induced to form blebs when compressed to the point that their nucleus was deformed (Fig. 2D), while contact compression (where the nucleus was not deformed (Fig. 2J, K)) was not observed to induce blebbing.

In the majority of cases spontaneously blebbing cells continue to bleb after the addition of thrombin, we have not included this specific analysis in the paper, but as an example we reviewed the bleb status of the cells in the siCtrl populations included in Fig. 5H. and observed that of the 55 spontaneously blebbing cells, 54 (98%) continued to bleb after the addition of thrombin.

- Is there a possible involvement of Piezo2 or other mechano-sensitive ion channels in the studied cell type as identified in different migratory cell types? A comment would be relevant to include.

We have now included qPCR data in which we analyze Piezo1 and Piezo2 transcript levels, and demonstrate that Piezo1 is the major Piezo isoform in this cell-type (Fig. S5B). In addition, we have now studied the capacity of TRPV4 and TRPM7 channel agonists to attenuate thrombin-induced

© 2022. Published by The Company of Biologists under the terms of the Creative Commons Attribution License (https://creativecommons.org/licenses/by/4.0/).

blebbing, and included an analysis of the Ca^{2+}-responses stimulated by these agonists in comparison to those induced by Yoda1. These analyses are included as Fig. 4J-L, and Fig. S6A-E. We discuss this new data and cite relevant publications recommended by this reviewer and other reviewers.
Fig. 1C seems to show the wrong time axis when compared to Fig. 1B.

We thank the reviewer for identifying this error, which we have corrected in the revised version (Fig. S1)

- Fig. 2M shows a fraction of blebbing cells in the presence of blebbistatin and thrombin, how do the authors explain the occurrence of this blebbing cell fraction?

We were also initially surprised that thrombin still induced blebbing in a subpopulation of para-aminoblebbistatin-treated cells. However, thrombin-induced hydrolysis of PIP2 will still cause dissociation of actin-PM contacts (including contacts maintained by basal pERM levels) even in these myosin-inhibited cells, and as described by Welf et al. this loss of actin-PM contact can be sufficient to induce bleb formation (Welf et al., 2020). However, blebs in these cells are also retracted, which does imply actomyosin activity. Blebbistatin inhibits myosin by preferentially binding to its (ADP + phosphate (Pi))-bound state, slowing down Pi release. However, blebbistatin does not affect myosin's ability to bind actin (i.e. in its nucleotide-free state) or the capacity of ATP to bind myosin and dissociate it from actin (Kovács et al., 2004) i.e. its potential to contract actin is impeded, not abolished. In contrast, Ca^{2+} activated calmodulin enhances MLCK activity and thus MLC2 phosphorylation, which is in turn associated with enhanced myosin ATPase activity i.e. increasing the rate of ATP hydrolysis to (ADP + Pi). Therefore, it is conceivable that the positive effect of thrombin-induced Ca^{2+} on myosin ATPase activity, may partially compensate for the inhibitory effect blebbistatin has on myosin Pi-release, and could perhaps explain the limited increase in myosin activity observed in these cells.

- Fig. 2N at which time points was ERM phosphorylation measured and does it persist over time?

In this panel (now presented in Fig. 2O) the pERM levels are measured after 5 mins of thrombin treatment. Others have described how the pERM levels are reduced over time; for example, Adysehv et al. demonstrate how maximum thrombin- induced pERM levels are observed after 5 min followed by a gradual time-dependent reduction, but are still higher than baseline after 2 h (Adyshev et al., 2013), and we cite this study in our manuscript. In Fig. S7A, B, D and E cells were treated with or without thrombin for 5 min, after which either Yoda1 or OptiMEM (Ctrl) was added for a further 10 mins (in the ICC figures) or 15 mins (in the Western data). Therefore, these data represent pERM levels 15-20 mins after thrombin stimulation.

- Fig. 3E spontaneous blebbing does not seem to correlate with a calcium increase, is this consistently observed and how to the authors argue about the occurrence of spontaneous blebbing?

This is something that we have also considered during this study, and is relevant to many of the other model cell systems that have been used in studies of bleb dynamics. As discussed (page 9, paragraph 2), we suggest that the deletion of Merlin form MDA-MB-231 cells, an ERM-like protein, likely contributes to an increased risk for blebbing (Morrow et al., 2011, Shapiro et al., 2014). Which is in line with other studies in where the integrity of actin-PM contacts, mediated by other cross-linkers, and actin-binding proteins underlie the propensity with which a cell is likely to engage in blebbing behavior. As observed by the reviewer, we do not observe changes in cytosolic Ca^{2+} in spontaneously blebbing MDA-MB-231 cells; therefore, it is possible that cells engaging in this spontaneous blebbing behavior have sufficiently high levels of resting actomyosin contractility, combined with sufficiently low degrees of actin-PM contacts, which tip the balance in favor of a blebbing phenotype. Equally, thrombin-induced changes such as reduced actin-PM contacts (through PIP2 hydrolysis), and increased actomyosin contractility (through a Ca^{2+}-mediated increase in myosin activity), will also tip the balance in favor of blebbing. A further insight into this propensity is afforded by our Piezo1 knockdown experiments, in which we observed that thrombin-induced blebbing was enhanced in siPiezo1-treated cells, which suggests that Piezo1 activity in a cell, as regulated by local actomyosin traction forces (Ellefsen et al., 2019), may also contribute to reducing the likelihood of a cell adopting bleb behavior.

© 2022. Published by The Company of Biologists under the terms of the Creative Commons Attribution License (https://creativecommons.org/licenses/by/4.0/).

- The thrombin-induced calcium spikes in Fig. 5G are consistent with Fig. 2D but not 4A, how do the authors explain the difference?

The thrombin-induced Ca^{2+}-response, presented now in Fig. 6A, which deviates from the others presented in the study, is observed after we first stimulate cells with Yoda1. Interestingly, thrombin first elicits a decrease in the levels of cytosolic Ca^{2+} that were induced by Yoda1, after which a transient, but slower and less pronounced Ca^{2+} increase is recorded. We do not have a definitive explanation for this effect, but have considered the following possibilities: Piezo1 activity is dependent on PIP2 (Borbiro et al., 2015), and thrombin promotes PIP2 hydrolysis, which could result in Piezo1 inactivation and potentially explain the observed initial drop in cytosolic Ca^{2+}. In other experiments where cells are treated first with thrombin and then Yoda1, the delay between thrombin and Yoda1 treatment should be sufficient to permit PIP2 resynthesis at the PM, and therefore it is not then observed to be a limiting factor for Piezo1 activation. For example, Falkenburger et al. determine that following phospholipase C-mediated PIP2 depletion (via muscarinic receptor activation), PIP2 is resynthesized within approximately 2 mins (Falkenburger et al., 2010), and the minimum delay between thrombin and Yoda1 treatments in our study is typically 5 mins. The delayed and reduced Ca^{2+} response to thrombin may reflect a depletion of ER stored Ca^{2+}, as it is possible that Ca^{2+} influx via Piezo1 may also induce a ryanodine receptor-mediated release of Ca^{2+} from the ER, thus producing the observed alterations to the thrombin-mediated Ca^{2+} response.

Fig. 4F data for contact compression without thrombin should be included

We have now included a quantification for contact-attenuation of spontaneous blebbing, presented in Fig. 2E.

- Fig. 6B x scale is missing

We thank the reviewer for identifying this error, we have not added this.

- Fig. 7A, what causes the faster decay upon Yoda treatment in this experiment which appears inconsistent with the decay in Fig. 6B?

We have also observed differences in the rate of cytosolic Ca^{2+} reduction following Yoda1 stimulation in individual cells, and some example traces from individual cells are included as Fig. 4H, where differences in response profiles can be appreciated. We have observed examples of oscillatory responses, and cells whose levels appear to gradually increase after the initial spike, while others continually decay. As the Yoda1-stimulated traces presented in Fig. 4G, 5B, 6A and S5C are averaged plots for a relatively larger number of cells, it is likely that these apparent differences in decay rates perhaps reflect variability with respect to the ratios of the different types of Ca^{2+} profiles that manifest after the initial spike is evoked.
Importantly, despite these differences, the Yoda1-mediated increase in cytosolic Ca^{2+} is sustained clearly above baseline in the vast majority of cells for the duration of these experiments (also mentioned above).

- Several main figure panels could be moved to the supplementary information to reduce the number of main figures as they often show supporting information (including for example Fig. 1 panels, Fig. 3J which appears redundant to panel 3C and Fig. 6H, I).

As suggested we have now moved the previous Fig. 1 to the supplementary information (now Fig. S1).
Panel 3C and 3J: While we agree there are similarities, we consider that the differences between these experiments warrant both panels being presented, which are now presented in Fig. 2C and 2K. Specifically, in Fig. 2C we detail how compression that leads to nuclear deformation induces bleb formation, while in Fig. 2J we demonstrate that the capacity of contact compression to attenuate blebbing in a spontaneously blebbing cell, can still be reversed by deformation. Equally, the previous panels Fig. 6H and I, now presented as Fig. 5H and I, are also included to illustrate the specific and relative differences between the bleb-inducing effect of thrombin (Fig. 5H) and the attenuating effect of Yoda1 (Fig. 51) in siCtrl and siPz1 treated cells.

© 2022. Published by The Company of Biologists under the terms of the Creative Commons Attribution License (https://creativecommons.org/licenses/by/4.0/).

References

Adyshev, D. M., Dudek, S. M., Moldobaeva, N., Kim, K.-m., Ma, S.-F., Kasa, A., Garcia, J. G. N. & Verin, A. D. (2013). Ezrin/radixin/moesin proteins differentially regulate endothelial hyperpermeability after thrombin. *American journal of physiology. Lung cellular and molecular physiology,* **305,** L240-L255.

Borbiro, I., Badheka, D. & Rohacs, T. (2015). Activation of TRPV1 channels inhibits mechanosensitive Piezo channel activity by depleting membrane phosphoinositides. *Science Signaling,* **8,** ra15-ra15.

Dolmetsch, R. E., Lewis, R. S., Goodnow, C. C. & Healy, J. I. (1997). Differential activation of transcription factors induced by Ca2+ response amplitude and duration. *Nature,* **386,** 855-8.

Ellefsen, K. L., Holt, J. R., Chang, A. C., Nourse, J. L., Arulmoli, J., Mekhdjian, A. H., Abuwarda, H., Tombola, F., Flanagan, L. A., Dunn, A. R., et al. (2019). Myosin-II mediated traction forces evoke localized Piezo1- dependent Ca2+ flickers. *Communications Biology,* **2,** 298.

Falkenburger, B. H., Jensen, J. B. & Hille, B. (2010). Kinetics of PIP2 metabolism and KCNQ2/3 channel regulation studied with a voltage-sensitive phosphatase in living cells. *Journal of General Physiology,* **135,** 99-114.

Kovács, M., Tóth, J., Hetényi, C., Málnási-Csizmadia, A. & Sellers, J. R. (2004). Mechanism of blebbistatin inhibition of myosin II. *J Biol Chem,* **279,** 35557-63.

Lomakin, A. J., Cattin, C. J., Cuvelier, D., Alraies, Z., Molina, M., Nader, G. P. F., Srivastava, N., Sáez, P. J., Garcia-Arcos, J. M., Zhitnyak, I. Y., et al. (2020). The nucleus acts as a ruler tailoring cell responses to spatial constraints. *Science,* **370,** eaba2894.

Morrow, K. A., Das, S., Metge, B. J., Ye, K., Mulekar, M. S., Tucker, J. A., Samant, R. S. & Shevde, L. A. (2011). Loss of tumor suppressor Merlin in advanced breast cancer is due to post-translational regulation. *The Journal of biological chemistry,* **286,** 40376-40385.

Motiani, R. K., Abdullaev, I. F. & Trebak, M. (2010). A Novel Native Store-operated Calcium Channel Encoded by Orai3: SELECTIVE REQUIREMENT OF Orai3 VERSUS Orai1 IN ESTROGEN RECEPTOR-POSITIVE VERSUS ESTROGEN RECEPTOR-NEGATIVE BREAST CANCER CELLS*. *Journal of Biological Chemistry,* **285,** 19173-19183.

Ponuwei, G. A. (2016). A glimpse of the ERM proteins. *Journal of Biomedical Science,* **23,** 35.

Shapiro, I. M., Kolev, V. N., Vidal, C. M., Kadariya, Y., Ring, J. E., Wright, Q., Weaver, D. T., Menges, C., Padval, M., Mcclatchey, A. I., et al. (2014). Merlin deficiency predicts FAK inhibitor sensitivity: a synthetic lethal relationship. *Science translational medicine,* **6,** 237ra68-237ra68.

Timmerman, L. A., Clipstone, N. A., Ho, S. N., Northrop, J. P. & Crabtree, G. R. (1996). Rapid shuttling of NF-AT in discrimination of Ca2+ signals and immunosuppression. *Nature,* **383,** 837-840.

Vanderboor, C. M., Thibeault, P. E., Nixon, K. C., Gros, R., Kramer, J. M. & Ramachandran, R. (2020). Proteinase-Activated Receptor 4 (PAR4) Activation Triggers Cell Membrane Blebbing through RhoA and ß-arrestin. *Molecular Pharmacology,* mol.119.118232.

Welf, E. S., Miles, C. E., Huh, J., Sapoznik, E., Chi, J., Driscoll, M. K., Isogai, T., Noh, J., Weems, A. D., Pohlkamp, T., et al. (2020). Actin-Membrane Release Initiates Cell Protrusions. *Developmental Cell,* **55,** 723-736.e8.

Second decision letter

MS ID#: JOCES/2021/258809

MS TITLE: Piezo1 activation attenuates thrombin-induced blebbing in breast cancer cells

AUTHORS: Paul O'Callaghan, Adam Engberg, Olle Eriksson, Nikos Fatsis-Kavalopoulos, Christina Stelzl, Gonzalo Sanchez, Olof Idevall-Hagren, and Johan Kreuger
ARTICLE TYPE: Research Article

© 2022. Published by The Company of Biologists under the terms of the Creative Commons Attribution License (https://creativecommons.org/licenses/by/4.0/).

We have now reached a decision on the above manuscript.

To see the reviewers' reports and a copy of this decision letter, please go to: https://submit-jcs.biologists.org and click on the 'Manuscripts with Decisions' queue in the Author Area. (Corresponding author only has access to reviews.)

As you will see, the reviewers gave generally favourable reports but reviewer 3 raised some final points that need clarification. As the reviewer noted, "The authors could include data presented to this reviewer in their manuscript or add a note to discuss implications of these preliminary findings to improve the clarity and interpretation of results in the presented work." I leave it to your discretion whether to do new experiments, include new data, or clarify in the text which issues have been definitively addressed here vs. which need to be addressed in future studies. I would return the manuscript specifically to Reviewer 3 for evaluation and I would request expedited review. I hope that you will be able to carry these out because I would like to be able to accept your paper, depending on further comments from Reviewer 3.

We are aware that you may be experiencing disruption to the normal running of your lab that makes experimental revisions challenging. If it would be helpful, we encourage you to contact us to discuss your revision in greater detail. Please send us a point-by-point response indicating where you are able to address concerns raised (either experimentally or by changes to the text) and where you will not be able to do so within the normal timeframe of a revision. We will then provide further guidance. Please also note that we are happy to extend revision timeframes as necessary.

Please ensure that you clearly highlight all changes made in the revised manuscript. Please avoid using 'Tracked changes' in Word files as these are lost in PDF conversion.

I should be grateful if you would also provide a point-by-point response detailing how you have dealt with the points raised by the reviewers in the 'Response to Reviewers' box. Please attend to all of the reviewers' comments. If you do not agree with any of their criticisms or suggestions please explain clearly why this is so.

Reviewer 1

Advance summary and potential significance to field

This paper reports thrombin-induction of breast cancer cell blebbing, a phenomenon that can be suppressed by low compressive stress and treatment of Yoda-1, an agonist of mechanosensitive ion channel PIEZO1.
These observations provide a new angle to understand how cell blebbing, an integral characteristic of amoeboid cell state, can be regulated by both chemical and mechanical cues.

Comments for the author

The authors have satisfactorily addressed all my concerns. I appreciate their efforts in revising the manuscript and now support publication.

Reviewer 2

Advance summary and potential significance to field

Please see my original review.

Comments for the author

The authors have addressed satisfactorily all my queries. As such, this reviewer finds the manuscript acceptable for publication in JCS.

© 2022. Published by The Company of Biologists under the terms of the Creative Commons Attribution License (https://creativecommons.org/licenses/by/4.0/).

Reviewer 3

Advance summary and potential significance to field

The authors provided a detailed response to the reviewer questions and have revised their presented manuscript which now includes further data and an extended discussion. Overall, the work presents interesting new data on the role of thrombin and Piezo1 in regulating cell blebbing in MDA-MB-231 breast cancer cells, which can have relevant implications for the regulation of cancer cell migration modes and metastatic potential. As a weakness of the study, the proposed link between Piezo1 activation and mechanical cell confinement however remains unclear. Data in the manuscript are presented in a way that suggests that gentle contact compression may involve Piezo1 activation to suppress thrombin induced cell blebbing, but this notion should be argued with care as no direct evidence is provided.

Comments for the author

Specifically, the authors find that Piezo1 activation and interference with Piezo1 affects thrombin induced cell blebbing. They observe that also gentle contact compression leads to an attenuation of thrombin-induced cell blebbing, providing a potential link between Piezo1 activation and mechanical contact compression.
However, the authors did not directly assess whether gentle contact compression 1) involves mechanosensitive channels (e.g. via gadolinium or other inhibitors of mechanosensitive ion channels) and Piezo1 siRNA depletion was apparently not sufficient to reduce thrombin-induced blebbing in confinement, 2) if a rise in calcium levels is required upon mechanical cell confinement to attenuate cell blebbing (e.g. via addition of an intracellular calcium chelator), and 3) if a change of ERM protein phosphorylation occurs during mechanical contact compression that was suggested to attenuate thrombin-induced blebbing independently of myosin II phosphorylation upon Piezo1 activation via Yoda1.
The authors could include data presented to this reviewer in their manuscript or add a note to discuss implications of these preliminary findings to improve the clarity and interpretation of results in the presented work.

Furthermore, it would be interesting to further discuss how gentle contact compression (an apparently weak mechanical stimulus) can lead to the potential activation of mechanosensitive ion channels or whether other pathways might be involved.
Along this line, the authors could also address the role of other potential mechanisms as changes in membrane mechanics due to PIP2 hydrolysis in regulating thrombin-induced cell blebbing.

Second revision

Author response to reviewers' comments

Reviewer 3 Comments for the Author:

Specifically, the authors find that Piezo1 activation and interference with Piezo1 affects thrombin inducedcell blebbing. They observe that also gentle contact compression leads to an attenuation of thrombin-inducedcell blebbing, providing a potential link between Piezo1 activation and mechanical contact compression.

However, the authors did not directly assess whether gentle contact compression 1) involves mechanosensitive channels (e.g. via gadolinium or other inhibitors of mechanosensitive ion channels) andPiezo1 siRNA depletion was apparently not sufficient to reduce thrombin-induced blebbing in confinement, 2)
if a rise in calcium levels is required upon mechanical cell confinement to attenuate cell blebbing (e.g. viaaddition of an intracellular calcium chelator), and 3) if a change of ERM protein phosphorylation occursduring mechanical contact compression that was suggested to attenuate thrombin-induced blebbing independently of myosin II phosphorylation upon Piezo1 activation via Yoda1.

© 2022. Published by The Company of Biologists under the terms of the Creative Commons Attribution License (https://creativecommons.org/licenses/by/4.0/).

The authors could include data presented to this reviewer in their manuscript or add a note to discussimplications of these preliminary findings to improve the clarity and interpretation of results in the presented work.

Author's response:

We appreciate the opportunity to address these issues:

(1) As suggested by the reviewer we have now added data previously included only in our response text to the reviewer, detailing our inconclusive attempts to assess bleb attenuation by contact compression in Piezo1- depleted cells (now please see new panels Fig. S4F and G, and the related supplementary legend). In the Results (page 7; lines 252-259) we suggest that the similar degree of Ca^{2+} influx seen in siCtrl and siPiezo1 may be due to compensatory activation of additional mechanosensitive channels, which are not engaged during the Piezo1- specific activation achieved with Yoda1. We write that *"Nonetheless, as we could not assess the effects of compression in Piezo1-depleted cells, we have yet to determine whether Piezo1 activation alone is sufficient to mediate the bleb-attenuation observed in response to contact."* which we hope the reviewer agrees improves clarityand permits more nuanced interpretations of the work.

(2) As described in our previous response, our efforts to alter intracellular calcium concentrations using thapsigargin yielded inconclusive results in part due to the activation of store-operated calcium entry mechanisms (previously described for this cell line), resulting in an elevation of cytosolic Ca^{2+} levels. As this elevation in Ca^{2+} was not clearly associated with an increase or decrease in blebbing behavior, we did not pursuethis method in combination with contact compression, and have decided not to add these data to the manuscript, as they do not contribute to a greater understanding of the processes described. With the data added during the previous revision, pertaining to the TRPV4 and TRPM7 channels, we do demonstrate and discuss how Ca^{2+} influx alone is not sufficient to attenuate thrombin-induced blebbing. As discussed below, further work will be required to comprehensively deconstruct the contact-induced mechanisms that contribute to bleb attenuation, butwe hope the reviewer agrees that while we cannot exclusively connect the contact-compression effects to Piezo1activation alone, we provide convincing evidence that it is nonetheless involved in this process, and in this latestrevision have further discussed alternative interpretations and highlighted limitations of our work (as detailed above; page 7, lines 252-259, and below; page 11, lines 387-390).

(3) While we agree that it would be interesting to assess ERM phosphorylation following contact compression, we respectfully suggest that this and other questions raised during our study warrant further investigation in future work. As described above in (1) we have now highlighted that contact compression likely engages additional mechanosensitive channels, and that while Yoda1-activation of Piezo1 is sufficient to attenuate blebbing, contact-attenuation of blebbing is likely a more complex event activating additional mechanisms, andelucidating the roles and activity of ERM and myosin during different compression states would be better achieved by employing alternative methods such as genetically encoded biosensors that permit live imaging of these dynamic events (e.g. the newly developed conformation-sensitive ERM biosensors (Leguay et al., 2021)).

Furthermore, it would be interesting to further discuss how gentle contact compression (an apparently weak mechanical stimulus) can lead to the potential activation of mechanosensitive ion channels or whether other pathways might be involved.

Author's response:
In comparison to the deformation compression that alters the shape of the nucleus, contact compression is a comparatively weaker mechanical stimulus. However, contact compression will engage a relatively large portionof the exposed cell surface, exerting force and increasing plasma membrane tension. Experiments using other mechanical stimuli such as shear flow (Ranade et al., 2014) and patching or poking (Moroni et al., 2018), which are also comparably mild (e.g. do not lead to nucleus deformation) are also sufficient to activate Piezo1.
However, alternative pathways may of course be involved; for example, changes in PM tension alters activity of the kinase TORC2, in part through a PIP2-dependent mechanism (Berchtold et al., 2012, Riggi et al., 2018) and the contact-induced changes studied here may similarly alter the activity of kinases and/or phosphatases involvedin regulating actomyosin contractility. We have now cited these papers and discussed how alternative (Piezo1- independent) pathways such as these should also be considered (page 11, lines 387-390).

© 2022. Published by The Company of Biologists under the terms of the Creative Commons Attribution License (https://creativecommons.org/licenses/by/4.0/).

Along this line, the authors could also address the role of other potential mechanisms as changes inmembrane mechanics due to PIP2 hydrolysis in regulating thrombin-induced cell blebbing.

Author's response:
We have now briefly extended our discussion to include another potential role of PIP2 hydrolysis in thrombin-induced blebbing. This is encompassed in the new brief discussion point described above (page 11, lines 387- 390) where we cite (Berchtold et al., 2012, Riggi et al., 2018).

References

Berchtold, D., Piccolis, M., Chiaruttini, N., Riezman, I., Riezman, H., Roux, A., Walther, T. C. & Loewith, R. (2012). Plasma membrane stress induces relocalization of Slm proteins and activation of TORC2 topromote sphingolipid synthesis. *Nature Cell Biology*, **14**, 542-547.

Leguay, K., Decelle, B., He, Y. Y., Pagniez, A., Hogue, M., Kobayashi, H., Le Gouill, C., Bouvier, M. & Carréno, S. (2021). Development of conformational BRET biosensors that monitor ezrin, radixin and moesin activation in real time. *Journal of Cell Science,* **134**.

Moroni, M., Servin-Vences, M. R., Fleischer, R., Sánchez-Carranza, O. & Lewin, G. R. (2018). Voltage gating ofmechanosensitive PIEZO channels. *Nature Communications,* **9,** 1096.

Ranade, S. S., Qiu, Z., Woo, S.-H., Hur, S. S., Murthy, S. E., Cahalan, S. M., Xu, J., Mathur, J., Bandell, M.,Coste, B., et al. (2014). Piezo1, a mechanically activated ion channel, is required for vascular development in mice. *Proceedings of the National Academy of Sciences,* **111,** 10347-10352.

Riggi, M., Niewola-Staszkowska, K., Chiaruttini, N., Colom, A., Kusmider, B., Mercier, V., Soleimanpour, S.,Stahl, M., Matile, S., Roux, A., et al. (2018). Decrease in plasma membrane tension triggers PtdIns(4,5)P2 phase separation to inactivate TORC2. *Nature Cell Biology,* **20,** 1043-1051.

Third decision letter

MS ID#: JOCES/2021/258809

MS TITLE: Piezo1 activation attenuates thrombin-induced blebbing in breast cancer cells

AUTHORS: Paul O'Callaghan, Adam Engberg, Olle Eriksson, Nikos Fatsis-Kavalopoulos, Christina Stelzl, Gonzalo Sanchez, Olof Idevall-Hagren, and Johan Kreuger
ARTICLE TYPE: Research Article

I am happy to tell you that your manuscript has been accepted for publication in Journal of Cell Science, pending standard ethics checks.

Reviewer 3

Advance summary and potential significance to field

The study presents an interesting link between chemical and mechanical signals that control cell blebbing.
The results stimulate future work to identify the specific mechanosensing pathways and role of calcium signalling in thrombin-induced cell blebbing and its modulation by cell confinement.

Comments for the author

The authors have satisfactorily revised the manuscript and answered to this reviewer.

© 2022. Published by The Company of Biologists under the terms of the Creative Commons Attribution License (https://creativecommons.org/licenses/by/4.0/).
